# Synthesis and anticancer activity of podophyllotoxin derivatives with nitrogen-containing heterocycles

**DOI:** 10.3389/fchem.2023.1191498

**Published:** 2023-05-10

**Authors:** Meng Yin, Yongsheng Fang, Xiaotong Sun, Minggao Xue, Caimei Zhang, Zhiyun Zhu, Yamiao Meng, Lingmei Kong, Yi Yi Myint, Yan Li, Jingfeng Zhao, Xiaodong Yang

**Affiliations:** ^1^ Key Laboratory of Medicinal Chemistry for Natural Resource, Ministry of Education, Yunnan Provincial Center for Research & Development of Natural Products, School of Pharmacy, Yunnan University, Kunming, China; ^2^ Department of Chemistry, University of Mandalay, Mandalay, Myanmar

**Keywords:** podophyllotoxin, imidazolium salts, triazoles, antitumor activity, structure-activity relationships

## Abstract

Three series of podophyllotoxin derivatives with various nitrogen-containing heterocycles were designed and synthesized. The antitumor activity of these podophyllotoxin derivatives was evaluated *in vitro* against a panel of human tumor cell lines. The results showed that podophyllotoxin-imidazolium salts and podophyllotoxin-1,2,4-triazolium salts **a1–a20** exhibited excellent cytotoxic activity. Among them, **a6** was the most potent cytotoxic compound with IC_50_ values of 0.04–0.29 μM. Podophyllotoxin-1,2,3-triazole derivatives **b1–b5** displayed medium cytotoxic activity, and podophyllotoxin-amine compounds **c1–c3** has good cytotoxic activity with IC_50_ value of 0.04–0.58 μM. Furthermore, cell cycle and apoptosis experiments of compound **a6** were carried out and the results exhibited that **a6** could induce G2/M cell cycle arrest and apoptosis in HCT-116 cells.

## 1 Introduction

According to the data from the International Agency for Cancer Research (IARC), there would be around 19.3 million new cancer diagnoses and nearly 10 million cancer-related deaths in 2020 (Sung et al., 2021). Therefore, the development of innovative anticancer agents and therapeutic strategies is essential (Boshuizen and Peeper, 2020). Medicinal chemists have increasingly viewed natural products as valuable resources for developing anticancer drug (Choi et al., 2017). About 84% of antitumor small molecule drugs approved between 1981 and 2019 were derived from natural products or structural units containing natural products (Newman and Cragg, 2020). The design and rational synthesis of natural product-like libraries, from which lead compounds with high efficiency, high selectivity, and low toxicity can be screened and discovered for preclinical studies, is one of the significant approaches for developing new drugs (Liu et al., 2017).

Podophyllotoxin is a natural product with anticancer activity belonging to the lignans cyclolignolide family (Dagenais et al., 2020). Podophyllotoxin and its semi-synthetic glycoside derivatives Etoposide, Teniposide and Etoposide Phosphate have been proved to be highly active antitumor agents with excellent clinical effects and are essential drugs for the treatment of small cell lung cancer, leukemia, testicular cancer and other types of tumors (Zhang et al., 2018; Li et al., 2019; Guo and Jiang, 2021; Zhao et al., 2021). Numerous structural and pharmacological studies have demonstrated that C-4 derivatization could enhance the biological activity of this family of drugs (Xiao et al., 2020).

**FIGURE 1:**
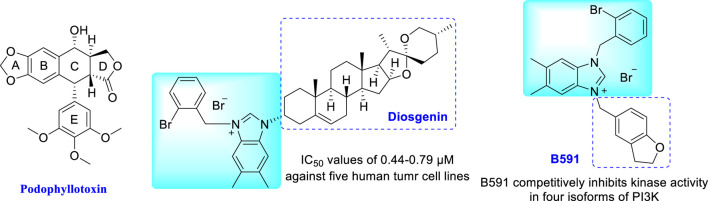
Structures of podophyllotoxin and representative imidazolium salts with antitumor activity.

On the other side, nitrogen-containing heterocycles are widely used in drug design and discovery (Xu et al., 2014a; Vitaku et al., 2014). The unique structural features of imidazoles and triazoles possess desirable electron rich properties, which are more favorable for conjugation with other molecules, and the molecular activity could be improved after hybridization (Verma et al., 2013; Gaba and Mohan, 2015; Bozorov et al., 2019; Xu et al., 2019; Dixit et al., 2021; Sharma et al., 2021). Among them, imidazolium salts have attracted much attention for their important and extensive biological and pharmacological activities, especially antitumor activity (Cui et al., 2003; Yang et al., 2009). In this context, our group has devoted to the synthesis of novel imidazolium salt derivatives and found a series of promising compounds with antitumor activity (Chen et al., 2013; Wang et al., 2013; Xu et al., 2014b; Xu et al., 2015; Zhou et al., 2016a; Zhou et al., 2016b). Further mechanistic studies confirmed that these imidazolium salt derivatives can induce cell cycle arrest and apoptosis in tumor cells (Liu et al., 2013; Liu et al., 2015; Huang et al., 2019). The representative examples are an effective antitumor active diosgenin-imidazolium salt and a new mTOR pathway inhibitor **B591** (Figure 1) (Deng et al., 2019; Zhou et al., 2019).

In the past three decades, molecular hybridization has played an important role in drug discovery (Zhang et al., 2017; Yang et al., 2021). In view of the potential anticancer activity of podophyllotoxin and nitrogen-containing heterocycles, we launched the synthesis of hybrid compounds of natural product podophyllotoxin and imidazolium/triazolium salts. Although some nitrogen-containing heterocycles-podophyllotoxin derivatives were prepared and found to possess anticancer and neuroactive activities (Chen et al., 2011; Shang et al., 2012; Vishnuvardhan et al., 2017; Hou et al., 2019), to the best of our knowledge, there are no reports on the synthesis and bioactivity of imidazolium/triazolium salt hybrids of podophyllotoxin. With this in mind, we turned our attention to the synthesis and antitumor activity of a series of novel podophyllotoxin nitrogen-containing heterocycles, especially imidazolium and triazolium salts.

## 2 Results and discussion

### 2.1 Chemistry

**SCHEME 1:**
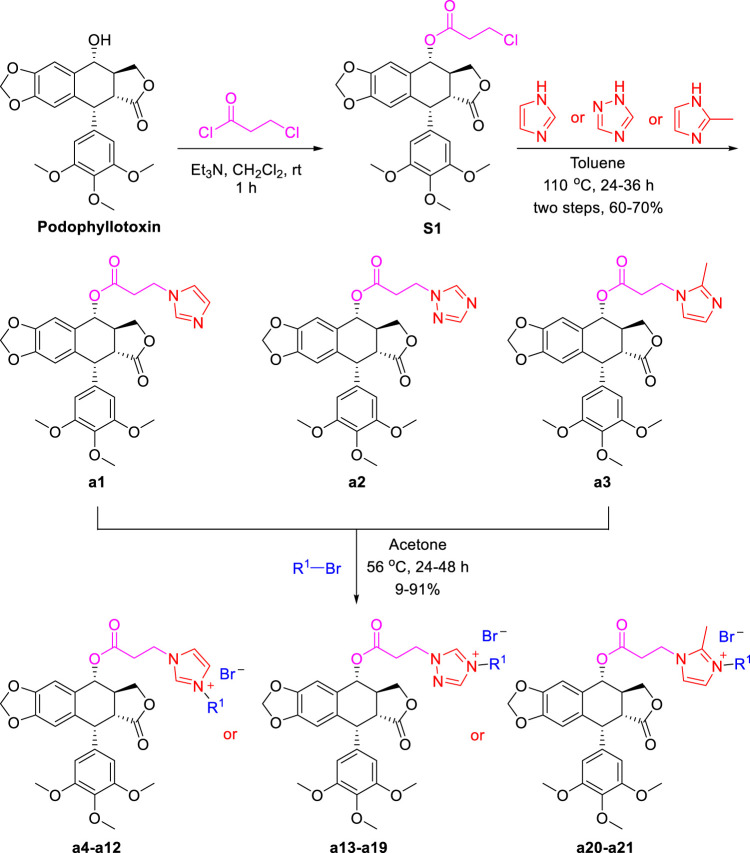
Synthesis of podophyllotoxin nitrogenous derivatives **a1–a21**.

**SCHEME 2:**
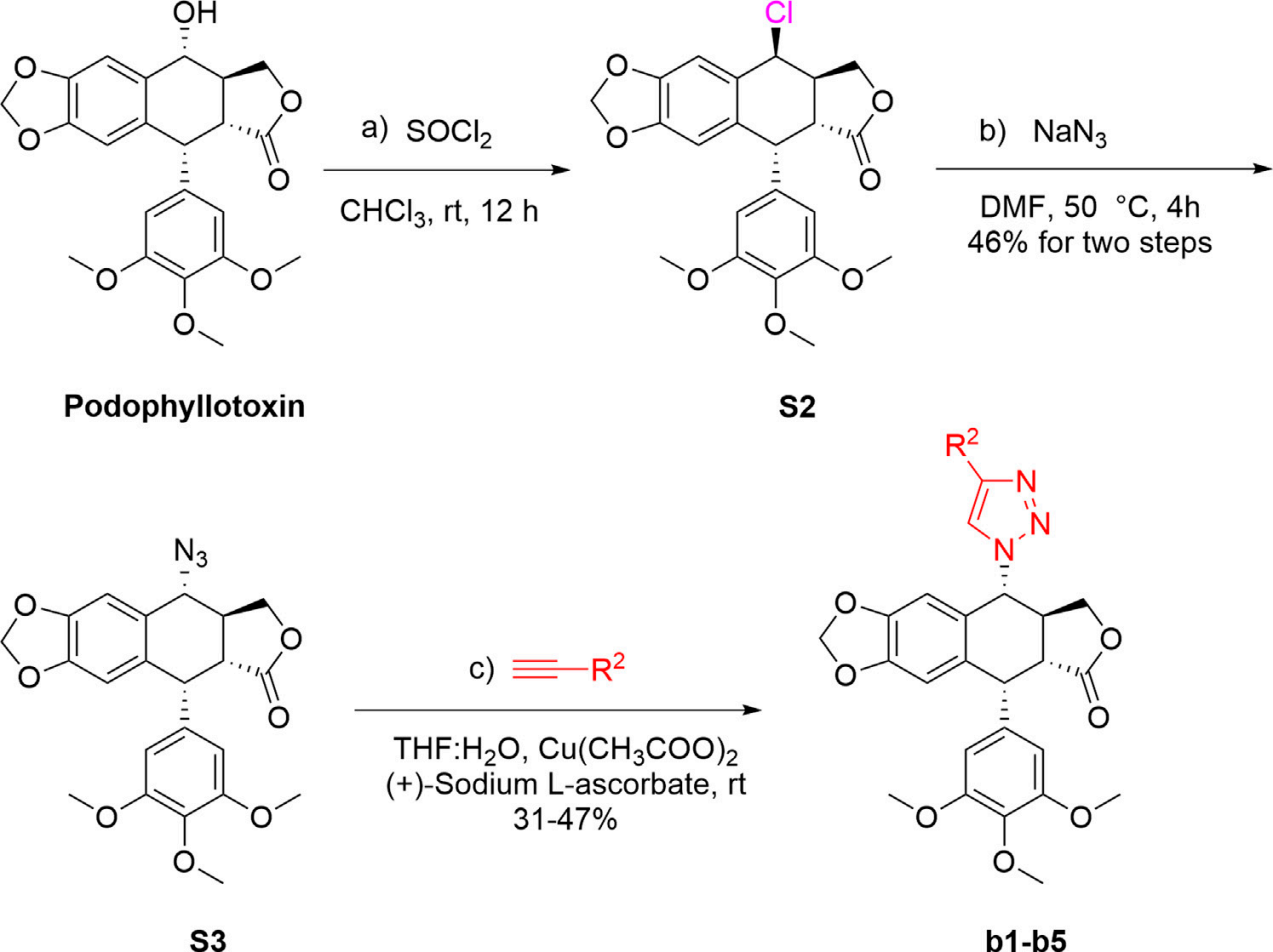
Synthesis of podophyllotoxin nitrogenous derivatives **b1–b5**.

**SCHEME 3:**
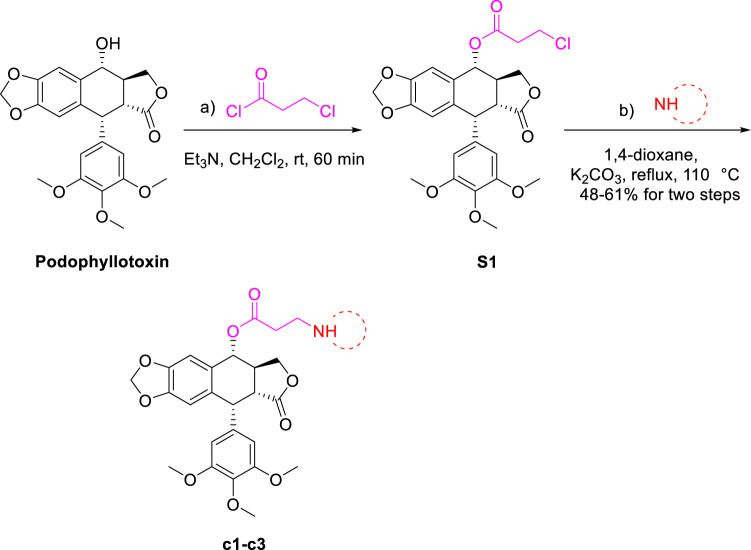
Synthesis of podophyllotoxin nitrogenous derivatives **c1–c3**.

As shown in Scheme 1, firstly, to synthesize podophyllotoxin nitrogen-containing heterocycles, imidazole, 1,2,4-triazole, 2-methylimidazole, 1,2,3-triazole and amines were used for reaction. Using the commercial podophyllotoxin as starting material, the esterification reaction with 2-chloropropionyl chloride was carried out to obtain the ester **S1**. Next, **S1** reacted with imidazole, 1,2,4-triazole and 2-methylimidazole to obtain the nitrogen-containing heterocycles **a1–a3** (60%–70% yields, two steps). Then, treatment of **a1–a3** with various bromides generated the podophyllotoxin imidazolium/triazolium salts **a4–a21** (9%–91% yields). Secondly, as shown in Scheme 2, 4-chlorinated podophyllotoxin **S2** was obtained by commercial podophyllotoxin reacting with thionyl chloride. Next, a nucleophilic substitution reaction with sodium azide was conducted to obtain compound **S3** (46% yield, two steps). Then, azide **S3** reacted with various terminal alkynes under Click reaction condition to get the podophyllotoxin-1,2,3-triazole derivatives **b1–b5** (31%–47% yields). Finally, as shown in Scheme 3, using podophyllotoxin as the starting material, esterification reaction with 2-chloropropionyl chloride was performed to obtain the ester **S1**, which then underwent a nucleophilic substitution reaction with commercial cyclic amines (pyrrole, piperidine and morpholine) to furnish the podophyllotoxin-amines **c1-c3** (48%–61% yields, two steps). To summarize, the structures and yields of all new podophyllotoxin nitrogen-containing heterocycle derivatives were shown in Table 1.

**TABLE 1:** Structures and yields of podophyllotoxin nitrogen-containing heterocycles **a1–a21/b1–b5/c1–c3**.

Entry	Compound	R^1^	R^2^	R^3^	Yields (%)
1	**a1**	—	—	—	68
2	**a2**	—	—	—	70
3	**a3**	—	—	—	60
4	**a4**	2-naphthylacyl	—	—	38
5	**a5**	4-bromophenacyl	—	—	91
6	**a6**	2-naphthylmethyl	—	—	58
7	**a7**	phenacyl	—	—	44
8	**a8**	4-methoxyphenacyl	—	—	53
9	**a9**	4-bromobenzy	—	—	82
10	**a10**	4-methylbenzyl	—	—	46
11	**a11**	2-bromobenzyl	—	—	81
12	**a12**	5-bromomethyl	—	—	78
13	**a13**	2-naphthylacyl	—	—	30
14	**a14**	4-bromophenacyl	—	—	34
15	**a15**	2-naphthylmethyl	—	—	63
16	**a16**	phenacyl	—	—	37
17	**a17**	4-methoxyphenacyl	—	—	80
18	**a18**	4-bromobenzy	—	—	71
19	**a19**	4-methylbenzyl	—	—	9
20	**a20**	4-bromophenacyl	—	—	58
21	**a21**	phenacyl	—	—	50
22	**b1**	—	F	—	31
23	**b2**	—	Br	—	47
24	**b3**	—	OMe	—	33
25	**b4**	—	Pyridine	—	38
26	**b5**	—	Naphthalene	—	43
27	**c1**	—	—	Pyrrolidine	58
28	**c2**	—	—	Piperidine	61
29	**c3**	—	—	Morpholine	48

### 2.2 Biological evaluation and structure-activity relationship analysis

#### 2.2.1 Biological assay procedures and results

The synthesized twenty-nine podophyllotoxin nitrogen-containing derivatives were evaluated *in vitro* antitumor cytotoxic activity screening by MTS method (Perchellet et al., 2005). Four human cancer cell lines including hepatocellular carcinoma cells (HepG-2), non-small cell lung cancer cells (A-549), breast cancer cells (MDA-MB-231) and colon cancer cells (HCT-116) were selected to determine *in vitro* cytotoxic activity. DDP (Cisplatin), Etoposide, and Paclitaxel were chosen as positive controls. The results were listed in Table 2.

**TABLE 2:** Cytotoxic activities of podophyllotoxin nitrogen-containing heterocycles **a1–a21**/**b1–b5**/**c1–c3** in *vitro* ^a^ (IC_50_, μM^b^).

Entry	Compound No.	HepG-2	A-549	MDA-MB-231	HCT-116
1	**a1**	0.31 ± 0.02	0.76 ± 0.12	0.47 ± 0.01	0.04 ± 0.00
2	**a2**	0.23 ± 0.01	0.30 ± 0.02	0.45 ± 0.03	0.15 ± 0.01
3	**a3**	0.32 ± 0.06	0.65 ± 0.03	0.38 ± 0.04	0.31 ± 0.04
4	**a4**	0.33 ± 0.01	1.11 ± 0.04	0.53 ± 0.01	0.04 ± 0.00
5	**a5**	0.29 ± 0.00	0.76 ± 1.54	0.51 ± 0.05	0.04 ± 0.00
6	**a6**	0.07 ± 0.00	0.29 ± 0.04	0.11 ± 0.01	0.04 ± 0.00
7	**a7**	0.18 ± 0.01	1.08 ± 0.20	0.48 ± 0.02	0.04 ± 0.00
8	**a8**	0.26 ± 0.01	0.65 ± 0.39	0.55 ± 0.02	0.04 ± 0.00
9	**a9**	0.25 ± 0.01	0.44 ± 0.10	0.49 ± 0.02	0.29 ± 0.00
10	**a10**	0.25 ± 0.01	0.25 ± 0.00	0.45 ± 0.09	0.30 ± 0.05
11	**a11**	0.27 ± 0.00	0.42 ± 0.09	0.33 ± 0.06	0.30 ± 0.08
12	**a12**	0.26 ± 0.05	0.53 ± 0.10	0.51 ± 0.02	0.21 ± 0.01
13	**a13**	0.34 ± 0.02	1.10 ± 0.14	0.41 ± 0.06	0.10 ± 0.02
14	**a14**	0.42 ± 0.03	1.53 ± 0.23	0.33 ± 0.04	0.05 ± 0.02
15	**a15**	0.29 ± 0.01	0.75 ± 0.07	0.30 ± 0.00	0.27 ± 0.15
16	**a16**	0.28 ± 0.02	0.75 ± 0.07	0.25 ± 0.02	0.20 ± 0.06
17	**a17**	0.25 ± 0.02	0.74 ± 0.19	0.28 ± 0.05	0.04 ± 0.09
18	**a18**	0.28 ± 0.03	0.58 ± 0.01	0.23 ± 0.02	0.04 ± 0.00
19	**a19**	0.25 ± 0.00	0.65 ± 0.01	0.26 ± 0.00	0.04 ± 0.00
20	**a20**	7.98 ± 0.51	15.84 ± 0.04	>20	6.80 ± 0.11
21	**a21**	0.40 ± 0.03	0.28 ± 0.05	0.11 ± 0.03	0.07 ± 0.01
22	**b1**	1.86 ± 0.15	3.60 ± 0.56	2.03 ± 0.14	0.04 ± 0.33
23	**b2**	2.14 ± 0.04	7.31 ± 0.12	1.74 ± 0.47	6.58 ± 1.87
24	**b3**	1.60 ± 0.00	3.48 ± 0.03	0.49 ± 0.01	0.90 ± 0.42
25	**b4**	4.59 ± 0.37	9.64 ± 0.62	7.57 ± 0.62	1.49 ± 1.76
26	**b5**	>20	>20	>20	>20
27	**c1**	0.28 ± 0.01	0.58 ± 0.02	0.04 ± 0.03	0.05 ± 0.01
28	**c2**	0.10 ± 0.01	0.39 ± 0.03	0.10 ± 0.00	0.10 ± 0.02
29	**c3**	0.21 ± 0.03	0.39 ± 0.01	0.36 ± 0.11	0.06 ± 0.11
30	**DDP**	1.85 ± 0.34	5.52 ± 0.21	12.77 ± 2.71	10.92 ± 0.26
31	**Etoposide**	16.95 ± 2.00	14.77 ± 0.26	1.92 ± 0.96	14.19 ± 0.13
32	**Paclitaxel**	<0.008	<0.008	<0.008	<0.008

^a^ Data represent the mean values of three independent determinations.

^b^ Cytotoxicity as IC_50_ for each cell line, is the concentration of compound which reduced by 50% the optical density of treated cells with respect to untreated cells using the MTS assay.

As presented in Table 2, the majority of podophyllotoxin nitrogen-containing heterocycles showed potent inhibitory activity than positive controls Etoposide and DDP. Notably, these derivatives have obvious selective inhibitory against HCT-116 cell lines. The results showed that the structures of podophyllotoxin nitrogen-containing heterocycles plays a crucial role in regulating cytotoxic activity.

For pharmacophores of nitrogen-containing heterocycles, podophyllotoxin-imidazole and its salts (**a1/a3/a4-a12/a20/a21**) and podophyllotoxin-1,2,4-triazole and its salts (**a2/a13–a19**) exhibited excellent cytotoxic activity with IC_50_ values of 0.04–1.53 μM except **a20**. Among them, **a6** was the most potent cytotoxic compound and its IC_50_ values for HepG2, A-549, MDA-MB-231 and HCT-116 were 0.07, 0.29, 0.11 and 0.04 μM, respectively. Secondly, the introduction of 1,2,3-triazole derivatives **b1–b5** by Click reaction showed medium cytotoxic activity with IC_50_ values of 0.04–9.64 μM except **b5**. Finally, while compounds **c1–c3** introduced with cyclic amines also showed excellent cytotoxic activity with IC_50_ values of 0.04–0.58 μM.

For the groups at position-3 of imidazolium and triazolium salts (**a4–a21**), the cytotoxic activities of most substituted benzyl groups were superior to those of substituted phenacyl groups. Among them, 2-naphthylmethyl substituent at position-3 of the imidazole ring (**a6** and **a15**) showed excellent cytotoxic activity with IC_50_ values of 0.04–0.75 μM and **a6** was the most powerful compound. Similarly, 4-bromobenzyl, 4-methylbenzyl, 4-methoxybenzoyl and 2-bromobenzyl groups at position-3 of the imidazole ring exhibited good cytotoxic activity with IC_50_ values of 0.04–0.65 μM.

For the groups at position-4 of 1,2,3-triazole ring (**b1–b5**), when the substituent was replaced with electron donating groups (R^2^ = OMe), **b3** exhibits higher inhibitory activity with IC_50_ values of 0.49–3.48 μM. In contrast, when the substituent was charged with electron-withdrawing groups (R^2^ = F, Br), **b1** and **b2** were decreased slightly with IC_50_ values of 0.04–7.31 μM. When the substituent group was pyridine, **b4** exhibited poor inhibitory activity with IC_50_ values of 1.49–9.64 μM, due to the electron-withdrawing effect of pyridine. When the substituent was a naphthalene ring, **b5** did not exhibit any inhibitory activity.

For the cyclic amines (**c1–c3**), piperidine derivative of podophyllotoxin (**c2**) displayed excellent cytotoxic activity with IC_50_ values of 0.10–0.39 μM, which was superior to pyrrole derivative (0.04–0.58 μM) and morpholine derivative (0.06–0.39 μM). Notably, compound **c1** has selective inhibitory against MDA-MB-231 cell lines with an IC_50_ value of 0.04 μM.

**SCHEME 4:**
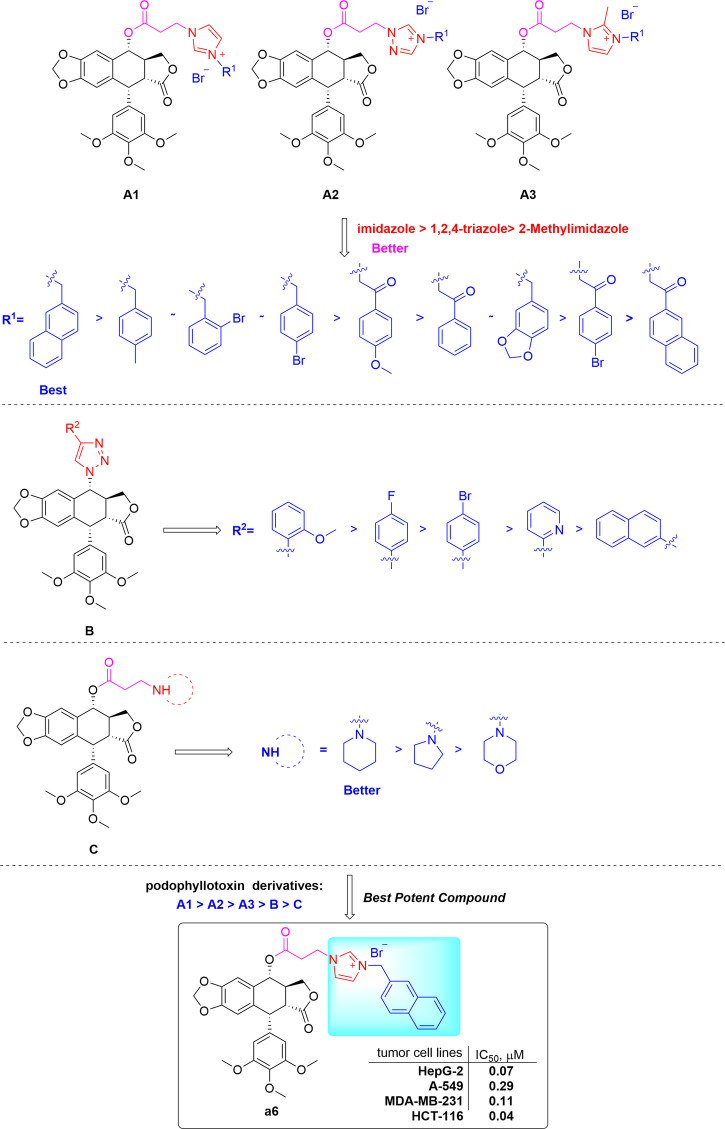
Structure-activity relationship of podophyllotoxin derivatives.

The results demonstrated that the introduction of imidazole ring into podophyllotoxin and a 2-naphthyl methyl substituent at the imidazolium salt’s 3-position play a critical role in enhancing cytotoxic activity. The preliminary structure activity relationships (SARs) of the derivatives were summarized in Scheme 4.

#### 2.2.2 Compound **a6** induced G2/M cell cycle arrest and apoptosis

**FIGURE 2:**
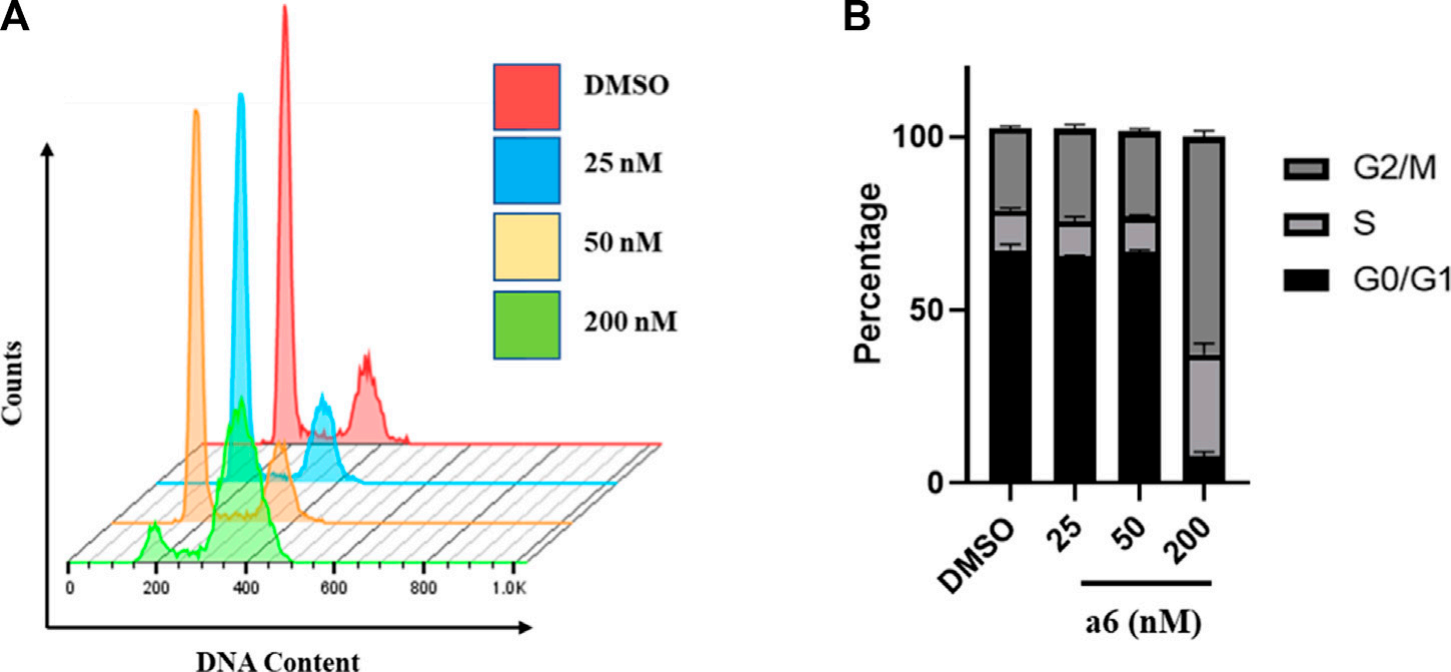
Compound **a6** induced G2/M phase arrest in HCT-116 cells. **(A)** Cells were treated with different concentrations of compound **a6** (25, 50 and 200 nM) for 24 h, and cell cycle was determined by cell cytometry with PI staining. **(B)** The percentages of cells in different phases were quantified.

To determine the possible mechanism of compound **a6** induced proliferation inhibition, cell cycle and apoptosis analysis were performed with flow cytometry. Firstly, HCT-116 cells were treated with indicated concentrations of compound **a6** for 24 h and the cell cycle phase distribution of **a6**-treated cells was determined with propidium iodide (PI) staining. As shown in Figure 2, **a6** exposure caused G2/M phase arrest in HCT-116 cells when compared with the control group, indicating that compound **a6** inhibited cell proliferation through inducing G2/M cell cycle arrest.

**FIGURE 3:**
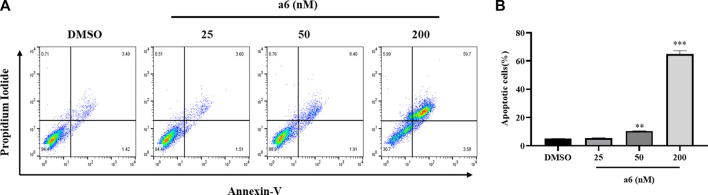
Compound **a6** induced apoptosis of HCT-116 cells. **(A)** Cells were treated with 25, 50 and 200 nM compound **a6** for 48 h. Cell apoptosis was determined by Annexin V-FITC/PI staining analysis. **(B)** The quantification of apoptotic cells.

The compound **a6** induced cell apoptosis was also determined with Annexin V-FITC/PI staining. As shown in Figure 3, after treated with compound **a6** at 25, 50 and 200 nM for 48 h, the apoptotic rate of HCT-116 cells remarkably elevated to 5.37 ± 0.37%, 10.45 ± 0.20% and 64.98 ± 2.40%, respectively. The results suggested that compound **a6** inhibited cell proliferation through induction of G2/M cell cycle arrest and apoptosis of HCT-116 cells.

## 3 Conclusion

In conclusion, a series of novel podophyllotoxin nitrogen-containing heterocycle derivatives with potential antitumor activity were prepared using a straightforward synthetic approach. The results showed that the imidazole-substituted derivatives demonstrated more effective inhibitory activity than 1,2,4-triazole-substituted and 1,2,3-triazole-substituted equivalents. The biological activity was significantly improved when the imidazole or imidazolium salt group was introduced into the structure of podophyllotoxin. Among them, imidazolium salt **a6** was the most potent cytotoxic activity with IC_50_ values of 0.04–0.29 μM. It has an obvious selective inhibitory against HCT-116 cell lines with an IC_50_ value of 0.04 μM and could induce G2/M cell cycle arrest and apoptosis in HCT-116 cells. Podophyllotoxin-imidazolium salt **a6** could be employed as a promising lead compound for further structural modification and in-depth activity research to identify new starting points for more effective anticancer agents.

## Data Availability

The original contributions presented in the study are included in the article/Supplementary Material, further inquiries can be directed to the corresponding authors.
