# Supplementary material for: Synthesis and anticancer activity of podophyllotoxin derivatives with nitrogen-containing heterocycles
Source: Front Chem. 2023 May 10;11:1191498. doi: 10.3389/fchem.2023.1191498 (PMC10206303; doi:10.3389/fchem.2023.1191498)
Supplement: Supplementary file 1 [file DataSheet1.pdf]

Supplementary Material

Synthesis and anticancer activity of podophyllotoxin derivatives with nitrogen-containing heterocycles

Meng Yin[1†], Yongsheng Fang[1†], Xiaotong Sun[1], Minggao Xue[1], Caimei Zhang[1], Zhiyun Zhu[1], Yamiao Meng[1], Lingmei Kong[1], Yi Yi Myint[2], Yan Li[1,*], Jingfeng Zhao[1,*], Xiaodong Yang[1,*]

[1]Key Laboratory of Medicinal Chemistry for Natural Resource, Ministry of Education; Yunnan Provincial Center for Research & Development of Natural Products, School of Pharmacy, Yunnan University, Kunming, 650091, P. R. China.

[2]Department of Chemistry, University of Mandalay, Mandalay, Myanmar.

[†]These authors have contributed equally to this work.

Table of Contents

1. General Experimental.. S2
2. Experimental Procedures and Analytical Data... S3-S30
3. Biological Assay Procedures and Results... S31-S32
4. ^{1}H NMR, ^{13}C NMR and HR-MS (ESI) spectra of New Compounds.............. .... S33-S62

1. General Experimental

Melting points were obtained and uncorrected on a Haineng melting-point apparatus. Proton nuclear magnetic resonance (^{1}H-NMR) spectra were recorded on a Bruker Avance 400 and Bruker Avance 600 spectrometers at 400 MHz and 600 MHz. Carbon-13 nuclear magnetic resonance (^{13}C-NMR) was recorded on Bruker Avance 400 spectrometer at 100 MHz. And carbon-13 nuclear magnetic resonance (^{13}C-NMR) was recorded on Bruker Avance 600 spectrometer at 150 MHz. Chemical shifts are reported as δ values in parts per million (ppm) relative to tetramethylsilane (TMS) for all recorded NMR spectra. High Resolution Mass spectra were taken on Aglient LC-MSO/TOR mass spectrometer. Silica gel (200–300 mesh) for column chromatography and silica GF254 for TLC were produced by Qingdao Marine Chemical Company (China). All air- or moisture- sensitive reactions were conducted under an argon atmosphere. Starting materials and reagents used in reactions were obtained commercially from TCI, Acros, Adamas, Bidepharm and were used without purification, unless otherwise indicated.

2. Experimental Procedures and Analytical Data

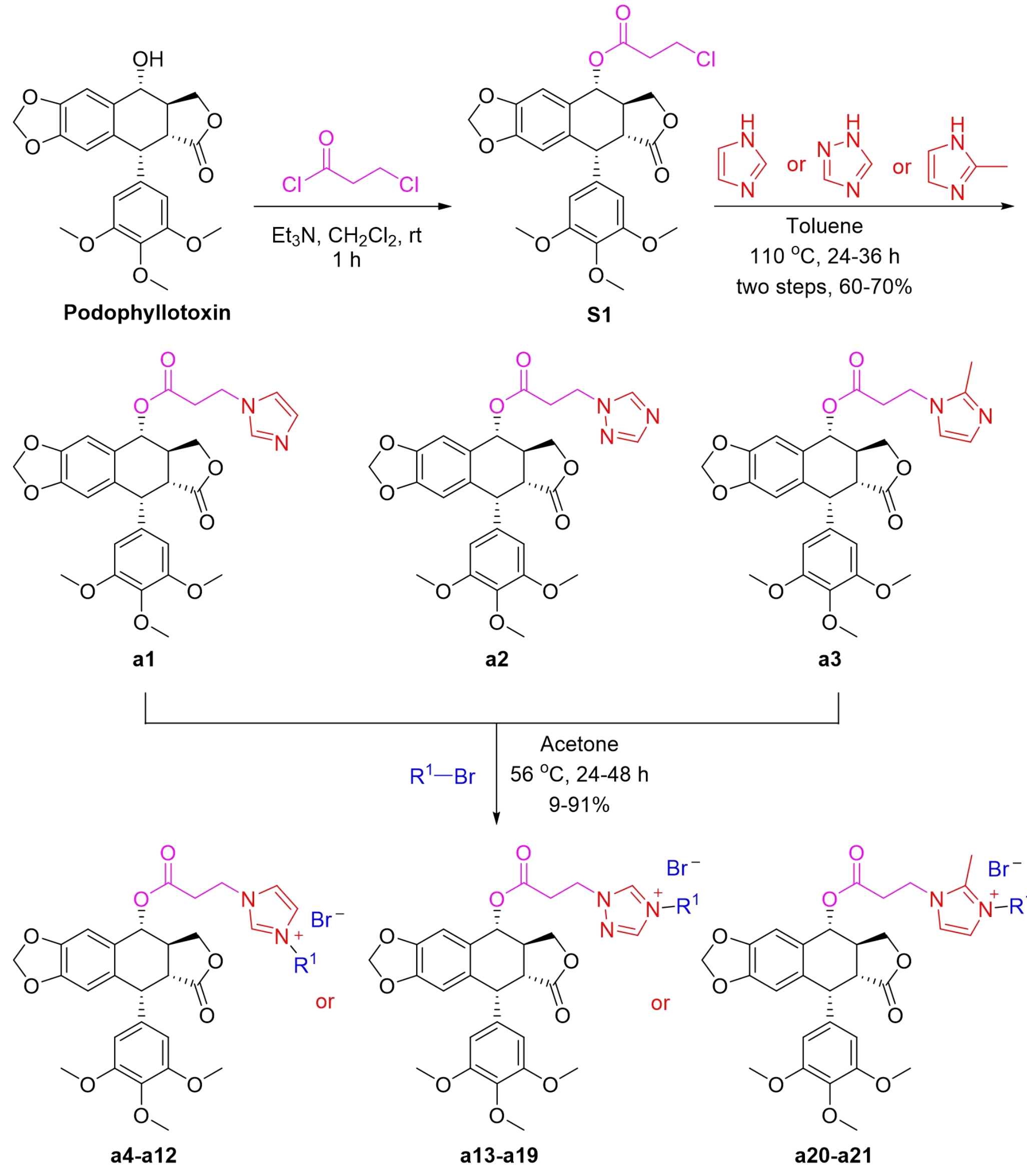

Scheme 1 Synthesis of podophyllotoxin nitrogenous derivatives **a1-a21**.

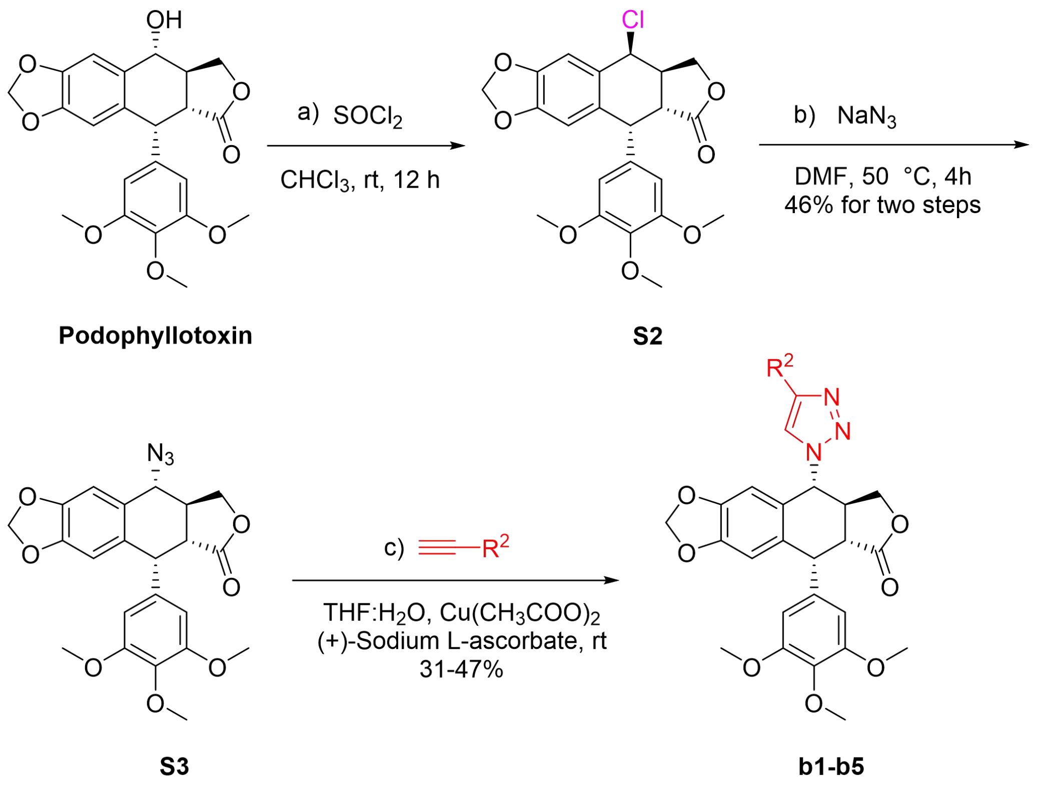

Scheme 2 Synthesis of podophyllotoxin nitrogenous derivatives **b1-b5**.

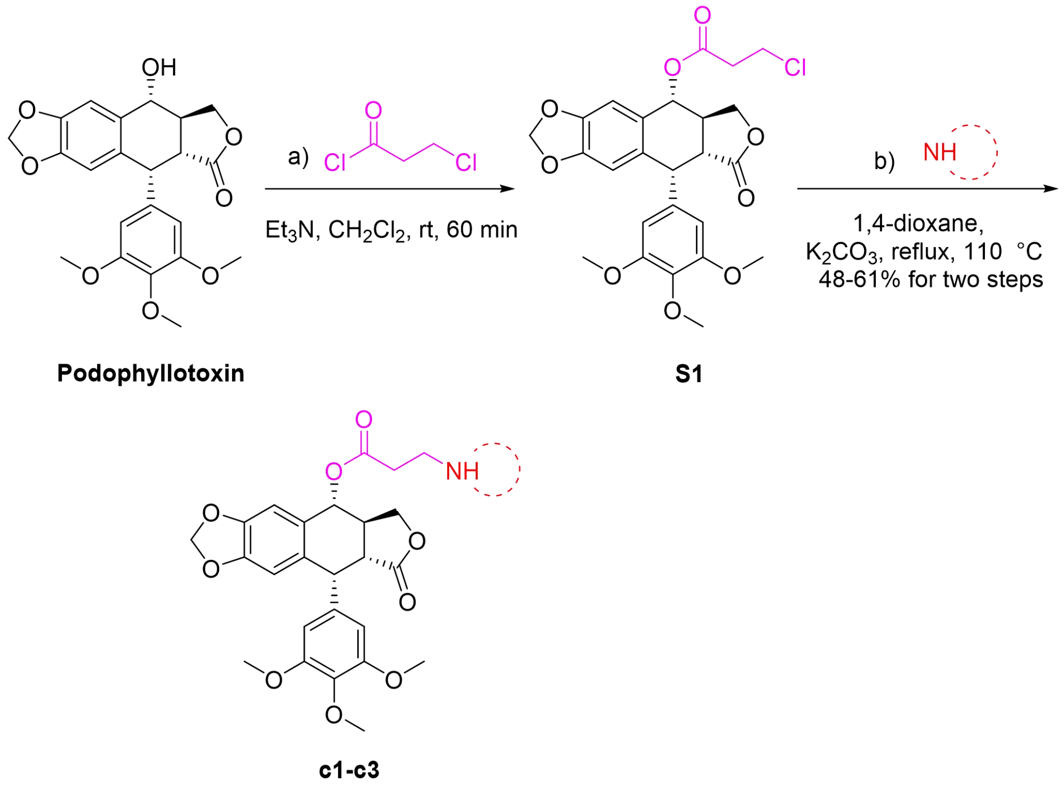

Scheme 3 Synthesis of podophyllotoxin nitrogenous derivatives **c1-c3**.

Table 1

Structures and yields of podophyllotoxin nitrogen-containing heterocycles **a1–a21/b1-b5/c1-c3**.

Entry	Compound	R^1	R^2	R^3	Yields
1	a1	-	-	-	68%
2	a2	-	-	-	70%
3	a3	-	-	-	60%
4	a4	2-naphthylacyl	-	-	38%
5	a5	4-bromophenacyl	-	-	91%
6	a6	2-naphthylmethyl	-	-	58%
7	a7	phenacyl	-	-	44%
8	a8	4-methoxyphenacyl	-	-	53%
9	a9	4-bromobenzy	-	-	82%
10	a10	4-methylbenzyl	-	-	46%
11	a11	2-bromobenzyl	-	-	81%
12	a12	5-bromomethyl	-	-	78%
13	a13	2-naphthylacyl	-	-	30%
14	a14	4-bromophenacyl	-	-	34%
15	a15	2-naphthylmethyl	-	-	63%
16	a16	phenacyl	-	-	37%
17	a17	4-methoxyphenacyl	-	-	80%
18	a18	4-bromobenzy	-	-	71%
19	a19	4-methylbenzyl	-	-	9%
20	a20	4-bromophenacyl	-	-	58%
21	a21	phenacyl	-	-	50%
22	b1	-	F	-	31%
23	b2	-	Br	-	47%
24	b3	-	OMe	-	33%
25	b4	-	pyridine	-	38%
26	b5	-	naphthalene	-	43%
27	c1	-	-	pyrrolidine	58%
28	c2	-	-	piperidine	61%

**29**	**c3**	-	-	morpholine	48%

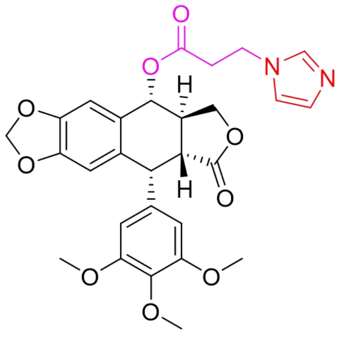

a1(68%)

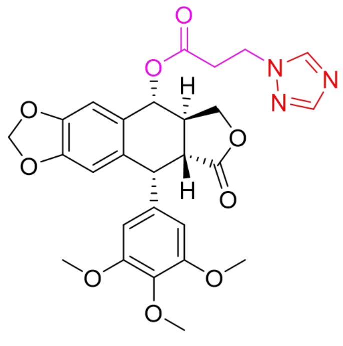

a2(70%)

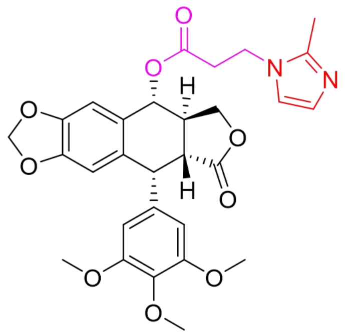

a3(60%)

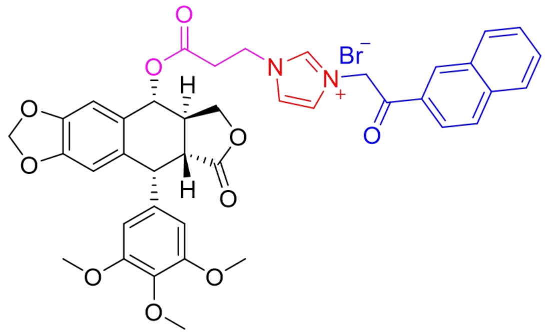

a4(38%)

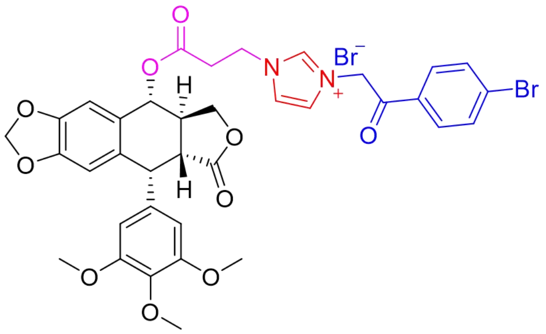

a5(91%)

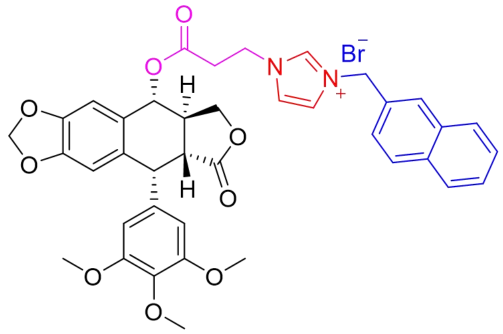

a6(58%)

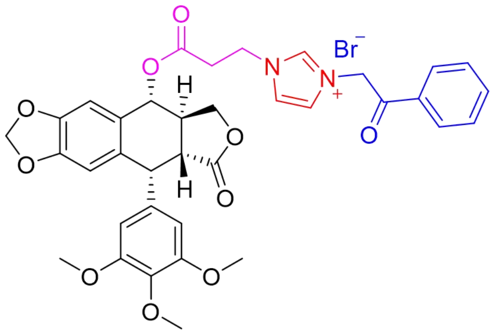

a7(44%)

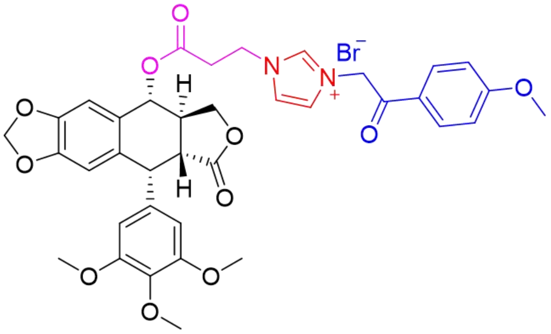

a8(53%)

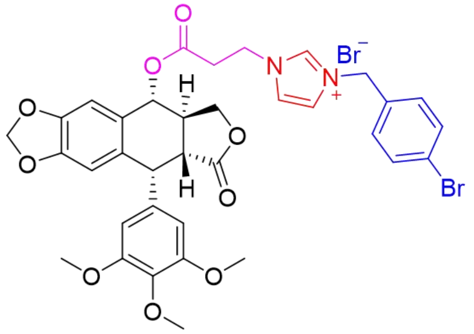

a9(82%)

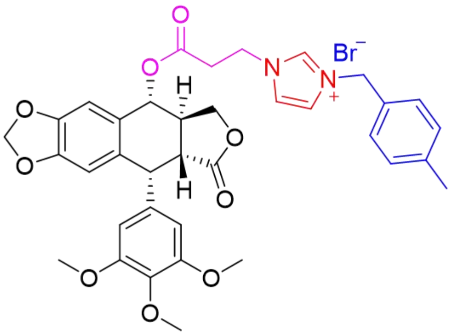

a10(46%)

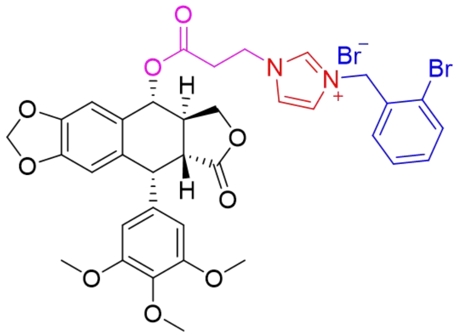

a11(81%)

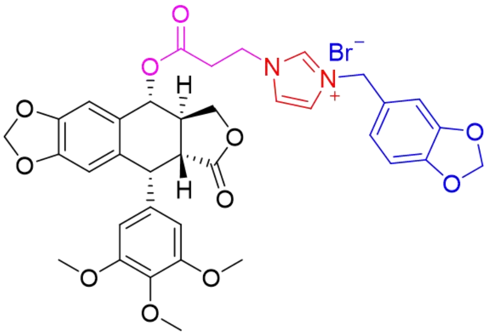

a12(78%)

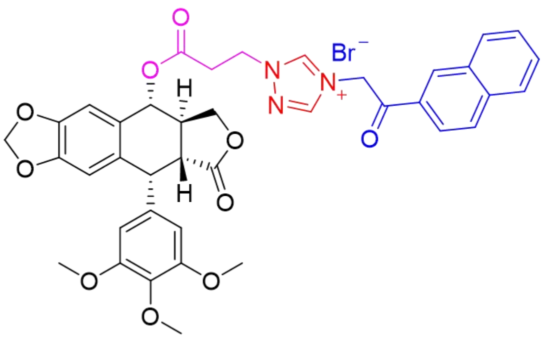

a13(30%)

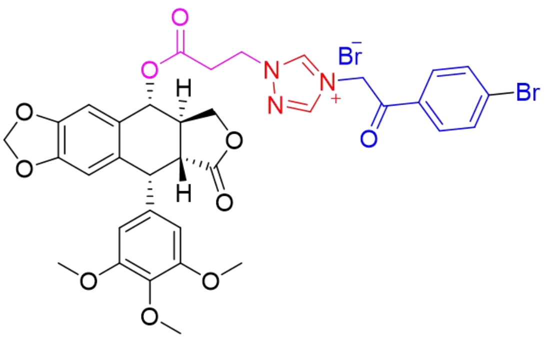

a14(34%)

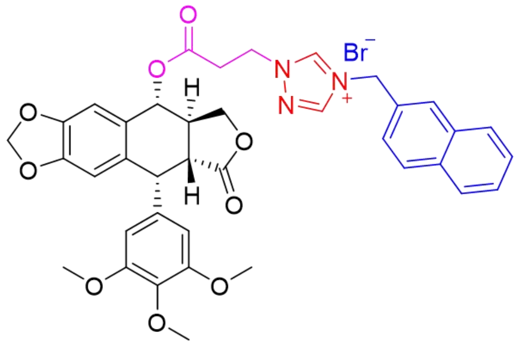

a15(63%)

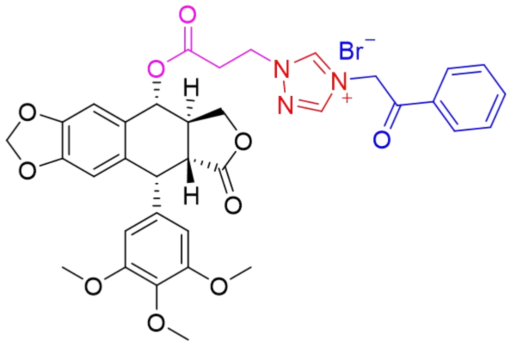

a16(37%)

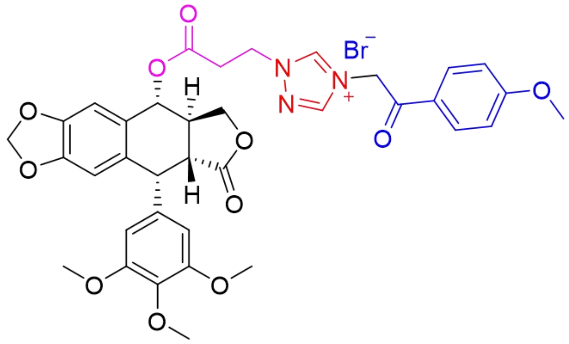

a17(80%)

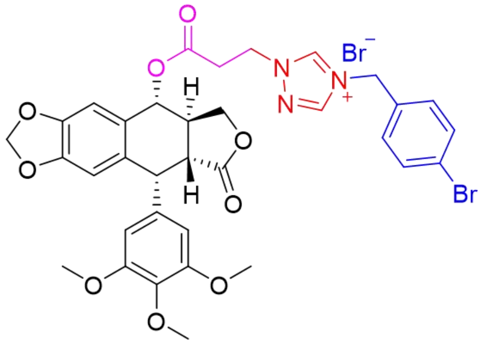

a18(71%)

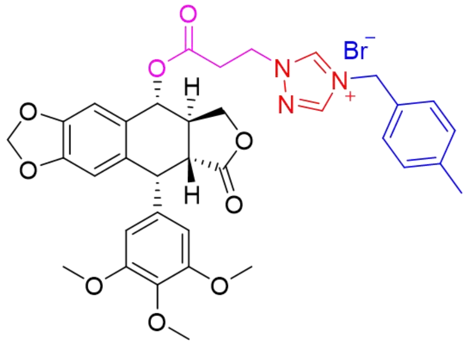

a19(9%)

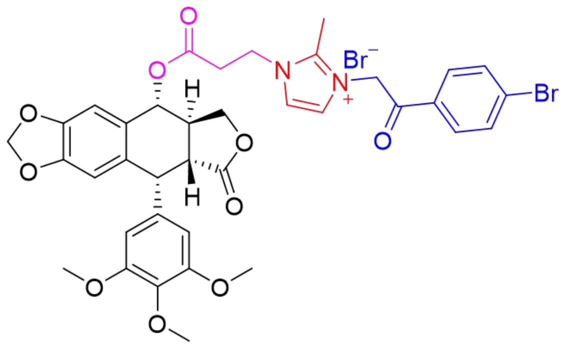

a20(58%)

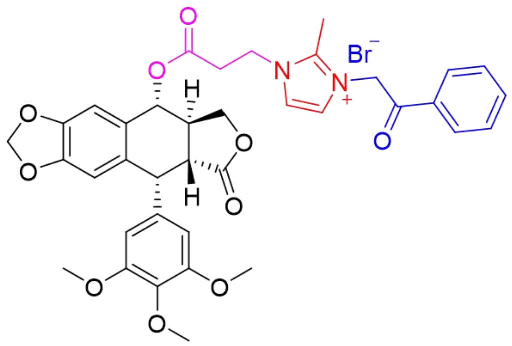

a21(50%)

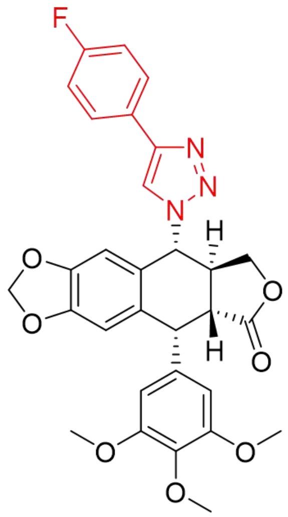

b1(31%)

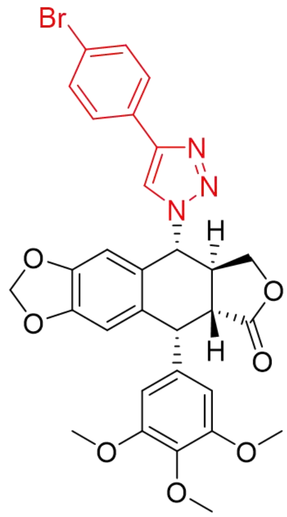

b2(47%)

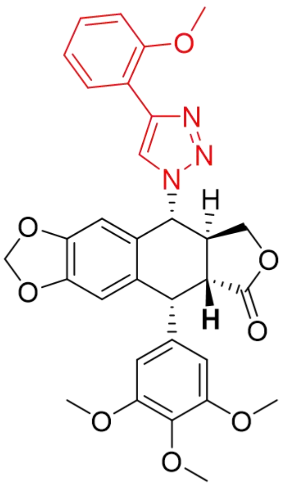

b3(33%)

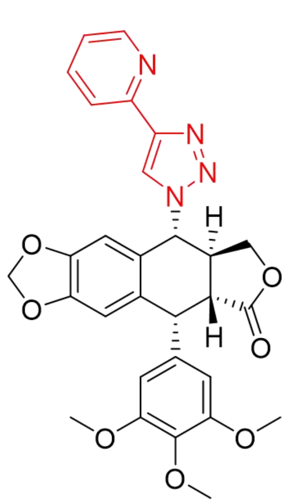

b4(38%)

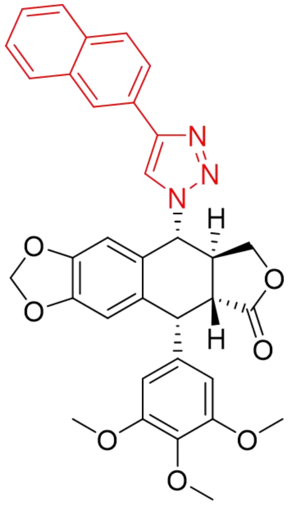

b5(43%)

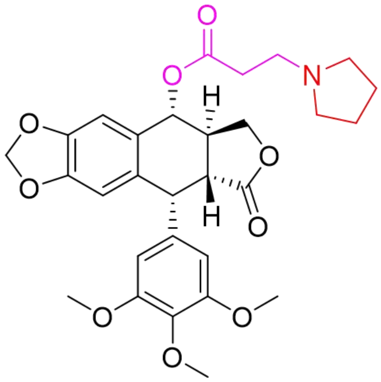

c1(58%)

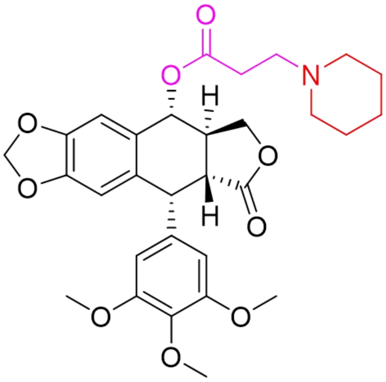

c2(61%)

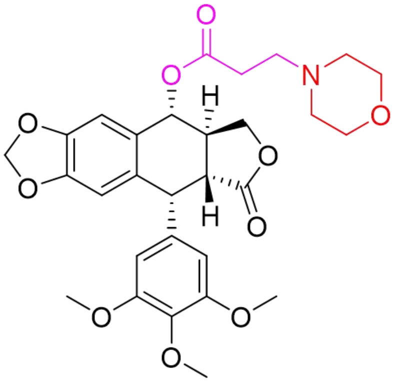

c3(48%)

2.1 Synthesis of compounds a1-a3

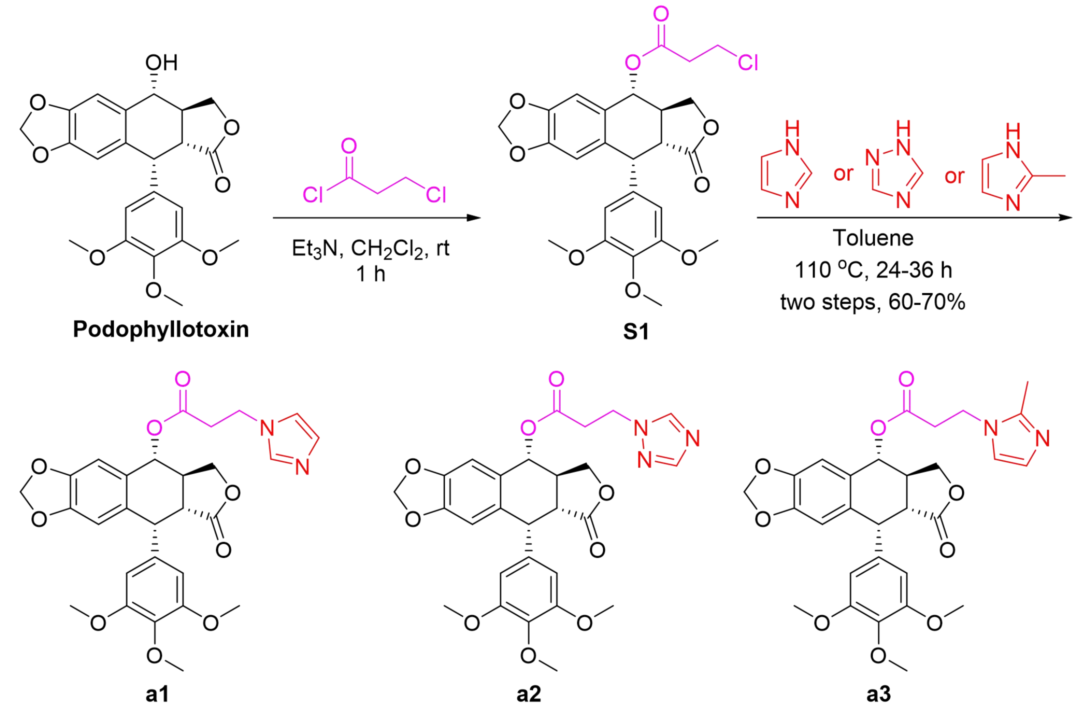

To a solution of podophyllotoxin (1.0 g, 2.41 mmoL) in dichloromethane (10.0 mL) was added 3-chloropropanoyl chloride (0.77 g, 6.03 mmoL) and triethylamine (0.98 g, 9.65 mmoL) at 0 ºC. The resulting mixture was stirred at room temperature for 1 h. After quenching the reaction with water (50.0 mL), the layers were separated. The organic phase was dried over anhydrous Na_2SO_4 and concentrated, and used for the next synthetic step. A mixture of the previous 3-chloropropanoyl chloride **S1** (2.41 mmoL), Na_2CO_3 (0.77g, 7.23mmoL) and various azoles (12.05 mmoL) was stirred in toluene (10.0 mL) at reflux for 24–36 h (monitored by TLC). After cooling to room temperature, the solvent was concentrated, and the residue was diluted with EtOAc (20.0 mL). The organic layer was washed with water and brine , dried over anhydrous Na_2SO_4 and concentrated. The residue was purified by column chromatography (silica gel, petroleum : ethyl acetate = 3:1→dichloromethane : ethyl acetate = 3:1) to afford **a1-a3** in 60-70% yield (two steps) as yellow or white powder.

a1

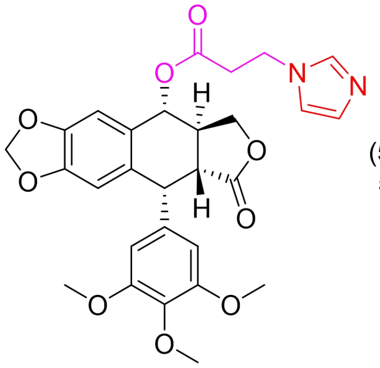

(5*R*,5a*R*,8a*R*,9*R*)-8-oxo-9-(3,4,5-trimethoxyphenyl)-5,5a,6,8,8a,9-hexahydrofuro[3',4':6,7]naphtho[2,3-*d*][1,3]dioxol-5-yl 3-(1*H*-imidazol-1-yl)propanoate

2.1.1 (5*R*,5a*R*,8a*R*,9*R*)-8-oxo-9-(3,4,5-trimethoxyphenyl)-5,5a,6,8,8a,9-hexahydrofuro[3',4':6,7]

naphtho[2,3-*d*][1,3]dioxol-5-yl 3-(1*H*-imidazol-1-yl)propanoate (a1)

Yield 68%. White powder, m.p. 139 - 143 °C. IR ν_{max} (cm^{-1}): 2938, 2251, 1772, 1590, 1505, 1242, 1126, 1037, 912, 732, 663, 529. ^{1}H NMR (400 MHz, Chloroform-*d*) δ 7.39 (s, 1H), 6.98 (s, 1H), 6.81 (s, 1H), 6.65 (s, 1H), 6.57 (s, 1H), 6.32 (s, 2H), 5.97 (d, *J* = 1.2 Hz, 1H), 5.94 (d, *J* = 1.6 Hz, 1H), 5.67 (d, *J* = 4.0 Hz, 1H), 4.41 – 4.35 (m, 2H), 4.17 – 4.06 (m, 3H), 3.78 (d, *J* = 12.8 Hz, 9H), 3.31 (dd, *J* = 9.2, 3.2 Hz, 1H), 2.86 – 2.80 (m, 1H), 2.61 – 2.52 (m, 2H) ppm; ^{13}C NMR (100 MHz, $CDCl_3$) δ^{13}C NMR (100 MHz, $CDCl_3$) δ 177.3, 170.1, 153.2, 148.7, 147.3, 138.9, 137.2, 136.9, 131.4, 129.7, 125.3, 118.7, 110.1, 108.8, 105.5, 101.6, 73.3, 70.5, 60.9, 56.3, 45.1, 44.2, 42.1, 39.3, 35.9 ppm. HRMS (ESI-TOF) *m/z* Calcd for $C_{28}H_{28}N_2O_9$ $[M+H]^+$ 537.1867, found 537.1868.

a2

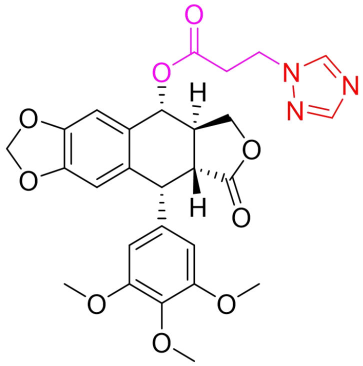

(5*R*,5a*R*,8a*R*,9*R*)-8-oxo-9-(3,4,5-trimethoxyphenyl)-5,5a,6,8,8a,9-hexahydrofuro[3',4':6,7]naphtho[2,3-*d*][1,3]dioxol-5-yl 3-(1*H*-1,2,4-triazol-1-yl)propanoate

2.1.2 (5*R*,5a*R*,8a*R*,9*R*)-8-oxo-9-(3,4,5-trimethoxyphenyl)-5,5a,6,8,8a,9-hexahydrofuro[3',4':6,7] naphtho[2,3-*d*][1,3]dioxol-5-yl 3-(1*H*-1,2,4-triazol-1-yl)propanoate (a2)

Yield 70%. White powder. m.p. 176 - 179 °C. IR ν_{max} (cm^{-1}): 2939, 2250, 1773, 1590, 1505, 1328, 1242, 1126, 1038, 913, 733, 679, 528. ^{1}H NMR (400 MHz, Chloroform-*d*) δ 8.03 (s, 1H), 7.86 (s, 1H), 6.64 (s, 1H), 6.52 (s, 1H), 6.33 (s, 2H), 5.93 (dd, *J* = 10.8, 1.2 Hz, 2H), 5.69 (d, *J* = 4.4 Hz, 1H), 4.38 – 4.30 (m, 4H), 4.17 (dd, *J* = 10.0, 3.2 Hz, 1H), 3.78 (s, 3H), 3.75 (s, 6H), 3.28 (dd, *J* = 9.2, 3.6 Hz, 1H), 2.93 – 2.87 (m, 1H), 2.83 – 2.71 (m, 2H) ppm; ^{13}C NMR (100 MHz, CDCl3) δ 176.2, 169.2, 152.2, 151.1, 147.6, 146.3, 137.8, 130.3, 124.6, 108.9, 107.4, 104.5, 100.5, 72.2, 69.6, 59.8, 55.3, 44.2, 43.5, 43.1, 38.4, 33.0 ppm. HRMS (ESI-TOF) *m/z* Calcd for $C_{27}H_{28}N_3O_9$ $[M+H]^+$ 538.1818, found 538.1820.

a3

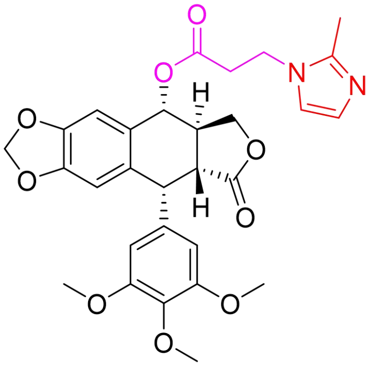

(5*R*,5a*R*,8a*R*,9*R*)-8-oxo-9-(3,4,5-trimethoxyphenyl)-5,5a,6,8,8a,9-hexahydrofuro[3',4':6,7]naphtho[2,3-*d*][1,3]dioxol-5-yl 3-(2-methyl-1*H*-imidazol-1-yl)propanoate

2.1.3 (5*R*,5a*R*,8a*R*,9*R*)-8-oxo-9-(3,4,5-trimethoxyphenyl)-5,5a,6,8,8a,9-hexahydrofuro[3',4':6,7]naphtho[2,3-*d*][1,3]dioxol-5-yl 3-(2-methyl-1*H*-imidazol-1-yl)propanoate (a3)

Yield 60%. White powder. m.p. 148 - 150 °C. IR ν_{max} (cm^{-1}): 1774, 1484, 1239, 1182, 1126, 913, 743. ^{1}H NMR (400 MHz, Chloroform-*d*) δ 6.92 (d, *J* = 1.6 Hz, 1H), 6.87 (d, *J* = 1.6 Hz, 1H), 6.52 (d, *J* = 12.4 Hz, 2H), 6.34 (s, 2H), 5.96 (dd, *J* = 8.0, 1.2 Hz, 2H), 5.87 (d, *J* = 9.2 Hz, 1H), 4.57 (d, *J* = 4.4 Hz, 1H), 4.26 – 4.20 (m, 3H), 4.13 (t, *J* = 9.6 Hz, 1H), 3.78 (s, 3H), 3.73 (s, 6H), 3.57 (q, *J* = 7.2 Hz, 1H), 2.91 – 2.79 (m, 3H), 2.41 (s, 3H) ppm; ^{13}C NMR (100 MHz, CDCl3) δ 172.4, 170.1, 151.7, 147.3, 146.7, 133.7, 131.3, 126.6, 117.7, 108.8, 107.2, 105.8, 100.7, 73.5, 70.0, 59.7, 55.2, 44.5, 42.6, 40.2, 37.5, 34.4 ppm. HRMS (ESI-TOF) *m/z* Calcd for $C_{29}H_{31}N_2O_9$ $[M+H]^+$ 551.2020, found 551.2024.

2.2 Synthesis of compounds a4-a21

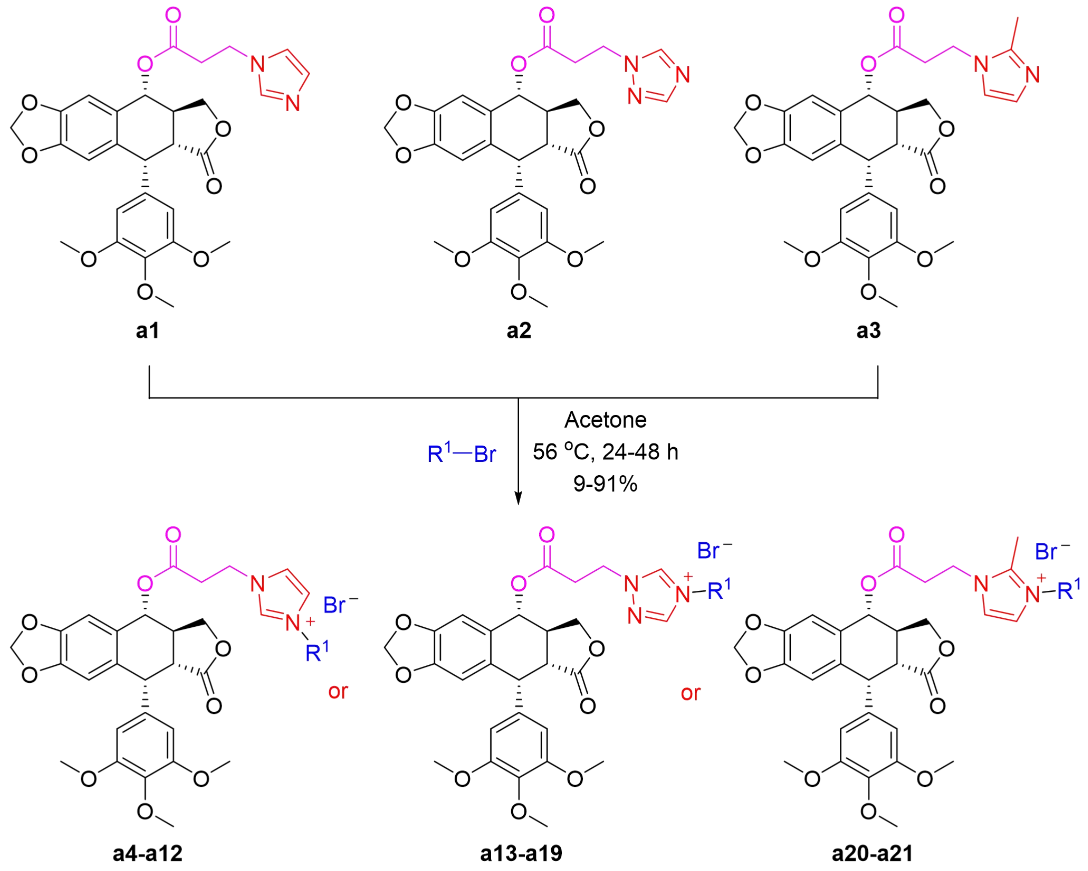

A mixture of compounds a1-a3 (0.1 mmoL) and phenacyl bromides or alkyl halides (1.2 mmoL) was stirred in acetone (10.0 mL) at reflux for 24-48 h. An insoluble substance was formed. After completion of the reaction as indicated by TLC, the precipitate was filtered, and washed with ethyl acetate (3 × 10.0 mL), then dried to afford imidazolium salts a4-a21 in 9–91% yields.

a4

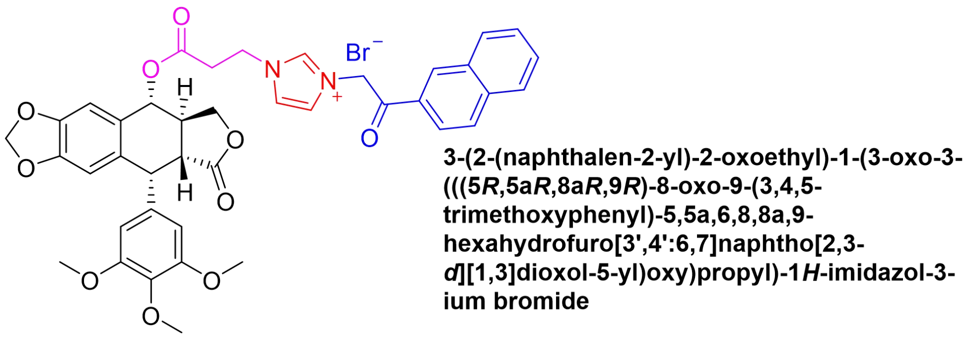

3-(2-(naphthalen-2-yl)-2-oxoethyl)-1-(3-oxo-3-(((5*R*,5a*R*,8a*R*,9*R*)-8-oxo-9-(3,4,5-trimethoxyphenyl)-5,5a,6,8,8a,9-hexahydrofuro[3',4':6,7]naphtho[2,3-*d*][1,3]dioxol-5-yl)oxy)propyl)-1*H*-imidazol-3-ium bromide

2.2.1 3-(2-(naphthalen-2-yl)-2-oxoethyl)-1-(3-oxo-3-(((5*R*,5a*R*,8a*R*,9*R*)-8-oxo-9-(3,4,5-trimethoxyphenyl)-5,5a,6,8,8a,9-hexahydrofuro[3',4':6,7]naphtho[2,3-*d*][1,3]dioxol-5-yl)oxy)propyl)-1*H*-imidazol-3-ium bromide (a4)

Yield 38%. White powder. m.p. 198 - 204 °C. IR ν_{max} (cm^{-1}): 3416, 2257, 2129, 1659, 1026, 826, 764, 630. ^{1}H NMR (400 MHz, DMSO-d_6) δ 9.21 (s, 1H), 8.85 (s, 1H), 8.21 (d, J = 8.0 Hz, 1H), 8.14 (d, J = 8.8 Hz, 1H), 8.09 – 8.03 (m, 2H), 7.88 (s, 1H), 7.79 – 7.68 (m, 3H), 6.87 (s, 1H), 6.57 (s, 2H), 6.52 (s, 1H), 6.23 (s, 2H), 6.01 (s, 2H), 5.76 (d, J = 6.0 Hz, 1H), 4.54 (t, J = 6.4 Hz, 2H), 4.47 – 4.43 (m, 1H), 4.32 – 4.28 (m, 2H), 3.75 (s, 6H), 3.67 – 3.64 (m, 4H), 3.13 – 2.98 (m, 3H) ppm; ^{13}C NMR (100 MHz, DMSO) δ 191.1, 177.1, 170.1, 152.7, 147.3, 146.2, 138.2, 137.7, 136.1, 135.4, 132.3, 131.9, 130.9, 130.4, 129.6, 129.3, 128.7, 127.8, 127.3, 126.6, 124.1, 123.1, 122.1, 108.7, 107.3, 105.7, 101.2, 72.4, 70.1, 59.9, 55.9, 55.4, 44.6, 43.5, 43.2, 33.5 ppm. HRMS (ESI-TOF) *m/z* Calcd for $C_{40}H_{37}N_2O_{10}$ $[M-Br]^+$ 705.2439, found 705.2443.

a5

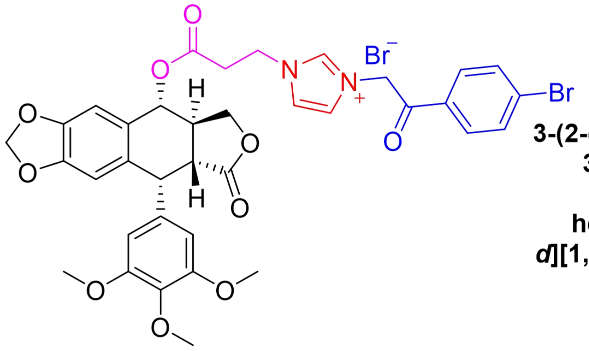

3-(2-(4-bromophenyl)-2-oxoethyl)-1-(3-oxo-3-(((5*R*,5a*R*,8a*R*,9*R*)-8-oxo-9-(3,4,5-trimethoxyphenyl)-5,5a,6,8,8a,9-hexahydrofuro[3',4':6,7]naphtho[2,3-*d*][1,3]dioxol-5-yl)oxy)propyl)-1*H*-imidazol-3-ium bromide

2.2.2 3-(2-(4-bromophenyl)-2-oxoethyl)-1-(3-oxo-3-(((5*R*,5a*R*,8a*R*,9*R*)-8-oxo-9-(3,4,5-trimetho-xyphenyl)-5,5a,6,8,8a,9-hexahydrofuro[3',4':6,7]naphtho[2,3-*d*][1,3]dioxol-5-yl)oxy)propyl)-1*H*-imidazol-3-ium bromide (a5)

Yield 91%. White powder. m.p. 201 - 209 °C. IR ν_{max} (cm^{-1}): 3439, 2253, 2127, 1663, 1485, 1027, 824, 761, 626. ^{1}H NMR (400 MHz, DMSO-d_6) δ 9.15 (s, 1H), 8.00 (d, J = 8.8 Hz, 2H), 7.89 – 7.85 (m, 3H), 7.72 (s, 1H), 6.84 (s, 1H), 6.56 (s, 2H), 6.51 (s, 1H), 6.07 (s, 2H), 6.00 (s, 2H), 5.74 (d, J = 6.0 Hz, 1H), 4.51 (t, J = 6.4 Hz, 2H), 4.46 – 4.42 (m, 1H), 4.30 – 4.27 (m, 2H), 3.74 (s, 6H), 3.64 (t, J = 6.4 Hz, 4H), 3.11 – 2.95 (m, 3H) ppm; ^{13}C NMR (100 MHz, DMSO) δ 190.6, 177.1, 170.1, 152.7, 147.3, 146.2, 138.2, 137.6, 136.1, 132.6, 132.2, 132.1, 130.0, 128.6, 126.6, 124.0, 122.0, 108.7, 107.3, 105.7, 101.2, 72.4, 70.1, 59.9, 55.9, 55.3, 44.5, 43.5, 43.2, 33.5 ppm. HRMS (ESI-TOF) *m/z* Calcd for $C_{36}H_{34}BrN_2O_{10}$ $[M-Br]^+$ 733.1388, found 733.1391.

a6

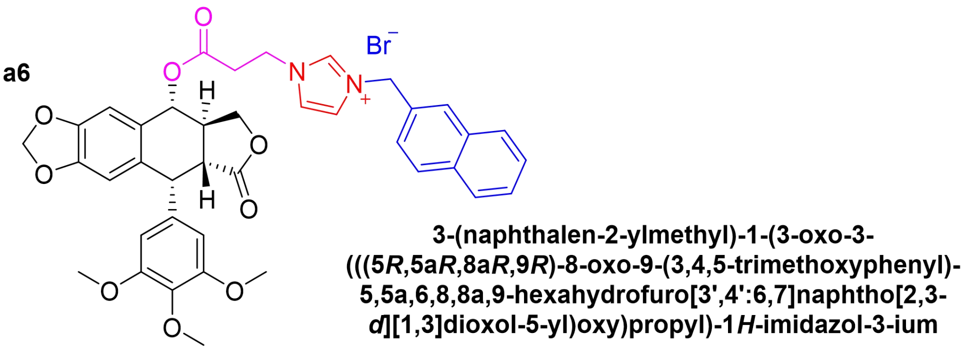

3-(naphthalen-2-ylmethyl)-1-(3-oxo-3-(((5*R*,5a*R*,8a*R*,9*R*)-8-oxo-9-(3,4,5-trimethoxyphenyl)-5,5a,6,8,8a,9-hexahydrofuro[3',4':6,7]naphtho[2,3-*d*][1,3]dioxol-5-yl)oxy)propyl)-1*H*-imidazol-3-ium

2.2.3 3-(naphthalen-2-ylmethyl)-1-(3-oxo-3-(((5*R*,5a*R*,8a*R*,9*R*)-8-oxo-9-(3,4,5-trimethoxyphen-yl)-5,5a,6,8,8a,9-hexahydrofuro[3',4':6,7]naphtho[2,3-*d*][1,3]dioxol-5-yl)oxy)propyl)-1*H*-imida-zol-3-ium (a6)

Yield 58%. White powder. m.p. 201 - 208 °C. IR ν_{max} (cm^{-1}): 3439, 2253, 2127, 1662, 1485, 1027, 824, 761, 626. ^{1}H NMR (400 MHz, DMSO-d_6) δ 9.41 (s, 1H), 7.98 – 7.90 (m, 5H), 7.83 (s, 1H), 7.58 – 7.56 (m, 2H), 7.52 (d, J = 8.4 Hz, 1H), 6.78 (s, 1H), 6.56 (s, 2H), 6.49 (s, 1H), 5.99 (s, 2H), 5.71 (d, J = 6.4 Hz, 1H), 5.63 (s, 2H), 4.44 – 4.37 (m, 3H), 4.29 – 4.25 (m, 2H), 3.73 (s, 6H), 3.64 – 3.60 (m, 4H), 3.05 – 2.95 (m, 3H) ppm; ^{13}C NMR (100 MHz, DMSO) δ 177.7, 170.7, 153.2, 147.8, 146.7, 138.7, 137.3, 136.6, 133.21, 133.19 132.8, 132.7, 129.3, 128.3, 128.2, 128.0, 127.3, 127.2, 126.1, 123.4, 123.1, 109.3, 107.7, 106.3, 101.8, 72.9, 70.5, 60.4, 56.4, 52.6, 45.1, 44.1, 43.8, 33.9 ppm. HRMS (ESI-TOF) *m/z* Calcd for $C_{39}H_{37}N_2O_9$ [M-Br]$^+$ 677.2493, found 677.2494.

a7

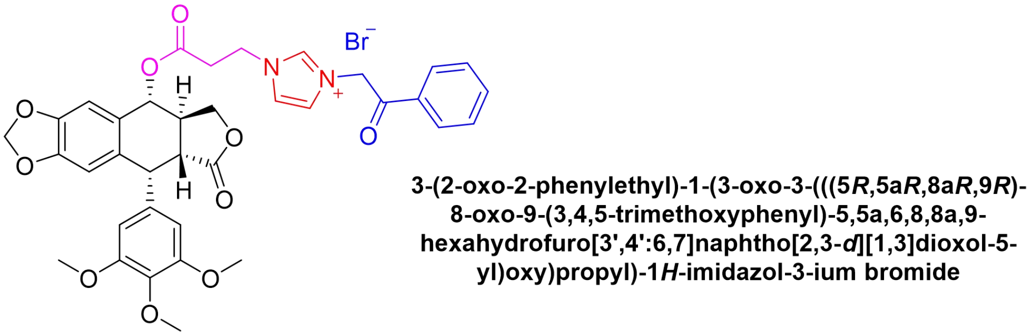

3-(2-oxo-2-phenylethyl)-1-(3-oxo-3-(((5*R*,5a*R*,8a*R*,9*R*)-8-oxo-9-(3,4,5-trimethoxyphenyl)-5,5a,6,8,8a,9-hexahydrofuro[3',4':6,7]naphtho[2,3-*d*][1,3]dioxol-5-yl)oxy)propyl)-1*H*-imidazol-3-ium bromide

2.2.4 3-(2-oxo-2-phenylethyl)-1-(3-oxo-3-(((5*R*,5a*R*,8a*R*,9*R*)-8-oxo-9-(3,4,5-trimethoxyphenyl)-5,5a,6,8,8a,9-hexahydrofuro[3',4':6,7]naphtho[2,3-*d*][1,3]dioxol-5-yl)oxy)propyl)-1*H*-imidazol-3-ium bromide (a7)

Yield 44%. White powder. m.p. 204 - 210 °C. IR ν_{max} (cm^{-1}): 3439, 2253, 2127, 1662, 1234, 1027, 824, 761, 626. ^{1}H NMR (400 MHz, DMSO-d_6) δ 9.16 (s, 1H), 8.07 (d, J = 7.2 Hz, 2H), 7.85 (s, 1H), 7.80 – 7.74 (m, 2H), 7.65 (t, J = 8.0 Hz, 2H), 6.85 (s, 1H), 6.56 (s, 2H), 6.51 (s, 1H), 6.09 (s, 2H), 6.00 (s, 2H), 5.75 (d, J = 2.0 Hz, 1H), 4.52 (t, J = 6.4 Hz, 2H), 4.46 – 4.42 (m, 1H), 4.31 – 4.27 (m, 2H), 3.74 (s, 6H), 3.64 – 3.63 (m, 4H), 3.10 – 2.96 (m, 3H) ppm; ^{13}C NMR (100 MHz, DMSO) δ 191.1, 177.1, 170.1, 152.7, 147.3, 146.2, 138.2, 137.7, 136.0, 134.5, 133.6, 132.2, 129.0, 128.1, 126.6, 124.0, 122.0, 108.7, 107.3, 105.7, 101.2, 72.4, 70.0, 59.9, 55.9, 55.3, 44.5, 43.5, 43.2, 33.5 ppm. HRMS (ESI-TOF) *m/z* Calcd for $C_{36}H_{35}N_2O_{10}$ [M-Br]$^+$ 655.2282, found 655.2286.

a8

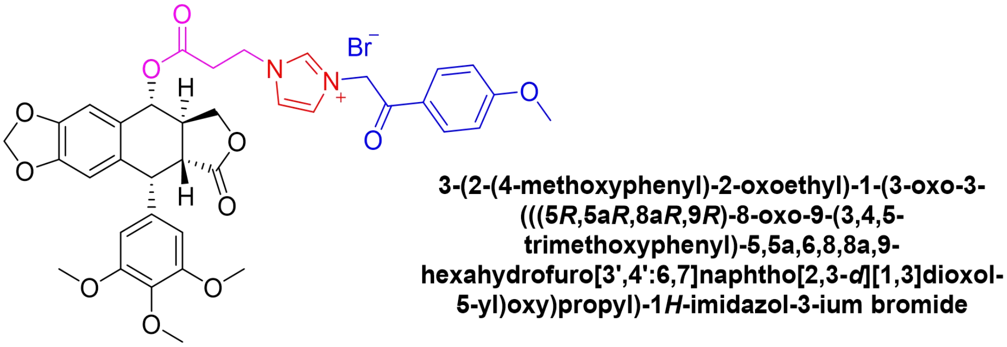

3-(2-(4-methoxyphenyl)-2-oxoethyl)-1-(3-oxo-3-(((5*R*,5a*R*,8a*R*,9*R*)-8-oxo-9-(3,4,5-trimethoxyphenyl)-5,5a,6,8,8a,9-hexahydrofuro[3',4':6,7]naphtho[2,3-*d*][1,3]dioxol-5-yl)oxy)propyl)-1*H*-imidazol-3-ium bromide

2.2.5 3-(2-(4-methoxyphenyl)-2-oxoethyl)-1-(3-oxo-3-(((5*R*,5a*R*,8a*R*,9*R*)-8-oxo-9-(3,4,5-trimet-h

oxyphenyl)-5,5a,6,8,8a,9-hexahydrofuro[3',4':6,7]naphtho[2,3-*d*][1,3]dioxol-5-yl)oxy)propyl)-1*H*-imidazol-3-ium bromide (a8)

Yield 53%. White powder. m.p. 201 - 202 °C. IR v_{max} (cm^{-1}): 3439, 2253, 2126, 1663, 1514, 1127, 1004, 823, 761, 626. ^{1}H NMR (400 MHz, DMSO-d_6) δ 9.19 (s, 1H), 8.05 (d, *J* = 8.4 Hz, 2H), 7.85 (s, 1H), 7.75 (s, 1H), 7.16 (d, *J* = 8.4 Hz, 2H), 6.85 (s, 1H), 6.57 (s, 2H), 6.52 (s, 1H), 6.03 (d, *J* = 18.8 Hz, 4H), 5.75 (d, *J* = 6.0 Hz, 1H), 4.51 (t, *J* = 6.4 Hz, 2H), 4.47 – 4.43 (m, 1H), 4.31 – 4.27 (m, 2H), 3.89 (s, 3H), 3.74 (s, 6H), 3.65 (s, 4H), 3.12 – 2.96 (m, 3H) ppm; ^{13}C NMR (100 MHz, DMSO) δ 190.0, 177.7, 170.6, 164.6, 153.2, 147.9, 146.8, 138.8, 138.2, 136.6, 132.8, 131.1, 127.2, 127.0, 124.6, 122.5, 114.8, 109.3, 107.9, 106.3, 101.8, 72.9, 70.6, 60.5, 56.5, 56.3, 55.6, 45.1, 44.1, 43.8, 34.1 ppm. HRMS (ESI-TOF) *m/z* Calcd for $C_{37}H_{37}N_2O_{11}$ [M-Br]$^+$ 685.2394, found 685.2392.

a9

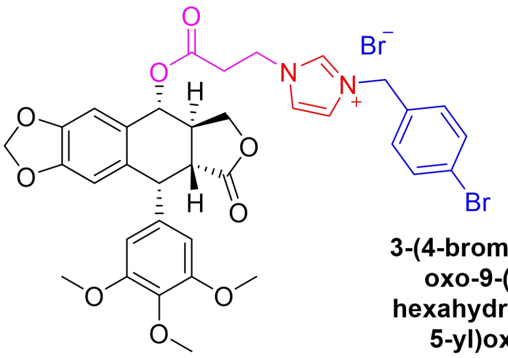

2.2.6 3-(4-bromobenzyl)-1-(3-oxo-3-(((5*R*,5a*R*,8a*R*,9*R*)-8-oxo-9-(3,4,5-trimethoxyphenyl)-5,5a,6,8,8a,9-hexahydrofuro[3',4':6,7]naphtho[2,3-*d*][1,3]dioxol-5-yl)oxy)propyl)-1*H*-imidazol-3-ium-bromide (a9)

Yield 82%. White powder. m.p. 197 - 199 °C. IR v_{max} (cm^{-1}): 3439, 2252, 2126, 1663, 1485, 1125, 1027, 823, 761, 626. ^{1}H NMR (400 MHz, DMSO-d_6) δ 9.34 (s, 1H), 7.81 (d, *J* = 11.6 Hz, 2H), 7.62 (d, *J* = 8.4 Hz, 2H), 7.38 (d, *J* = 8.0 Hz, 2H), 6.77 (s, 1H), 6.55 (s, 2H), 6.49 (s, 1H), 6.00 (s, 2H), 5.71 (d, *J* = 6.0 Hz, 1H), 5.44 (s, 2H), 4.43 – 4.39 (m, 3H), 4.30 – 4.25 (m, 2H), 3.73 (s, 6H), 3.63 (s, 4H), 3.06 – 2.94 (m, 3H) ppm; ^{13}C NMR (100 MHz, DMSO) δ 177.1, 170.1, 152.7, 147.2, 146.1, 138.1, 136.7, 136.1, 134.0, 132.2, 131.8, 130.4, 126.7, 122.8, 122.4, 122.0, 108.7, 107.1, 105.8, 101.2, 72.3, 69.9, 59.9, 55.9, 51.1, 44.5, 43.5, 43.2, 33.3 ppm. HRMS (ESI-TOF) *m/z* Calcd for $C_{35}H_{34}BrN_2O_9$ [M-Br]$^+$ 705.1442, found 705.1442.

a10

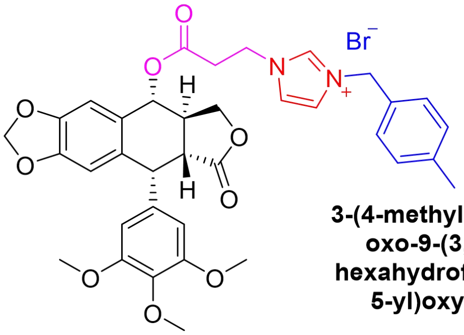

2.2.7 3-(4-methylbenzyl)-1-(3-oxo-3-(((5*R*,5a*R*,8a*R*,9*R*)-8-oxo-9-(3,4,5-trimethoxyphenyl)-5,5a,6,

8,8a,9-hexahydrofuro[3',4':6,7]naphtho[2,3-*d*][1,3]dioxol-5-yl)oxy)propyl)-1*H*-imidazol-3-ium bromide (a10)

Yield 46%. White powder. m.p. 191 - 193 °C. IR ν_{max} (cm^{-1}): 3420, 2254, 2128, 1660, 1506, 1027, 825, 763, 629. ^{1}H NMR (400 MHz, DMSO-d_6) δ 9.37 (s, 1H), 7.81 (d, *J* = 11.2 Hz, 2H), 7.32 (d, *J* = 8.0 Hz, 2H), 7.21 (d, *J* = 7.6 Hz, 2H), 6.78 (s, 1H), 6.56 (s, 2H), 6.50 (s, 1H), 6.00 (s, 2H), 5.71 (d, *J* = 6.0 Hz, 1H), 5.41 (s, 2H), 4.43 – 4.40 (m, 3H), 4.28 – 4.25 (m, 2H), 3.73 (s, 6H), 3.63 (s, 4H), 3.06 – 2.96 (m, 3H),2.29 (s, 3H) ppm; ^{13}C NMR (100 MHz, DMSO) δ 177.7, 170.6, 153.2, 147.9, 146.7, 138.75, 138.7, 137.0, 136.7, 132.8, 132.2, 130.0, 128.8, 127.2, 123.3, 122.9, 109.3, 107.7, 106.4, 101.8, 72.9, 70.5, 60.4, 56.5, 52.2, 45.0, 44.1, 43.8, 33.9, 21.2 ppm. HRMS (ESI-TOF) *m/z* Calcd for $C_{36}H_{37}N_2O_9$ [M-Br]$^+$ 642.2498, found 642.2494.

a11

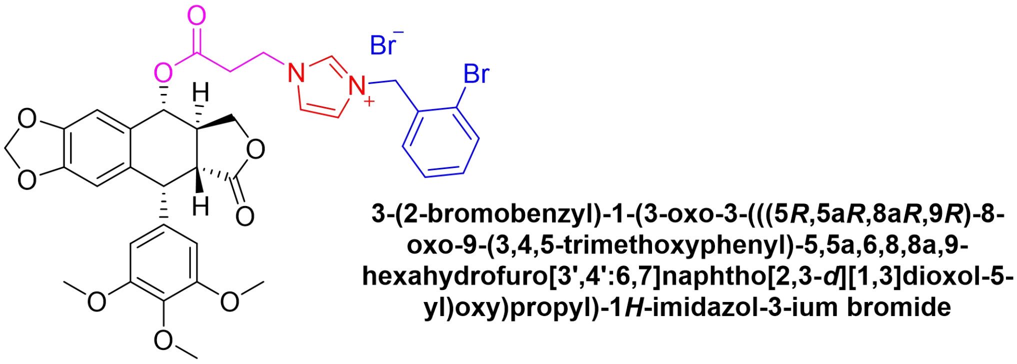

3-(2-bromobenzyl)-1-(3-oxo-3-(((5*R*,5a*R*,8a*R*,9*R*)-8-oxo-9-(3,4,5-trimethoxyphenyl)-5,5a,6,8,8a,9-hexahydrofuro[3',4':6,7]naphtho[2,3-*d*][1,3]dioxol-5-yl)oxy)propyl)-1*H*-imidazol-3-ium bromide

2.2.8 3-(2-bromobenzyl)-1-(3-oxo-3-(((5*R*,5a*R*,8a*R*,9*R*)-8-oxo-9-(3,4,5-trimethoxyphenyl)-5,5a,6,8,8a,9-hexahydrofuro[3',4':6,7]naphtho[2,3-*d*][1,3]dioxol-5-yl)oxy)propyl)-1*H*-imidazol-3-ium bromide (a11)

Yield 81%. White powder. m.p. 191 - 192 °C. IR ν_{max} (cm^{-1}): 3435, 2253, 2127, 1663, 1485, 1027, 824, 762, 626. ^{1}H NMR (400 MHz, DMSO-d_6) δ 9.33 (s, 1H), 7.86 (s, 1H), 7.80 (s, 1H), 7.74 (d, *J* = 7.6 Hz, 1H), 7.49 – 7.45 (m, 1H), 7.41 – 7.34 (m, 2H), 6.79 (s, 1H), 6.56 (s, 2H), 6.49 (s, 1H), 6.00 (s, 2H), 5.72 (d, *J* = 6.4 Hz, 1H), 5.55 (s, 2H), 4.46 – 4.40 (m, 3H), 4.30 – 4.26 (m, 2H), 3.73 (s, 6H), 3.66 – 3.62 (m, 4H), 3.08 – 2.96 (m, 3H) ppm; ^{13}C NMR (100 MHz, DMSO) δ 177.7, 170.6, 153.2, 147.8, 146.7, 138.7, 137.7, 136.6, 134.0, 133.6, 132.8, 131.5, 131.1, 129.0, 127.2, 123.5, 123.4, 123.3, 109.3, 107.7, 106.3, 101.8, 72.9, 70.6, 60.4, 56.4, 52.7, 45.1, 44.1, 43.8, 33.9 ppm. HRMS (ESI-TOF) *m/z* Calcd for $C_{35}H_{34}BrN_2O_9$ [M-Br]$^+$ 705.1444, found 705.1442.

a12

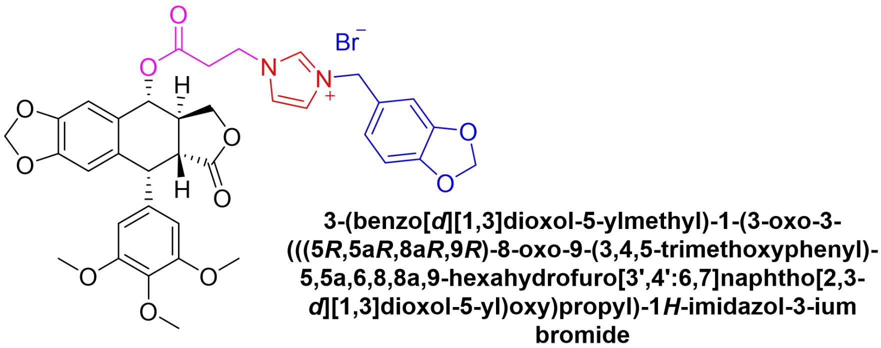

3-(benzo[*d*][1,3]dioxol-5-ylmethyl)-1-(3-oxo-3-(((5*R*,5a*R*,8a*R*,9*R*)-8-oxo-9-(3,4,5-trimethoxyphenyl)-5,5a,6,8,8a,9-hexahydrofuro[3',4':6,7]naphtho[2,3-*d*][1,3]dioxol-5-yl)oxy)propyl)-1*H*-imidazol-3-ium bromide

2.2.9 3-(benzo[d][1,3]dioxol-5-ylmethyl)-1-(3-oxo-3-(((5*R*,5a*R*,8a*R*,9*R*)-8-oxo-9-(3,4,5-trimethoxyphenyl)-5,5a,6,8,8a,9-hexahydrofuro[3',4':6,7]naphtho[2,3-*d*][1,3]dioxol-5-yl)oxy)propyl)-1*H*-imidazol-3-ium bromide (a12)

Yield 78%. White powder. m.p. 197 - 200 °C. IR ν_{max} (cm^{-1}): 3442, 2252, 2126, 1662, 1486, 1250, 1027, 823, 761, 625. ^1H NMR (400 MHz, DMSO-d_6) δ 9.28 (s, 1H), 7.76 - 7.75 (m, 1H), 7.71 - 7.70 (m, 1H), 7.01 (d, J = 1.6 Hz, 1H), 6.93 – 6.86 (m, 2H), 6.70 (s, 1H), 6.48 (s, 2H), 6.42 (s, 1H), 5.96 (d, J = 1.6 Hz, 2H), 5.93 (s, 2H), 5.63 (d, J = 6.0 Hz, 1H), 5.27 (s, 2H), 4.36 – 4.31 (m, 3H), 4.21 – 4.18 (m, 2H), 3.66 (s, 6H), 3.58 - 3.54 (m, 4H), 2.98 – 2.88 (m, 3H) ppm; ^{13}C NMR (100 MHz, DMSO) δ 177.7, 170.6, 153.2, 148.1, 147.9, 146.7, 138.7, 136.9, 136.7, 132.8, 128.7, 127.2, 123.2, 123.0, 122.8, 109.4, 109.3, 109.0, 107.7, 106.4, 101.9, 101.8, 72.9, 70.5, 60.4, 56.5, 52.2, 45.0, 44.1, 43.8, 33.9 ppm. HRMS (ESI-TOF) *m/z* Calcd for $C_{36}H_{35}N_2O_{11}$ [M-Br]$^+$ 671.2235, found 671.2235.

a13

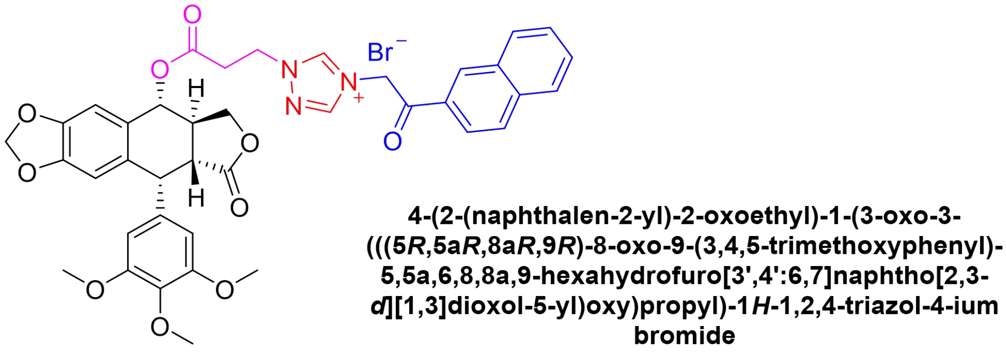

4-(2-(naphthalen-2-yl)-2-oxoethyl)-1-(3-oxo-3-(((5*R*,5a*R*,8a*R*,9*R*)-8-oxo-9-(3,4,5-trimethoxyphenyl)-5,5a,6,8,8a,9-hexahydrofuro[3',4':6,7]naphtho[2,3-*d*][1,3]dioxol-5-yl)oxy)propyl)-1*H*-1,2,4-triazol-4-ium bromide

2.2.10 4-(2-(naphthalen-2-yl)-2-oxoethyl)-1-(3-oxo-3-(((5*R*,5a*R*,8a*R*,9*R*)-8-oxo-9-(3,4,5-trimethoxyphenyl)-5,5a,6,8,8a,9-hexahydrofuro[3',4':6,7]naphtho[2,3-*d*][1,3]dioxol-5-yl)oxy)propyl)-1*H*-1,2,4-triazol-4-ium bromide (a13)

Yield 30%. White powder. m.p. 183 - 185 °C. IR ν_{max} (cm^{-1}): 3424, 2255, 2128, 1660, 1026, 825, 763, 629. ^1H NMR (400 MHz, DMSO-d_6) δ 10.12 (s, 1H), 9.16 (s, 1H), 8.79 (s, 1H), 8.15 (d, *J* = 8.4 Hz, 1H), 8.08 (d, *J* = 8.8 Hz, 1H), 8.02 – 7.97 (m, 2H), 7.71 – 7.62 (m, 2H), 6.84 (s, 1H), 6.50 (s, 2H), 6.45 (s, 1H), 6.24 (s, 2H), 5.93 (s, 2H), 5.69 (d, *J* = 6.4 Hz, 1H), 4.72 – 4.69 (m, 2H), 4.41 - 4.37 (m, 1H), 4.28 – 4.21 (m, 2H), 3.67 (s, 6H), 3.65 - 3.55 (m, 4H), 3.08 – 2.95 (m, 3H) ppm; ^{13}C NMR (100 MHz, DMSO) δ 190.7, 177.7, 170.5, 153.3, 147.9, 146.8, 146.3, 144.5, 138.8, 136.7, 136.1, 132.8, 132.5, 131.2, 130.2, 130.0, 129.4, 128.4, 128.0, 127.1, 123.6, 109.3, 108.0, 106.4, 101.8, 73.0, 70.7, 60.5, 56.5, 54.5, 47.9, 44.2, 43.8, 32.7 ppm. HRMS (ESI-TOF) *m/z* Calcd for $C_{39}H_{36}BrN_3O_{10}$ [M-Br]$^+$ 706.2396, found 706.2395.

a14

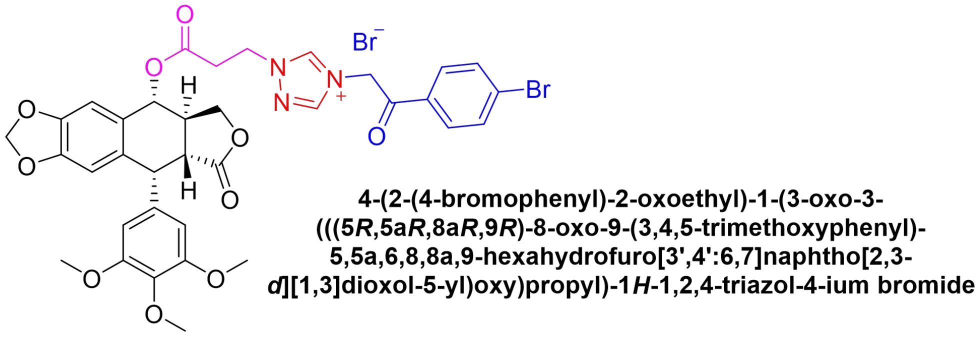

4-(2-(4-bromophenyl)-2-oxoethyl)-1-(3-oxo-3-(((5*R*,5a*R*,8a*R*,9*R*)-8-oxo-9-(3,4,5-trimethoxyphenyl)-5,5a,6,8,8a,9-hexahydrofuro[3',4':6,7]naphtho[2,3-*d*][1,3]dioxol-5-yl)oxy)propyl)-1*H*-1,2,4-triazol-4-ium bromide

2.2.11 4-(2-(4-bromophenyl)-2-oxoethyl)-1-(3-oxo-3-(((5*R*,5a*R*,8a*R*,9*R*)-8-oxo-9-(3,4,5-trimethox

yphenyl)-5,5a,6,8,8a,9-hexahydrofuro[3',4':6,7]naphtho[2,3-*d*][1,3]dioxol-5-yl)oxy)propyl)-1*H*-1,2,4-triazol-4-ium bromide (a14)

Yield 34%. White powder. m.p. 181 - 184 °C. IR ν_{max} (cm^{-1}): 3431, 2254, 2127, 1660, 1486, 1027, 824, 762, 627. ^{1}H NMR (400 MHz, DMSO-d_6) δ 10.07 (s, 1H), 9.10 (s, 1H), 7.94 (d, J = 8.8 Hz, 2H), 7.82 (d, J = 8.4 Hz, 2H), 6.82 (s, 1H), 6.49 (s, 2H), 6.45 (s, 1H), 6.09 (s, 2H), 5.93 (s, 2H), 5.67 (d, J = 6.0 Hz, 1H), 4.70 – 4.67 (m, 2H), 4.39 – 4.35 (m, 1H), 4.26 – 4.20 (m, 2H), 3.67 (s, 6H), 3.58 - 3.54 (s, 4H), 3.05 – 2.91 (m, 3H) ppm; ^{13}C NMR (100 MHz, DMSO) δ 190.2, 177.7, 170.5, 153.2, 147.9, 146.8, 146.2, 144.4, 138.8, 136.7, 132.9, 132.8, 130.7, 129.4, 127.1, 109.3, 108.0, 106.4, 101.7, 73.0, 70.7, 60.5, 56.5, 54.4, 47.9, 44.2, 43.8, 32.7 ppm. HRMS (ESI-TOF) *m/z* Calcd for $C_{35}H_{33}BrN_3O_{10}$ [M-Br]$^+$ 734.1343, found 734.1344.

a15

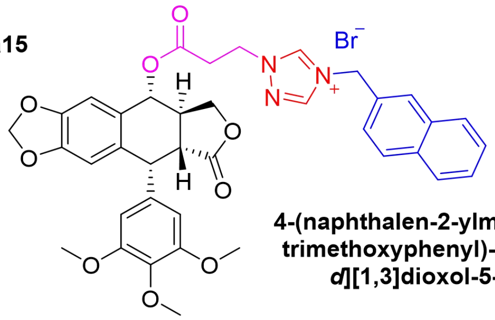

4-(naphthalen-2-ylmethyl)-1-(3-oxo-3-(((5*R*,5a*R*,8a*R*,9*R*)-8-oxo-9-(3,4,5-trimethoxyphenyl)-5,5a,6,8,8a,9-hexahydrofuro[3',4':6,7]naphtho[2,3-*d*][1,3]dioxol-5-yl)oxy)propyl)-1*H*-1,2,4-triazol-4-ium bromide

2.2.2.12 4-(naphthalen-2-ylmethyl)-1-(3-oxo-3-(((5*R*,5a*R*,8a*R*,9*R*)-8-oxo-9-(3,4,5-trimethoxyphe-nyl)-5,5a,6,8,8a,9-hexahydrofuro[3',4':6,7]naphtho[2,3-*d*][1,3]dioxol-5-yl)oxy)propyl)-1*H*-1,2,4-triazol-4-ium bromide (a15)

Yield 63%. White powder. m.p. 201 - 210 °C. IR ν_{max} (cm^{-1}): 3424, 2255, 2128, 1659, 1026, 825, 763, 629. ^{1}H NMR (400 MHz, DMSO-d_6) δ 10.28 (s, 1H), 9.36 (s, 1H), 7.96 – 7.92 (m, 2H), 7.90 – 7.85 (m, 2H), 7.54 – 7.49 (m, 3H), 6.78 (s, 1H), 6.47 (s, 2H), 6.43 (s, 1H), 5.92 (s, 2H), 5.67 (s, 2H), 5.64 (d, J = 6.4 Hz, 1H), 4.59 - 4.51 (m, 2H), 4.35 – 4.31 (m, 1H), 4.23 (dd, J = 9.6, 2.0 Hz, 1H), 4.19 (d, J = 4.8 Hz, 1H), 3.65 (s, 6H), 3.56 – 3.52 (m, 4H), 2.97 – 2.90 (m, 3H) ppm; ^{13}C NMR (100 MHz, DMSO) δ 177.7, 170.5, 153.2, 147.9, 146.8, 145.2, 143.7, 138.8, 136.7, 133.3, 133.2, 132.7, 131.5, 129.4, 128.5, 128.4, 128.2, 127.4, 127.3, 127.2, 126.3, 109.3, 107.9, 106.4, 101.7, 73.0, 70.7, 60.5, 56.5, 51.2, 47.8, 44.2, 43.8, 32.6 ppm. HRMS (ESI-TOF) *m/z* Calcd for $C_{38}H_{36}N_3O_9$ [M-Br]$^+$ 678.2446, found 678.2446.

a16

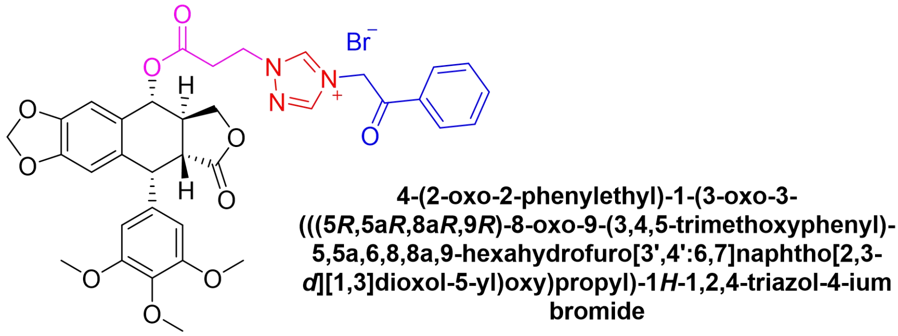

4-(2-oxo-2-phenylethyl)-1-(3-oxo-3-(((5*R*,5a*R*,8a*R*,9*R*)-8-oxo-9-(3,4,5-trimethoxyphenyl)-5,5a,6,8,8a,9-hexahydrofuro[3',4':6,7]naphtho[2,3-*d*][1,3]dioxol-5-yl)oxy)propyl)-1*H*-1,2,4-triazol-4-ium bromide

2.2.13 4-(2-oxo-2-phenylethyl)-1-(3-oxo-3-(((5*R*,5a*R*,8a*R*,9*R*)-8-oxo-9-(3,4,5-trimethoxyphenyl)-5,5a,6,8,8a,9-hexahydrofuro[3',4':6,7]naphtho[2,3-*d*][1,3]dioxol-5-yl)oxy)propyl)-1*H*-1,2,4-triaz

ol-4-ium bromide (a16)

Yield 37%. White powder. m.p. 198 - 200 °C. IR ν_{max} (cm^{-1}): 3424, 2255, 2128, 1659, 1026, 825, 763, 629. ^{1}H NMR (400 MHz, DMSO-d_6) δ 10.07 (s, 1H), 9.11 (s, 1H), 8.01 (d, J = 8.0 Hz, 2H), 7.72 (t, J = 7.2 Hz, 1H), 7.59 (t, J = 7.6 Hz, 2H), 6.82 (s, 1H), 6.49 (s, 2H), 6.45 (s, 1H), 6.10 (s, 2H), 5.93 (s, 2H), 5.68 (d, J = 6.4 Hz, 1H), 4.68 (s, 2H), 4.39 – 4.35 (m, 1H), 4.26 – 4.20 (m, 2H), 3.67 (s, 6H), 3.60 – 3.53 (m, 4H), 3.06 – 2.93 (m, 3H) ppm; ^{13}C NMR (100 MHz, DMSO) δ 190.8, 177.7, 170.5, 153.2, 147.9, 146.8, 146.2, 144.4, 138.8, 136.7, 135.2, 133.9, 132.8, 129.7, 128.7, 127.1, 109.3, 108.0, 106.4, 101.7, 73.0, 70.7, 60.5, 56.5, 54.4, 47.9, 44.2, 43.8, 32.7 ppm. HRMS (ESI-TOF) m/z Calcd for $C_{35}H_{34}N_3O_{10}$ $[M-Br]^+$ 656.2237, found 656.2239.

a17

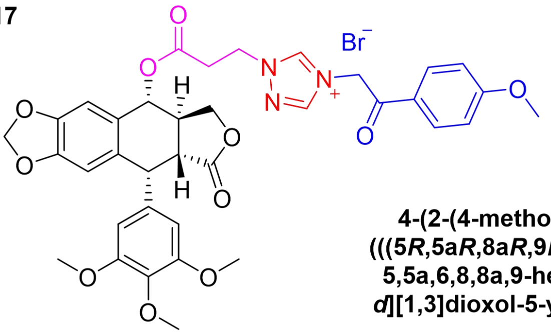

4-(2-(4-methoxyphenyl)-2-oxoethyl)-1-(3-oxo-3-(((5*R*,5a*R*,8a*R*,9*R*)-8-oxo-9-(3,4,5-trimethoxyphenyl)-5,5a,6,8,8a,9-hexahydrofuro[3',4':6,7]naphtho[2,3-*d*][1,3]dioxol-5-yl)oxy)propyl)-1*H*-1,2,4-triazol-4-ium bromide

2.2.14 4-(2-(4-methoxyphenyl)-2-oxoethyl)-1-(3-oxo-3-(((5*R*,5a*R*,8a*R*,9*R*)-8-oxo-9-(3,4,5-trimethoxyphenyl)-5,5a,6,8,8a,9-hexahydrofuro[3',4':6,7]naphtho[2,3-*d*][1,3]dioxol-5-yl)oxy)propyl)-1*H*-1,2,4-triazol-4-ium bromide (a17)

Yield 80%. White powder. m.p. 201 - 202 °C. IR ν_{max} (cm^{-1}): 3435, 2253, 2126, 1662, 1485, 1243, 1027, 823, 761, 626. ^{1}H NMR (400 MHz, DMSO-d_6) δ 10.12 (s, 1H), 9.14 (s, 1H), 7.99 (d, J = 8.8 Hz, 2H), 7.10 (d, J = 8.8 Hz, 2H), 6.83 (s, 1H), 6.49 (s, 2H), 6.45 (s, 1H), 6.08 (s, 2H), 5.93 (s, 2H), 5.68 (d, J = 6.0 Hz, 1H), 4.73 - 4.64 (m, 2H), 4.40 – 3.36 (m, 1H), 4.26 – 4.20 (m, 2H), 3.83 (s, 3H), 3.67 (s, 6H), 3.58 - 3.55 (m, 4H), 3.04 – 2.93 (m, 3H) ppm; ^{13}C NMR (100 MHz, DMSO) δ 189.0, 177.7, 170.5, 164.8, 153.2, 147.9, 146.8, 146.2, 144.4, 138.8, 136.7, 132.8, 131.2, 127.1, 126.7, 114.9, 109.3, 108.0, 106.4, 101.8, 73.0, 70.7, 60.5, 56.5, 56.3, 54.1, 47.9, 44.2, 43.8, 32.7 ppm. HRMS (ESI-TOF) m/z Calcd for $C_{36}H_{36}N_3O_{11}$ $[M-Br]^+$ 686.2346, found 686.2344.

a18

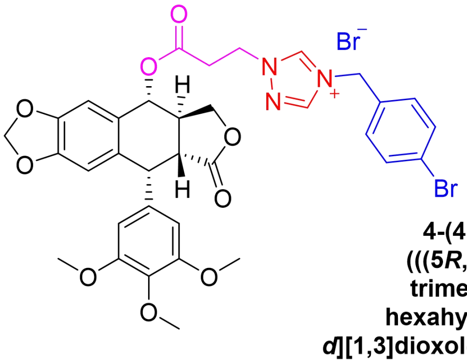

4-(4-bromobenzyl)-1-(3-oxo-3-(((5*R*,5a*R*,8a*R*,9*R*)-8-oxo-9-(3,4,5-trimethoxyphenyl)-5,5a,6,8,8a,9-hexahydrofuro[3',4':6,7]naphtho[2,3-*d*][1,3]dioxol-5-yl)oxy)propyl)-1*H*-1,2,4-triazol-4-ium bromide

2.2.15 4-(4-bromobenzyl)-1-(3-oxo-3-(((5*R*,5a*R*,8a*R*,9*R*)-8-oxo-9-(3,4,5-trimethoxyphenyl)-5,5a,6,8,8a,9-hexahydrofuro[3',4':6,7]naphtho[2,3-*d*][1,3]dioxol-5-yl)oxy)propyl)-1*H*-1,2,4-triazol-4-ium bromide (a18)

Yield 71%. White powder. m.p. 195 - 196 °C. IR ν_{max} (cm^{-1}): 3431, 2253, 2127, 1660, 1027, 824, 762, 627. ^{1}H NMR (400 MHz, DMSO-d_6) δ 10.31 (s, 1H), 9.38 (s, 1H), 7.66 (d, *J* = 8.4 Hz, 2H), 7.47 (d, *J* = 8.4 Hz, 2H), 6.84 (s, 1H), 6.55 (s, 2H), 6.49 (s, 1H), 6.00 (s, 2H), 5.72 (d, *J* = 6.0 Hz, 1H), 5.56 (s, 2H), 4.62 (t, *J* = 6.8 Hz, 2H), 4.44 – 4.40 (m, 1H), 4.31 (dd, *J* = 9.2, 2.0 Hz, 1H), 4.26 (d, *J* = 4.8 Hz, 1H), 3.73 (s, 6H), 3.64 - 3.60 (m, 4H), 3.06 – 2.96 (m, 3H) ppm; ^{13}C NMR (100 MHz, DMSO) δ 177.7, 170.5, 153.2, 147.8, 146.7, 145.1, 143.6, 138.8, 136.6, 133.4, 132.7, 132.4, 131.5, 127.2, 123.0, 109.2, 107.8, 106.3, 101.7, 73.0, 70.6, 60.4, 56.4, 50.2, 47.8, 44.2, 43.7, 32.6 ppm; HRMS (ESI-TOF) *m/z* Calcd for $C_{34}H_{33}BrN_3O_9$[M-Br]$^+$ 706.1391, found 706.1395.

a19

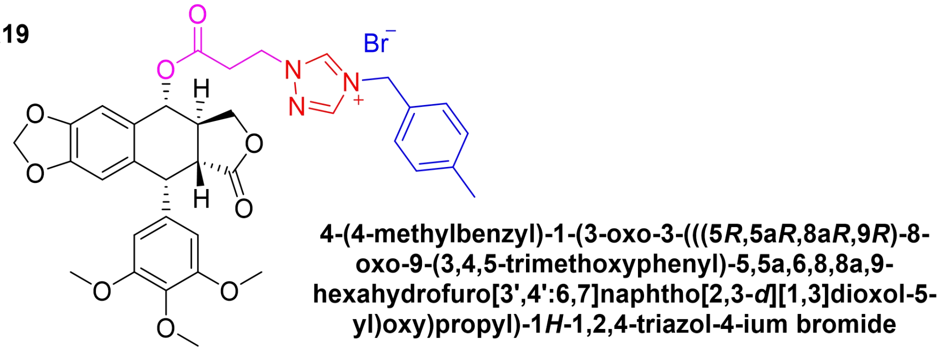

4-(4-methylbenzyl)-1-(3-oxo-3-(((5*R*,5a*R*,8a*R*,9*R*)-8-oxo-9-(3,4,5-trimethoxyphenyl)-5,5a,6,8,8a,9-hexahydrofuro[3',4':6,7]naphtho[2,3-*d*][1,3]dioxol-5-yl)oxy)propyl)-1*H*-1,2,4-triazol-4-ium bromide

2.2.16 4-(4-methylbenzyl)-1-(3-oxo-3-(((5*R*,5a*R*,8a*R*,9*R*)-8-oxo-9-(3,4,5-trimethoxyphenyl)-5,5a,6,8,8a,9-hexahydrofuro[3',4':6,7]naphtho[2,3-*d*][1,3]dioxol-5-yl)oxy)propyl)-1*H*-1,2,4-triazol-4-ium bromide (a19)

Yield 9%. White powder. m.p. 198 - 201 °C. IR ν_{max} (cm^{-1}): 3424, 2255, 2128, 1660, 1027, 825, 763, 629. ^{1}H NMR (400 MHz, DMSO-d_6) δ 10.19 (s, 1H), 9.27 (s, 1H), 7.30 (d, *J* = 8.0 Hz, 2H), 7.18 (d, *J* = 7.6 Hz, 2H), 6.77 (s, 1H), 6.47 (s, 2H), 6.43 (s, 1H), 5.93 (s, 2H), 5.63 (d, *J* = 6.0 Hz, 1H), 5.43 (s, 2H), 4.54 (t, *J* = 6.8 Hz, 2H), 4.33 (dd, *J* = 9.6, 6.8 Hz,1H), 4.22 (dd, *J* = 9.2, 2.0 Hz, 1H), 4.19 (d, *J* = 4.8 Hz, 1H), 3.65 (s, 6H), 3.57 – 3.51 (m, 4H), 2.98 – 2.88 (m, 3H), 2.24 (s, 3H) ppm; ^{13}C NMR (100 MHz, DMSO) δ 177.6, 170.5, 153.2, 147.9, 146.8, 145.1, 143.4, 139.1, 138.8, 136.7, 132.7, 130.1, 129.2, 127.1, 109.3, 107.9, 106.4, 101.7, 73.0, 70.6, 60.5, 56.5, 50.8, 47.8, 44.2, 43.7, 32.6, 21.2 ppm. HRMS (ESI-TOF) *m/z* Calcd for $C_{35}H_{36}N_3O_9$ [M-Br]$^+$ 642.2450, found 642.2446.

a20

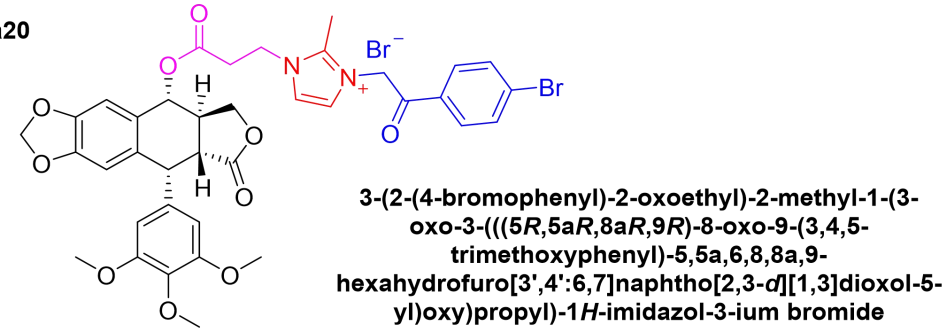

3-(2-(4-bromophenyl)-2-oxoethyl)-2-methyl-1-(3-oxo-3-(((5*R*,5a*R*,8a*R*,9*R*)-8-oxo-9-(3,4,5-trimethoxyphenyl)-5,5a,6,8,8a,9-hexahydrofuro[3',4':6,7]naphtho[2,3-*d*][1,3]dioxol-5-yl)oxy)propyl)-1*H*-imidazol-3-ium bromide

2.2.17 3-(2-(4-bromophenyl)-2-oxoethyl)-2-methyl-1-(3-oxo-3-(((5*R*,5a*R*,8a*R*,9*R*)-8-oxo-9-(3,4,5-trimethoxyphenyl)-5,5a,6,8,8a,9-hexahydrofuro[3',4':6,7]naphtho[2,3-*d*][1,3]dioxol-5-yl)oxy)propyl)-1*H*-imidazol-3-ium bromide (a20)

Yield 58%. White powder. m.p. 199 - 200 °C. IR ν_{max} (cm^{-1}): 3443, 2251, 2125, 1662, 1175, 1031,

822, 760, 624. ^{1}H NMR (400 MHz, DMSO-d_6) δ 8.06 (d, J = 8.8 Hz, 2H), 7.94 (d, J = 8.8 Hz, 2H), 7.78 (d, J = 2.4 Hz, 1H), 7.67 (d, J = 2.0 Hz, 1H), 6.91 (s, 1H), 6.59 (d, J = 26.4 Hz, 3H), 6.09 (d, J = 28.8 Hz, 4H), 5.80 (d, J = 6.0 Hz, 1H), 4.52 – 4.46 (m, 3H), 4.39 – 4.33 (m, 2H), 3.80 (s, 6H), 3.70 (s, 4H), 3.15 – 2.99 (m, 3H), 2.65 (s, 3H) ppm; ^{13}C NMR (100 MHz, DMSO) δ 191.0, 177.7, 170.6, 153.2, 147.9, 146.7, 146.5, 138.7, 136.7, 133.3, 132.8, 132.6, 130.8, 129.1, 127.3, 123.2, 121.6, 109.3, 107.8, 106.4, 101.8, 72.9, 70.5, 60.5, 56.5, 54.9, 44.1, 43.8, 43.7, 33.7, 9.8 ppm. HRMS (ESI-TOF) m/z Calcd for $C_{37}H_{36}BrN_2O_{10}$ $[M-Br]^+$ 747.1544, found 747.1548.

a21

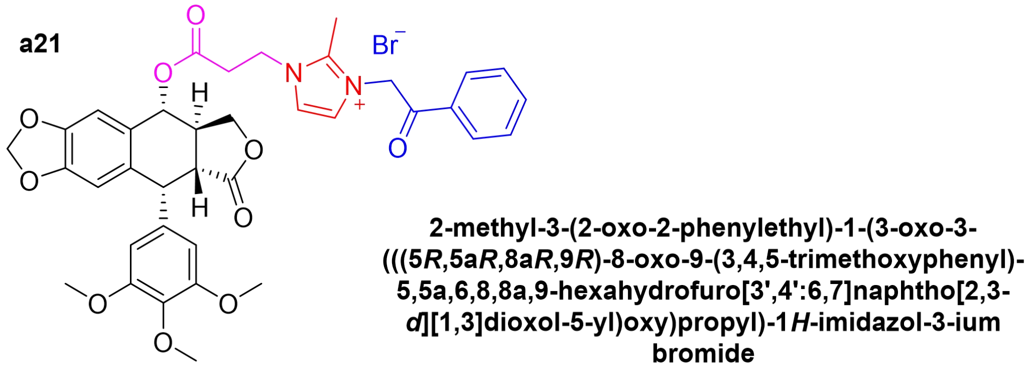

2-methyl-3-(2-oxo-2-phenylethyl)-1-(3-oxo-3-(((5*R*,5a*R*,8a*R*,9*R*)-8-oxo-9-(3,4,5-trimethoxyphenyl)-5,5a,6,8,8a,9-hexahydrofuro[3',4':6,7]naphtho[2,3-*d*][1,3]dioxol-5-yl)oxy)propyl)-1*H*-imidazol-3-ium bromide

2.2.18 2-methyl-3-(2-oxo-2-phenylethyl)-1-(3-oxo-3-(((5*R*,5a*R*,8a*R*,9*R*)-8-oxo-9-(3,4,5-trimethoxyphenyl)-5,5a,6,8,8a,9-hexahydrofuro[3',4':6,7]naphtho[2,3-*d*][1,3]dioxol-5-yl)oxy)propyl)-1*H*-imidazol-3-ium bromide (a21)

Yield 50%. White powder. m.p. 203 - 205 °C. IR ν_{max} (cm^{-1}): 3435, 2253, 2127, 1661, 1485, 1238, 1027, 824, 761, 626. ^{1}H NMR (400 MHz, DMSO-d_6) δ 7.97 (d, J = 7.2 Hz, 2H), 7.73 – 7.68 (m, 2H), 7.59 – 7.55 (m, 3H), 6.92 (s, 1H), 6.55 (s, 1H), 6.27 (s, 2H), 6.02 (s, 2H), 5.97 (d, J = 4.8 Hz, 2H), 5.87 (d, J = 9.2 Hz, 1H), 4.51 (d, J = 4.8 Hz, 1H), 4.48 – 4.37 (m, 2H), 4.23 (t, J = 6.8 Hz, 1H), 4.14 – 4.09 (m, 1H), 3.58 (s, 6H), 3.53 (s, 3H), 3.34 (dd, J = 14.8, 4.8 Hz, 1H), 3.19 – 3.00 (m, 2H), 2.75 – 2.65 (m, 2H), 2.55 (s, 3H) ppm; ^{13}C NMR (100 MHz, DMSO) δ 191.6, 174.3, 171.4, 152.5, 147.9, 147.4, 146.5, 136.9, 136.1, 135.0, 134.2, 132.8, 129.5, 128.85, 128.81, 123.2, 121.7, 109.8, 108.6, 107.6, 101.9, 73.8, 71.1, 60.4, 56.2, 54.9, 44.5, 43.8, 43.3, 33.8, 9.9 ppm. HRMS (ESI-TOF) m/z Calcd for $C_{37}H_{37}N_2O_{10}$ $[M-Br]^+$ 669.2444, found 669.2443.

2.3 Synthesis of compounds b1-b5

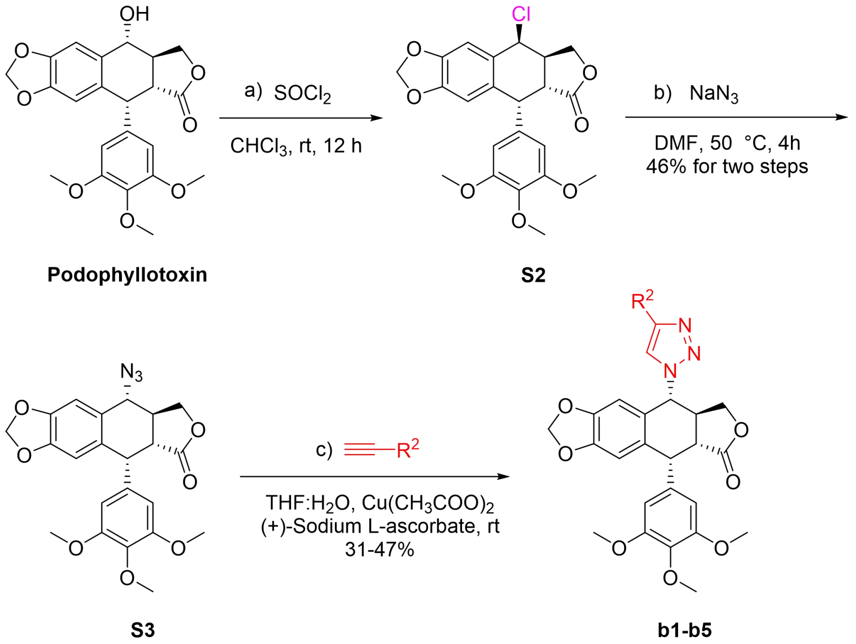

To solution of podophyllotoxin (1.0 g, 2.41 mmoL) in trichloroethane was added sulfurous dichloride (0.57 g, 4.82 mmoL) at 0 ºC. The resulting mixture was stirred at room temperat ure for 12 h. After quenching the reaction with water , the layers were separated. The organ ic phase was dried over anhydrous Na_2SO_4 and concentrated for the next synthetic step. A mixture of the previous sulfurous dichloride (2.59 mmoL) was stirred in N,N-dimethylformam ide (10.0 mL) at reflux for 4 h (monitored by TLC). After cooling to room temperature, the solvent was concentrated. The organic layer was washed with water and brine, dried over a nhydrous Na_2SO_4 and concentrated. The residue was purified by column chromatography (sili ca gel, petroleum : ethyl acetate = 7:1) to afford **S3** in 46% yield as yellow powder. To sol ution of S3 (0.1 g, 0.26 mmoL) in tetrahydrofuran (8 mL) and water (2 mL) was added alk yne compounds (0.46mmoL), copper acetate (62 mg, 0.34 mmoL) and sodium ascorbate (90 mg, 0.45 mmoL). The resulting mixture was stirred at room temperature for 12-36 h (monito red by TLC). The solvent was concentrated, and the residue was diluted with EtOAc. The or ganic layer was washed with water and brine, dried over anhydrous Na_2SO_4 and concentrated. The residue was purified by column chromatography (silica gel, dichloromethane : ethyl acet ate = 3:1) to afford **b1-b5** in 31–47% yield as white powder.

b1

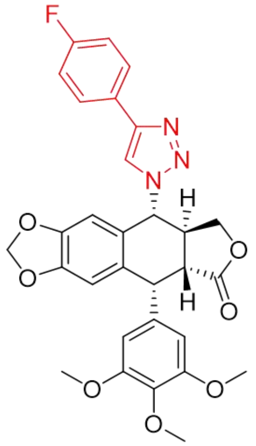

(5*R*,5a*R*,8a*S*,9*R*)-9-(4-(4-fluorophenyl)-1*H*-1,2,3-triazol-1-yl)-5-(3,4,5-trimethoxyphenyl)-5,8,8a,9-tetrahydrofuro[3',4':6,7]naphtho[2,3-*d*][1,3]dioxol-6(5a*H*)-one

2.3.1 (5*R*,5a*R*,8a*S*,9*R*)-9-(4-(4-fluorophenyl)-1*H*-1,2,3-triazol-1-yl)-5-(3,4,5-trimethoxyphenyl)-5,8,8a,9-tetrahydrofuro[3',4':6,7]naphtho[2,3-*d*][1,3]dioxol-6(5a*H*)-one (b1)

Yield 31%. White powder. m.p. 130 - 133 °C. IR ν_{max} (cm^{-1}): 1275, 1260, 913, 748. ^{1}H NMR (600 MHz, Chloroform-*d*) δ 7.79 – 7.77 (m, 2H), 7.52 (s, 1H), 7.11 (t, *J* = 8.4 Hz, 2H), 6.74 (s, 1H), 6.37 (s, 2H), 6.27 (s, 1H), 6.00 (s, 1H), 5.97 (s, 1H), 5.85 (d, *J* = 4.8 Hz, 1H), 4.64 (s, 1H), 4.59 – 4.56 (m, 1H), 4.43 (dd, *J* = 10.8, 4.2 Hz, 1H), 3.85 (s, 3H), 3.81 (s, 6H), 3.69 – 3.64 (m, 1H), 3.55 (d, *J* = 10.2 Hz, 1H) ppm; ^{13}C NMR (150 MHz, $CDCl_3$) δ 176.5, 162.7, 161.0, 152.8, 147.6, 147.0, 146.1, 137.1, 136.4, 129.0, 126.64, 126.59, 125.21, 125.19, 125.1, 119.2, 115.0, 114.8, 109.3, 105.6, 103.7, 100.7, 68.3, 59.9, 58.2, 55.4, 44.3, 43.9, 37.3 ppm. HRMS (ESI-TOF) *m/z* Calcd for $C_{30}H_{27}FN_3O_7$ $[M+H]^+$ 560.1824, found 560.1828.

b2

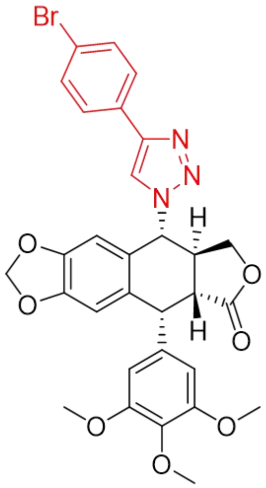

(5*R*,5a*R*,8a*S*,9*R*)-9-(4-(4-bromophenyl)-1*H*-1,2,3-triazol-1-yl)-5-(3,4,5-trimethoxyphenyl)-5,8,8a,9-tetrahydrofuro[3',4':6,7]naphtho[2,3-*d*][1,3]dioxol-6(5a*H*)-one

2.3.2 (5*R*,5a*R*,8a*S*,9*R*)-9-(4-(4-bromophenyl)-1*H*-1,2,3-triazol-1-yl)-5-(3,4,5-trimethoxyphenyl)-5,8,8a,9-tetrahydrofuro[3',4':6,7]naphtho[2,3-*d*][1,3]dioxol-6(5a*H*)-one (b2)

Yield 47%. White powder. m.p. 131 - 132 °C. IR ν_{max} (cm^{-1}): 3726, 1780, 1275, 1128, 913,764, 456. ^{1}H NMR (600 MHz, Chloroform-*d*) δ 7.66 (d, *J* = 6.6 Hz, 2H), 7.53 (d, *J* = 6.0 Hz, 2H), 7.41 (s, 1H), 6.66 (d, *J* = 5.4 Hz, 2H), 6.32 (s, 2H), 6.14 (d, *J* = 4.2 Hz, 1H), 6.03 (s, 1H), 6.01 (s, 1H), 4.77 (d, *J* = 5.4 Hz, 1H), 4.43 (t, *J* = 8.4 Hz, 1H), 3.82 (s, 3H), 3.77 (s, 6H), 3.37 (t, *J* = 10.2 Hz, 1H), 3.29 – 3.22 (m, 1H), 3.08 (dd, *J* = 12.6, 3.0 Hz, 1H) ppm; ^{13}C NMR (150 MHz, $CDCl_3$) δ 172.0, 151.9, 148.5, 147.2, 145.9, 136.7, 133.1, 132.3, 131.1, 127.8, 126.3, 123.6, 121.5, 118.8, 109.6, 107.9, 107.3, 101.0, 66.5, 59.8, 57.8, 55.4, 42.7, 40.7, 36.1 ppm. HRMS (ESI-TOF) *m/z* Calcd for $C_{30}H_{26}BrN_3O_7$

$[M+H]^+$ 620.1029, found 620.1027.

b3

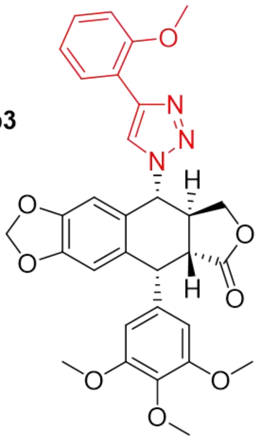

(5*R*,5a*R*,8a*S*,9*R*)-9-(4-(2-methoxyphenyl)-1*H*-1,2,3-triazol-1-yl)-5-(3,4,5-trimethoxyphenyl)-5,8,8a,9-tetrahydrofuro[3',4':6,7]naphtho[2,3-*d*][1,3]dioxol-6(5a*H*)-one

2.3.3 (5*R*,5a*R*,8a*S*,9*R*)-9-(4-(2-methoxyphenyl)-1*H*-1,2,3-triazol-1-yl)-5-(3,4,5-trimethoxyphenyl)-5,8,8a,9-tetrahydrofuro[3',4':6,7]naphtho[2,3-*d*][1,3]dioxol-6(5a*H*)-one (b3)

Yield 33%. White powder. m.p. 137 - 139 °C. IR ν_{max} (cm^{-1}): 2939, 1781, 1487, 1239, 1127, 913, 742, 655. ^1H NMR (600 MHz, Chloroform-*d*) δ 8.32 (d, *J* = 7.8 Hz, 1H), 7.74 (s, 1H), 7.32 (t, *J* = 7.8 Hz, 1H), 7.08 (t, *J* = 7.8 Hz, 1H), 6.96 (d, *J* = 8.4 Hz, 1H), 6.67 (d, *J* = 3.6 Hz, 2H), 6.35 (s, 2H), 6.11 (d, *J* = 4.8 Hz, 1H), 6.01 (s, 1H), 5.98 (s, 1H), 4.81 (d, *J* = 4.8 Hz, 1H), 4.40 (t, *J* = 7.8 Hz, 1H), 3.88 (s, 3H), 3.82 (s, 3H), 3.77 (s, 6H), 3.35 – 3.21 (m, 3H) ppm; ^{13}C NMR (150 MHz, $CDCl_3$) δ 172.4, 154.6, 151.8, 148.2, 147.0, 142.4, 136.6, 133.4, 132.1, 128.4, 126.7, 124.2, 122.0, 120.2, 117.7, 109.8, 109.4, 107.9, 107.3, 100.9, 66.4, 59.8, 57.5, 55.4, 54.5, 42.7, 40.8, 36.3 ppm. HRMS (ESI-TOF) *m/z* Calcd for $C_{31}H_{30}N_3O_8$ $[M+H]^+$ 572.2022, found 572.2027.

b4

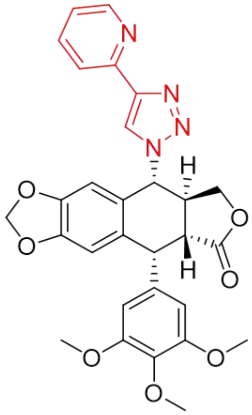

(5*R*,5a*R*,8a*S*,9*R*)-9-(4-(pyridin-2-yl)-1*H*-1,2,3-triazol-1-yl)-5-(3,4,5-trimethoxyphenyl)-5,8,8a,9-tetrahydrofuro[3',4':6,7]naphtho[2,3-*d*][1,3]dioxol-6(5a*H*)-one

2.3.4 (5*R*,5a*R*,8a*S*,9*R*)-9-(4-(pyridin-2-yl)-1*H*-1,2,3-triazol-1-yl)-5-(3,4,5-trimethoxyphenyl)-5,8,8a,9-tetrahydrofuro[3',4':6,7]naphtho[2,3-*d*][1,3]dioxol-6(5a*H*)-one (b4)

Yield 38%. White powder. m.p. 126 - 128 °C. IR ν_{max} (cm^{-1}): 1780, 1594, 1486, 1421, 1238, 1127, 913, 743, 655. ^1H NMR (600 MHz, Chloroform-*d*) δ 8.20 (d, *J* = 4.8 Hz, 1H), 7.86 (d, *J* = 7.8 Hz, 1H), 7.50 – 7.45 (m, 2H), 6.92 (s, 1H), 6.31 (d, *J* = 5.4 Hz, 2H), 5.99 (s, 2H), 5.84 (s, 1H), 5.68 (s, 1H), 5.65 (s, 1H), 4.41 (d, *J* = 5.4 Hz, 1H), 4.12 – 4.09 (m, 1H), 3.48 (s, 3H), 3.43 (s, 6H), 3.05 –

3.02 (m, 1H), 2.96 – 2.90 (m, 1H), 2.77 – 2.74 (m, 1H) ppm; ^{13}C NMR (150 MHz, $CDCl_3$) δ 172.0, 151.8, 148.7, 148.6, 148.4, 147.4, 147.2, 136.6, 136.2, 133.2, 132.3, 123.4, 122.3, 121.2, 119.4, 109.5, 107.9, 107.2, 101.0, 66.4, 59.8, 57.9, 55.3, 42.7, 40.5, 36.2 ppm. HRMS (ESI-TOF) *m/z* Calcd for $C_{29}H_{26}N_4O_7$ $[M+H]^+$ 543.1879, found 543.1874.

b5

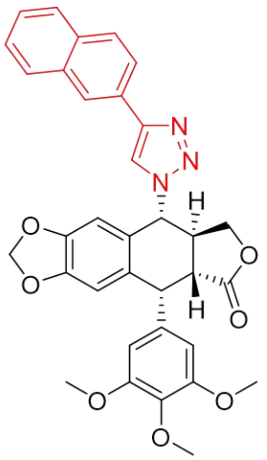

(5*R*,5a*R*,8a*S*,9*R*)-9-(4-(naphthalen-2-yl)-1*H*-1,2,3-triazol-1-yl)-5-(3,4,5-trimethoxyphenyl)-5,8,8a,9-tetrahydrofuro[3',4':6,7]naphtho[2,3-*d*][1,3]dioxol-6(5a*H*)-one

2.3.5 (5*R*,5a*R*,8a*S*,9*R*)-9-(4-(naphthalen-2-yl)-1*H*-1,2,3-triazol-1-yl)-5-(3,4,5-trimethoxyphenyl)-5,8,8a,9-tetrahydrofuro[3',4':6,7]naphtho[2,3-*d*][1,3]dioxol-6(5a*H*)-one (b5)

Yield 43%. White powder. m.p. 128 - 129 °C. IR ν_{max} (cm^{-1}): 2936, 1780, 1589, 1486, 1419, 1332, 1239, 1126, 1036, 913, 743, 476. ^{1}H NMR (600 MHz, Chloroform-*d*) δ 8.23 (s, 1H), 7.81 (s, 2H), 7.80 – 7.76 (m, 2H), 7.46 (s, 1H), 7.43 – 7.41 (m, 2H), 6.62 (d, J = 9.0 Hz, 2H), 6.27 (s, 2H), 6.11 (d, J = 4.8 Hz, 1H), 5.96 (s, 1H), 5.94 (s, 1H), 4.73 (d, J = 4.8 Hz, 1H), 4.40 - 4.37 (m, 1H), 3.76 (s, 3H), 3.71 (s, 6H), 3.36 - 3.33 (m, 1H), 3.24 – 3.17 (m, 1H), 3.08 (dd, J = 14.4, 4.8 Hz, 1H) ppm; ^{13}C NMR (150 MHz, $CDCl_3$) δ 172.1, 151.9, 148.5, 147.2, 147.0, 136.7, 133.2, 132.5, 132.3, 127.7, 127.2, 126.8, 126.2, 125.6, 125.4, 123.7, 123.6, 122.7, 119.0, 109.6, 108.0, 107.3, 101.0, 66.5, 59.8, 57.8, 55.4, 42.7, 40.8, 36.2 ppm. HRMS (ESI-TOF) *m/z* Calcd for $C_{34}H_{30}N_3O_7$ $[M+H]^+$ 592.2080, found 592.2078.

2.4 Synthesis of compounds c1-c3

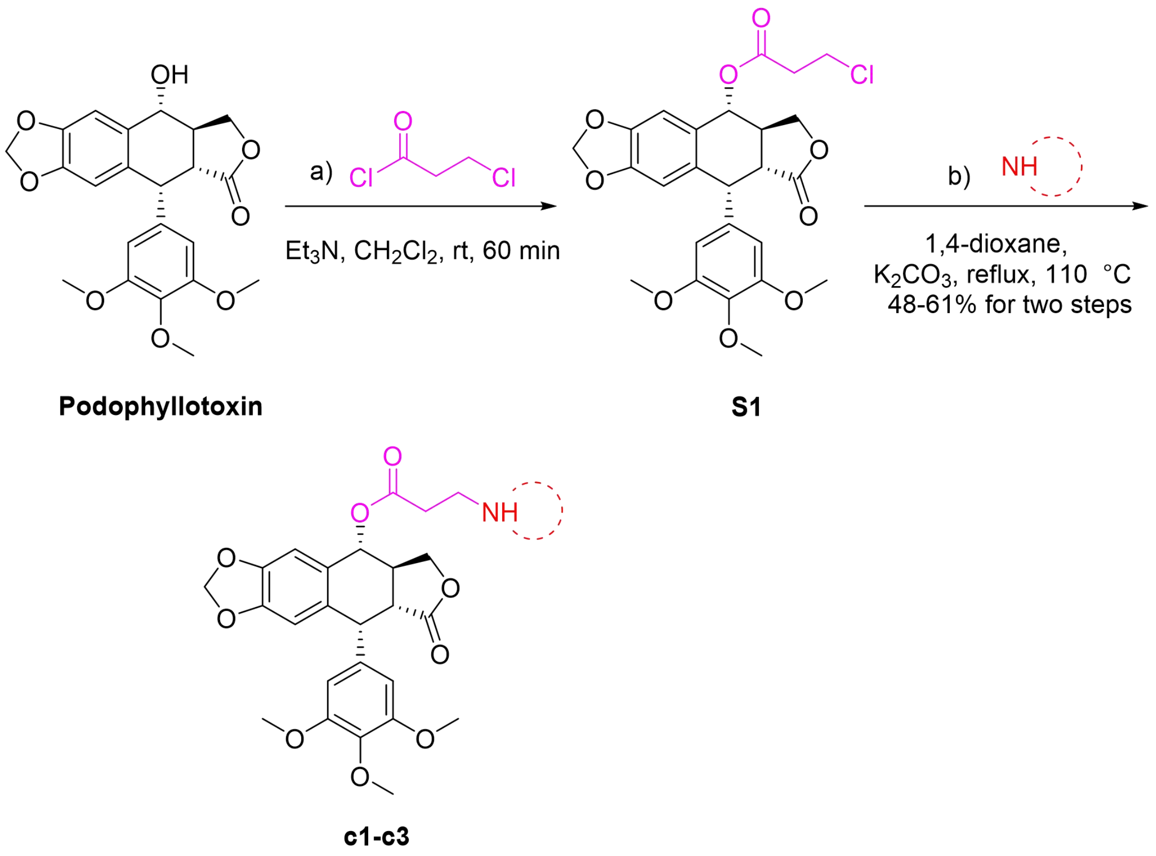

Scheme 3 Synthesis of nitrogenous derivatives of podophyllotoxin **c1-c3**.

To a solution of podophyllotoxin (1.0 g, 2.41 mmoL) in dichloromethane (10.0 mL) was added 3-chloropropanoyl chloride (0.77 g, 6.03 mmoL) and triethylamine (0.98 g, 9.65 mmoL) at 0 ºC. The resulting mixture was stirred at room temperature for 1 h. After quenching the reaction with water (50.0 mL), the layers were separated. The organic phase was dried over anhydrous Na_2SO_4 and concentrated, and used for the next synthetic step. A mixture of the previous 3-chloropropanoyl chloride **S1**(0.1g, 0.20 mmoL), K_2CO_3(0.77g, 7.23mmoL) and amines(0.40 mmoL) was stirred in 1,4-dioxane(2 mL) at reflux for 24-36 h (monitored by TLC). After cooling to room temperature, the solvent was concentrated, and the residue was diluted with dichloromethane. The organic layer was washed with water and brine, dried over anhydrous Na_2SO_4 and concentrated. The residue was purified by column chromatography (silica gel, ethyl acetate: methanol = 7:1) to afford **c1-c3** in 48–61% yield (two steps) as white powder.

c1

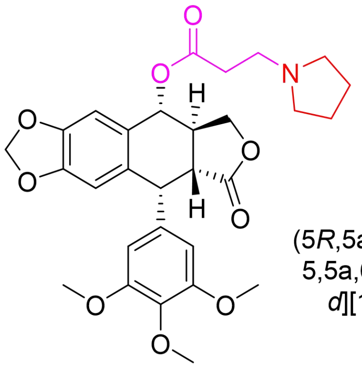

(5*R*,5a*R*,8a*R*,9*R*)-8-oxo-9-(3,4,5-trimethoxyphenyl)-5,5a,6,8,8a,9-hexahydrofuro[3',4':6,7]naphtho[2,3-*d*][1,3]dioxol-5-yl 3-(pyrrolidin-1-yl)propanoate

2.4.1 (5*R*,5a*R*,8a*R*,9*R*)-8-oxo-9-(3,4,5-trimethoxyphenyl)-5,5a,6,8,8a,9-hexahydrofuro[3',4':6,7] naphtho[2,3-*d*][1,3]dioxol-5-yl 3-(pyrrolidin-1-yl)propanoate (c1)

Yield 58%. White powder. m.p. 141 - 148 °C. IR ν_{max} (cm^{-1}): 1775, 1591, 1485, 1243, 1126, 913, 744, 655. ^{1}H NMR (600 MHz, Chloroform-*d*) δ 6.82 (s, 1H), 6.40 (s, 1H), 6.34 (s, 2H), 5.89 (s, 1H), 5.86 (s, 1H), 5.71 (d, *J* = 6.0 Hz, 1H), 4.34 – 4.29 (m, 2H), 4.24 (s, 1H), 3.77 (s, 3H), 3.74 (s, 6H), 3.17 (dd, *J* = 9.0, 3.6 Hz, 1H), 2.89 – 2.85 (m, 1H), 2.72 – 2.64 (m, 2H), 2.49 – 2.42 (m, 6H), 1.70 (s, 4H) ppm; ^{13}C NMR (150 MHz, $CDCl_3$) δ 176.2, 171.2, 152.4, 147.1, 146.2, 137.9, 130.1, 125.9, 108.5, 106.7, 104.7, 100.3, 70.9, 69.5, 59.8, 55.2, 52.8, 50.4, 44.6, 43.1, 38.9, 33.4, 22.5 ppm. HRMS (ESI-TOF) *m/z* Calcd for $C_{29}H_{34}NO_9$ $[M+H]^+$ 540.2224, found 540.2228.

c2

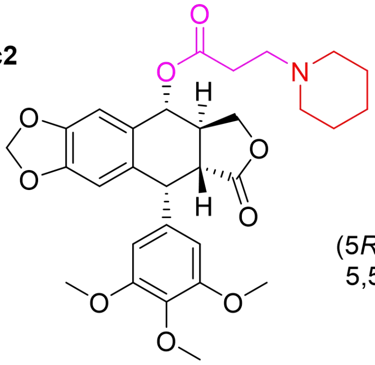

(5*R*,5a*R*,8a*R*,9*R*)-8-oxo-9-(3,4,5-trimethoxyphenyl)-5,5a,6,8,8a,9-hexahydrofuro[3',4':6,7]naphtho[2,3-*d*][1,3]dioxol-5-yl 3-(piperidin-1-yl)propanoate

2.4.2 (5*R*,5a*R*,8a*R*,9*R*)-8-oxo-9-(3,4,5-trimethoxyphenyl)-5,5a,6,8,8a,9-hexahydrofuro[3',4':6,7] naphtho[2,3-*d*][1,3]dioxol-5-yl 3-(piperidin-1-yl)propanoate (c2)

Yield 61%. White powder. m.p. 139 - 141 °C. IR ν_{max} (cm^{-1}): 2936, 1775, 1590, 1485, 1241, 1126, 1038, 913, 744,655. ^{1}H NMR (600 MHz, Chloroform-*d*) δ 6.79 (s, 1H), 6.40 (s, 1H), 6.34 (s, 2H), 5.89 (s, 1H), 5.86 (s, 1H), 5.71 (d, J = 6.6 Hz, 1H), 4.34 – 4.29 (m, 2H), 4.24 (d, *J* = 3.6 Hz, 1H), 3.76 (s, 3H), 3.73 (s, 6H), 3.17 (dd, *J* = 9.6, 4.2 Hz, 1H), 2.90 – 2.87 (m, 1H), 2.54 – 2.49 (m, 2H), 2.45 – 2.38 (m, 2H), 2.26 (d, *J* = 32.4 Hz, 4H), 1.46 (d, *J* = 6.0 Hz, 4H), 1.34 (s, 2H) ppm; ^{13}C NMR (150 MHz, $CDCl_3$) δ 176.2, 171.4, 152.3, 147.1, 146.2, 137.9, 135.8, 130.0, 125.9, 108.4, 106.6, 104.5, 100.3, 70.8, 69.6, 59.8, 55.1, 53.3, 53.2, 44.6, 43.1, 38.9, 31.6, 24.8, 23.2 ppm. HRMS (ESI-TOF) *m/z* Calcd for $C_{30}H_{35}NO_9$ $[M+H]^+$ 554.2384, found 554.2385.

c3

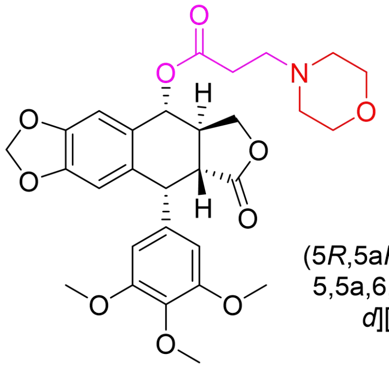

(5*R*,5a*R*,8a*R*,9*R*)-8-oxo-9-(3,4,5-trimethoxyphenyl)-5,5a,6,8,8a,9-hexahydrofuro[3',4':6,7]naphtho[2,3-*d*][1,3]dioxol-5-yl 3-morpholinopropanoate

2.4.3 (5*R*,5a*R*,8a*R*,9*R*)-8-oxo-9-(3,4,5-trimethoxyphenyl)-5,5a,6,8,8a,9-hexahydrofuro[3',4':6,7] naphtho[2,3-*d*][1,3]dioxol-5-yl 3-morpholinopropanoate (c3)

Yield 48%. White powder. m.p. 133 - 138 °C.IR ν_{max} (cm^{-1}): 2939, 1775, 1591, 1485, 1329, 1244, 1127, 1037, 913, 743, 654. ^1H NMR (600 MHz, Chloroform-*d*) δ 6.91 (s, 1H), 6.47 (s, 1H), 6.40 (s, 2H), 5.96 (s, 1H), 5.93 (s, 1H), 5.77 (d, J = 6.0 Hz, 1H), 4.41 - 4.39 (m, 1H), 4.35 (d, J = 9.6 Hz, 1H), 4.30 (d, J = 3.0 Hz, 1H), 3.83 (s, 3H), 3.80 (s, 6H), 3.67 (d, J = 5.4 Hz, 4H), 3.26 – 3.23 (m, 1H), 2.96 – 2.92 (m, 1H), 2.65 – 2.58 (m, 2H), 2.51 – 2.46 (m, 2H), 2.46 – 2.37 (m, 4H) ppm; ^{13}C NMR (150 MHz, $CDCl_3$) δ 176.1, 171.0, 152.4, 147.2, 146.2, 137.9, 136.1, 130.1, 125.8, 108.6, 106.6, 104.7, 100.4, 70.9, 69.4, 65.8, 59.8, 55.2, 53.0, 52.4, 44.5, 43.1, 38.9, 31.4 ppm. HRMS (ESI-TOF) *m/z* Calcd for $C_{29}H_{34}NO_{10}$ $[M+H]^+$ 556.2182, found 556.2177.

3. Biological Assay Procedures and Results

3.1 Biological experimental materials

The Human hepatocellular carcinoma cell lines (HepG-2), the Human pulmonary carcinoma Cell lines (A-549), Human breast carcinoma cell lines (MDA-MB-231), Human colon carcinoma cell lines (HCT-116) were purchased from the Shanghai Institute of Biochemistry and Cell Biology, Chinese Academy of Sciences (Shanghai, China). Cells were cultured in medium supplemented with 10% FBS, 100 units/mL penicillin and 100 mg/mL streptomycin (HyClone, Logan, UT, USA). All the cells were incubated at 37 °C, 5% CO_2 in a humidified atmosphere.

3.2 Cytotoxicity assay

Cytotoxicity of compounds were determined by MTS method. All the compounds tested were absolutely dissolved to 10 mM in DMSO in stock. 5×10^3 cells were plated in 96-well plates 12 h before treatment and continuously exposed to 0.032, 0.16, 0.8, 4 and 20 μM test compounds for 48 h. Then MTS (Promega, USA) was added to each well. The samples were incubated at 37 °C for 1~4 h and the optical density (OD) was measured at 490 nm using a microplate reader (Bio-Rad Laboratories). The IC_{50} values are calculated from appropriate dose-response curves.

3.3 Cell cycle analysis

To analyse the DNA content by flow cytometry, cells were collected and washed twice with PBS. Cells were fixed with 70% ethanol overnight. Fixed cells were washed with PBS, and then stained with 50 μg/mL propidium iodide (PI) solution containing 50 μg/mL RNase A for 30 mins at room temperature. Fluorescence intensity was analysed by FACSCalibur flow cytometer (BD Biosciences, San Jose, CA, USA). The percentages of the cells distributed in different phases of the cell cycle were determined using FlowJo V 7.6.5 software.

3.4 Cell apoptosis analysis

Cell apoptosis was analysed using the Annexin V-FITC/PI Apoptosis kit (BD Biosciences, Franklin Lakes, NJ) according to the manufacturer's protocols. Cells were seeded in 6-well plates at a density of 3×10^5 cells/well. After 48 h of compound treatment at the indicated concentrations, cells were collected and then washed twice ith cold PBS, and then resuspended in a binding buffer containing Annexin V-FITC and propidium iodine (PI). After incubation for 15 mins at room temperature in the dark, the fluorescent intensity was measured using a FACSCalibur flow cytometer (BD Biosciences, Franklin Lakes, NJ).

Table 2

Cytotoxic activities of podophyllotoxin nitrogen-containing heterocycles **a1–a21/b1-b5/c1-c3** in *vitro*[b] (IC_{50}, μM[a])

Entry	Compound No.	HepG-2	A-549	MDA-MB-231	HCT-116
1	**a1**	0.31±0.02	0.76±0.12	0.47±0.01	0.04±0.00
2	**a2**	0.23±0.01	0.30±0.02	0.45±0.03	0.15±0.01
3	**a3**	0.32±0.06	0.65±0.03	0.38±0.04	0.31±0.04
4	**a4**	0.33±0.01	1.11±0.04	0.53±0.01	0.04±0.00
5	**a5**	0.29±0.00	0.76±1.54	0.51±0.05	0.04±0.00

6	**a6**	0.07±0.00	0.29±0.04	0.11±0.01	0.04±0.00
7	**a7**	0.18±0.01	1.08±0.20	0.48±0.02	0.04±0.00
8	**a8**	0.26±0.01	0.65±0.39	0.55±0.02	0.04±0.00
9	**a9**	0.25±0.01	0.44±0.10	0.49±0.02	0.29±0.00
10	**a10**	0.25±0.01	0.25±0.00	0.45±0.09	0.30±0.05
11	**a11**	0.27±0.00	0.42±0.09	0.33±0.06	0.30±0.08
12	**a12**	0.26±0.05	0.53±0.10	0.51±0.02	0.21±0.01
13	**a13**	0.34±0.02	1.10±0.14	0.41±0.06	0.10±0.02
14	**a14**	0.42±0.03	1.53±0.23	0.33±0.04	0.05±0.02
15	**a15**	0.29±0.01	0.75±0.07	0.30±0.00	0.27±0.15
16	**a16**	0.28±0.02	0.75±0.07	0.25±0.02	0.20±0.06
17	**a17**	0.25±0.02	0.74±0.19	0.28±0.05	0.04±0.09
18	**a18**	0.28±0.03	0.58±0.01	0.23±0.02	0.04±0.00
19	**a19**	0.25±0.00	0.65±0.01	0.26±0.00	0.04±0.00
20	**a20**	7.98±0.51	15.84±0.04	>20	6.80±0.11
21	**a21**	0.40±0.03	0.28±0.05	0.11±0.03	0.07±0.01
22	**b1**	1.86±0.15	3.60±0.56	2.03±0.14	0.04±0.33
23	**b2**	2.14±0.04	7.31±0.12	1.74±0.47	6.58±1.87
24	**b3**	1.60±0.00	3.48±0.03	0.49±0.01	0.90±0.42
25	**b4**	4.59±0.37	9.64±0.62	7.57±0.62	1.49±1.76
26	**b5**	>20	>20	>20	>20
27	**c1**	0.28±0.01	0.58±0.02	0.04±0.03	0.05±0.01
28	**c2**	0.10±0.01	0.39±0.03	0.10±0.00	0.10±0.02
29	**c3**	0.21±0.03	0.39±0.01	0.36±0.11	0.06±0.11
30	**DDP**	1.85±0.34	5.52±0.21	12.77±2.71	10.92±0.26
31	**Etoposide**	16.95±2.00	14.77±0.26	1.92±0.96	14.19±0.13
32	**Paclitaxel**	<0.008	<0.008	<0.008	<0.008

[a] Cytotoxicity as IC_{50} for each cell line, is the concentration of compound which reduced by 50% the optical density of treated cells with respect to untreated cells using the MTS assay.

[b] Data represent the mean values of three independent determinations.

4. Copies of ^{1}H NMR, ^{13}C NMR and HR-MS (ESI) spectra

^{1}H NMR spectra (400 MHz, $CDCl_3$) of compound a1

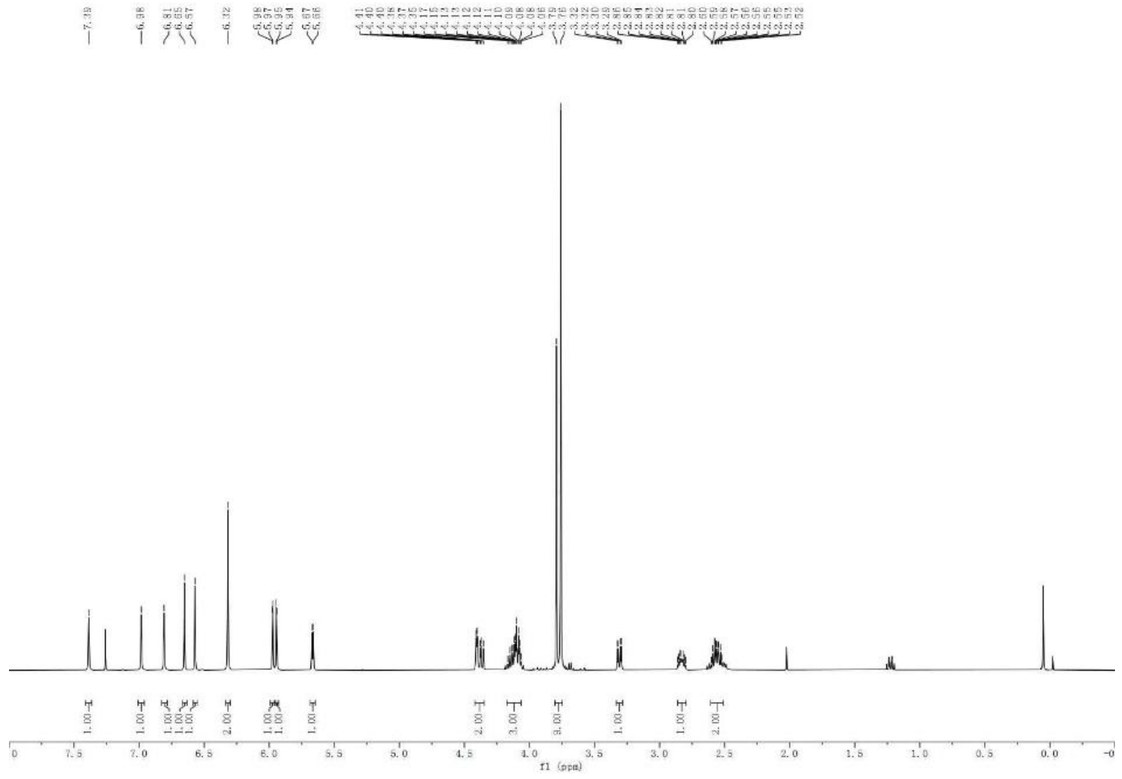

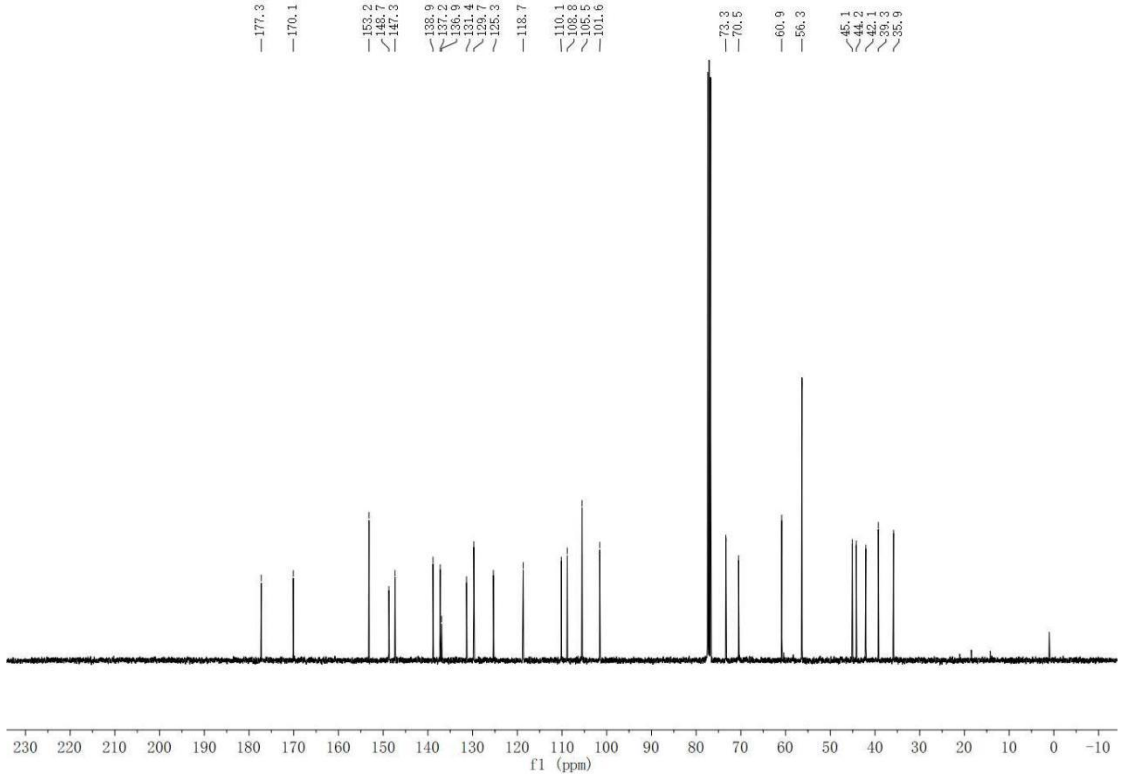

^{1}H NMR spectra (400 MHz, CDCl3) of compound a2

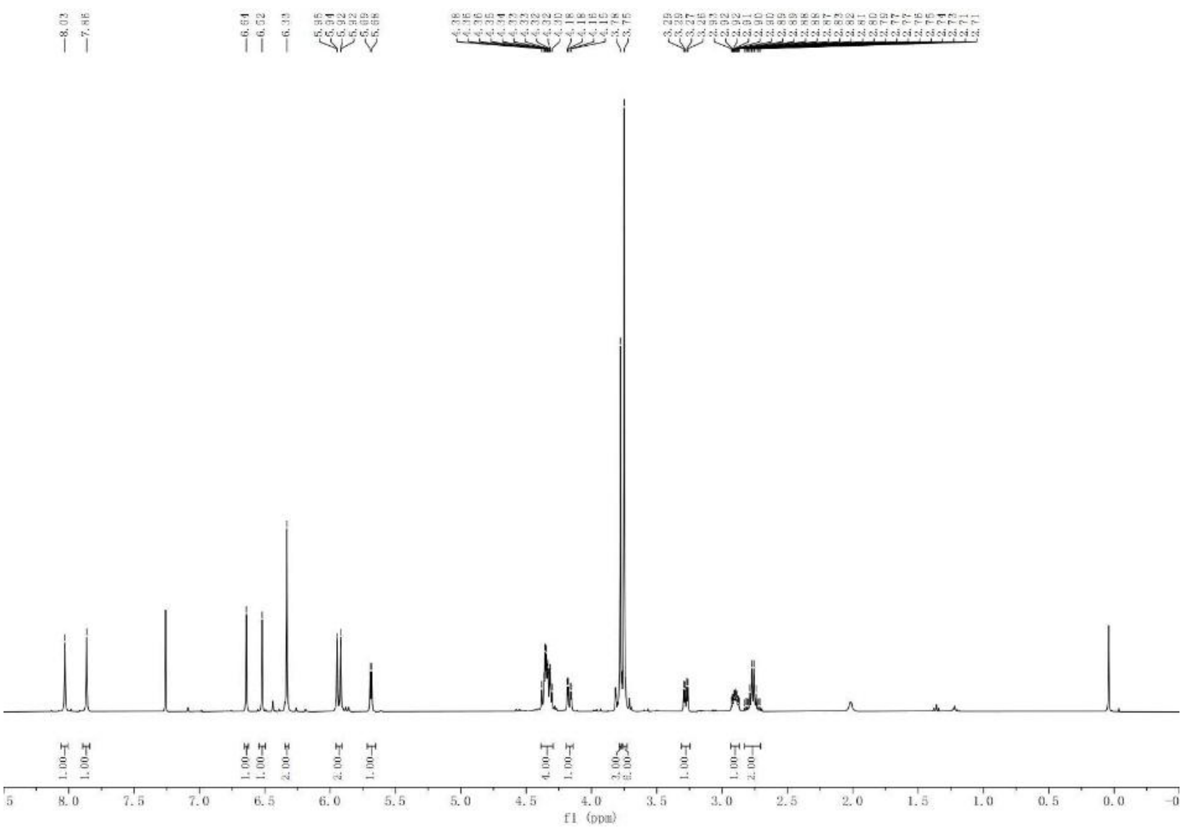

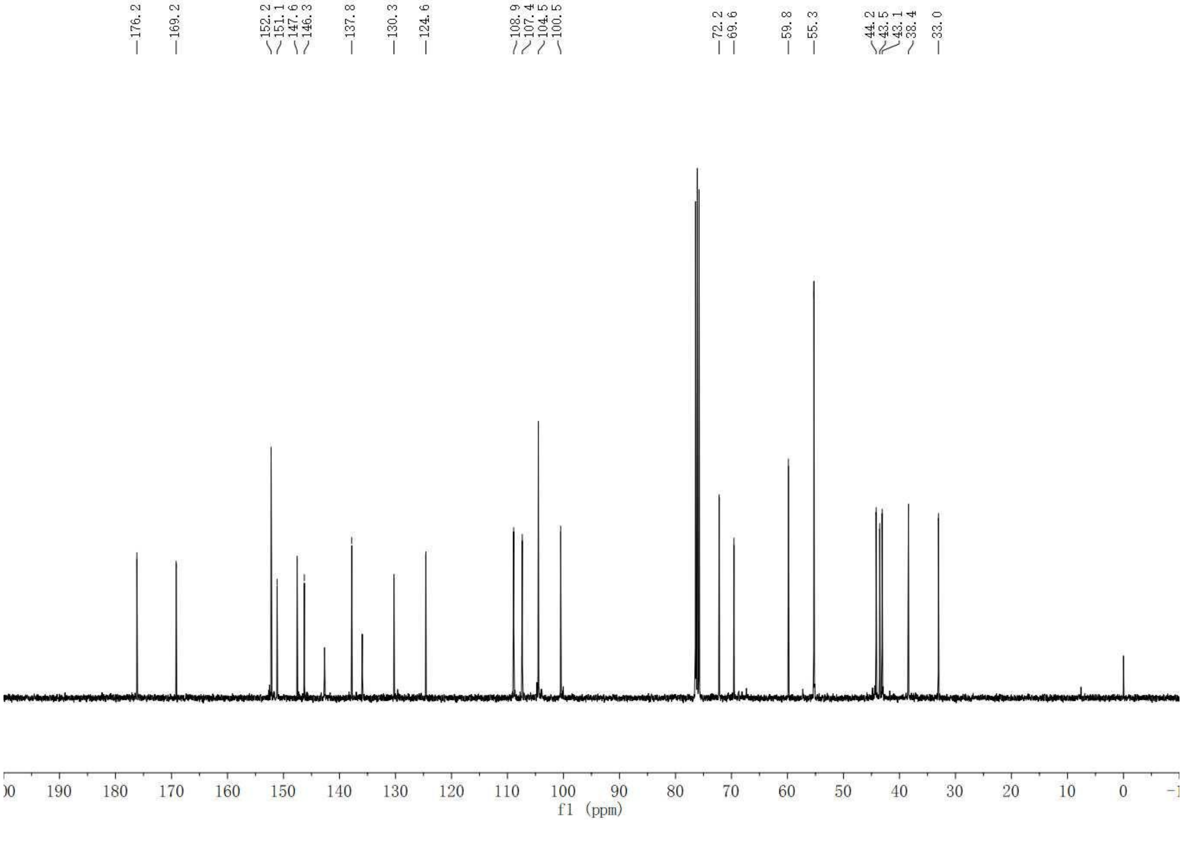

^{1}H NMR spectra (400 MHz, $CDCl_3$) of compound a3

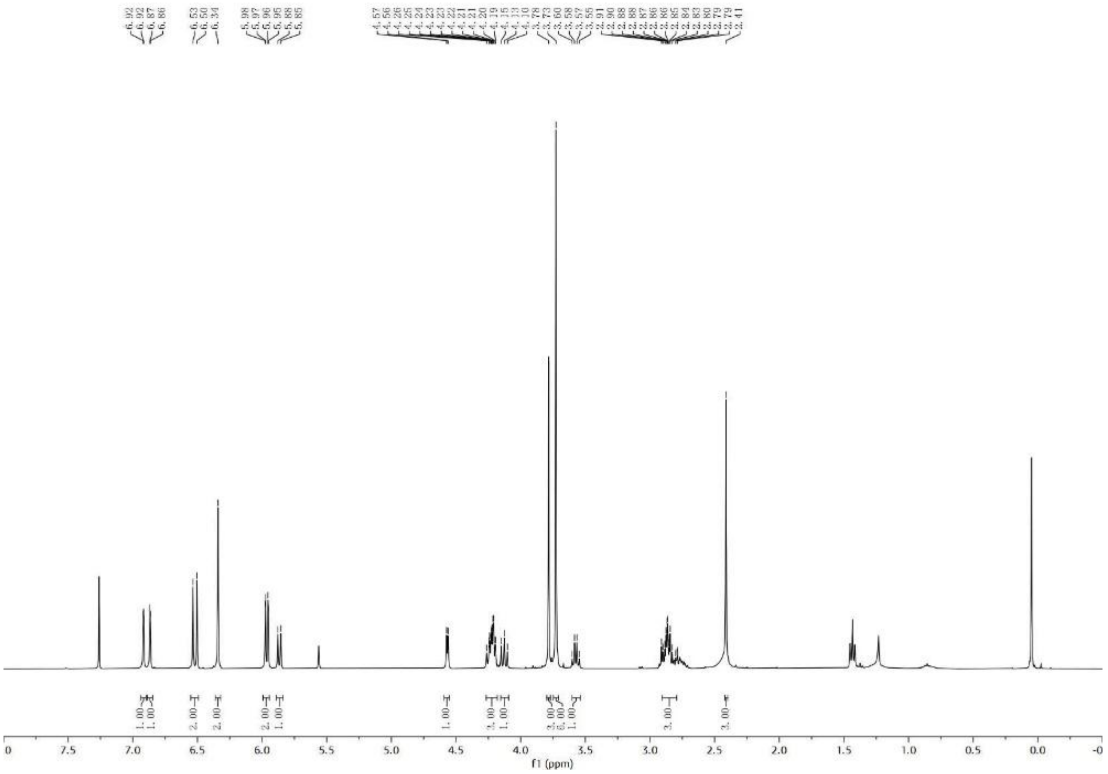

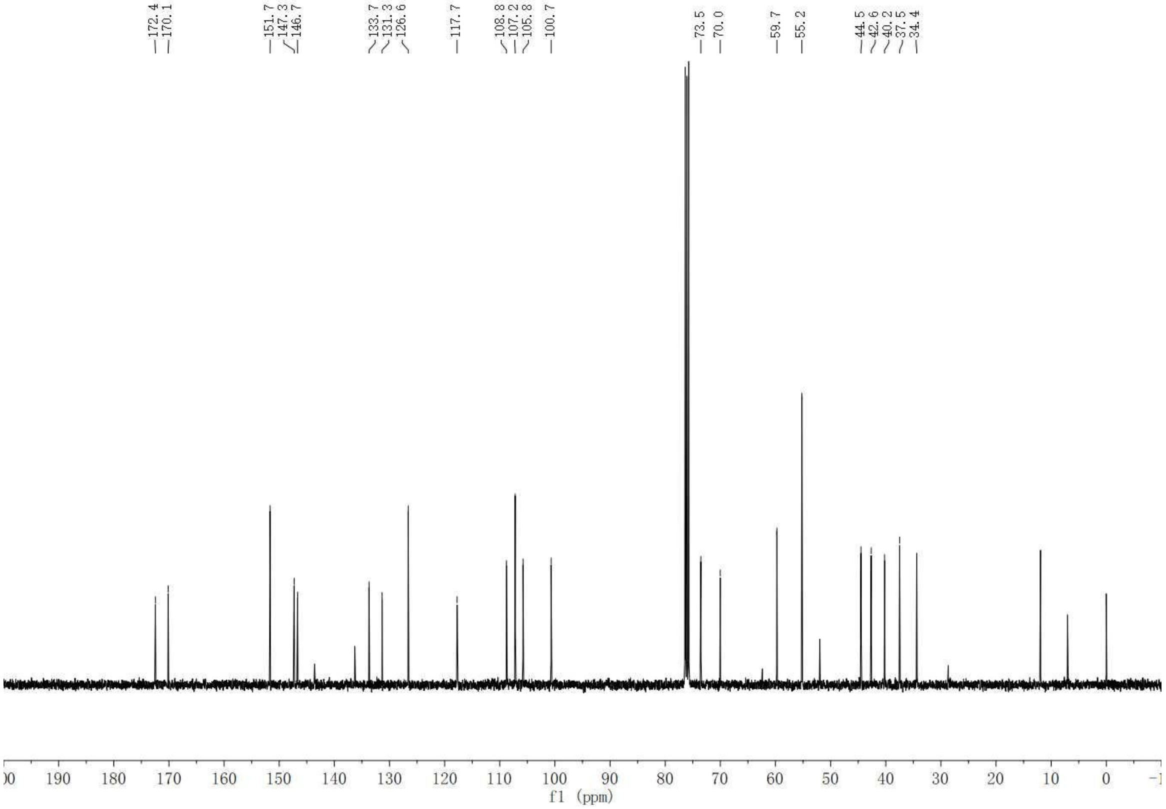

^{1}H NMR spectra (400 MHz, CDCl3) of compound a4

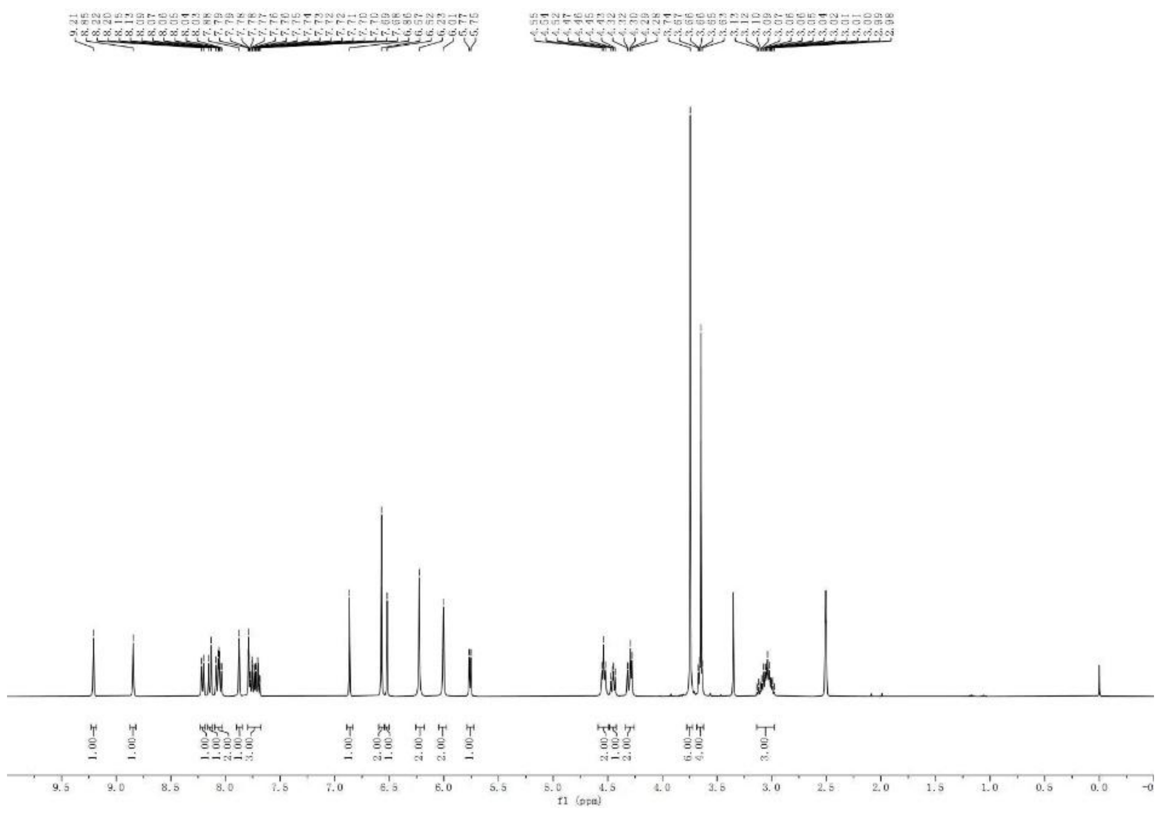

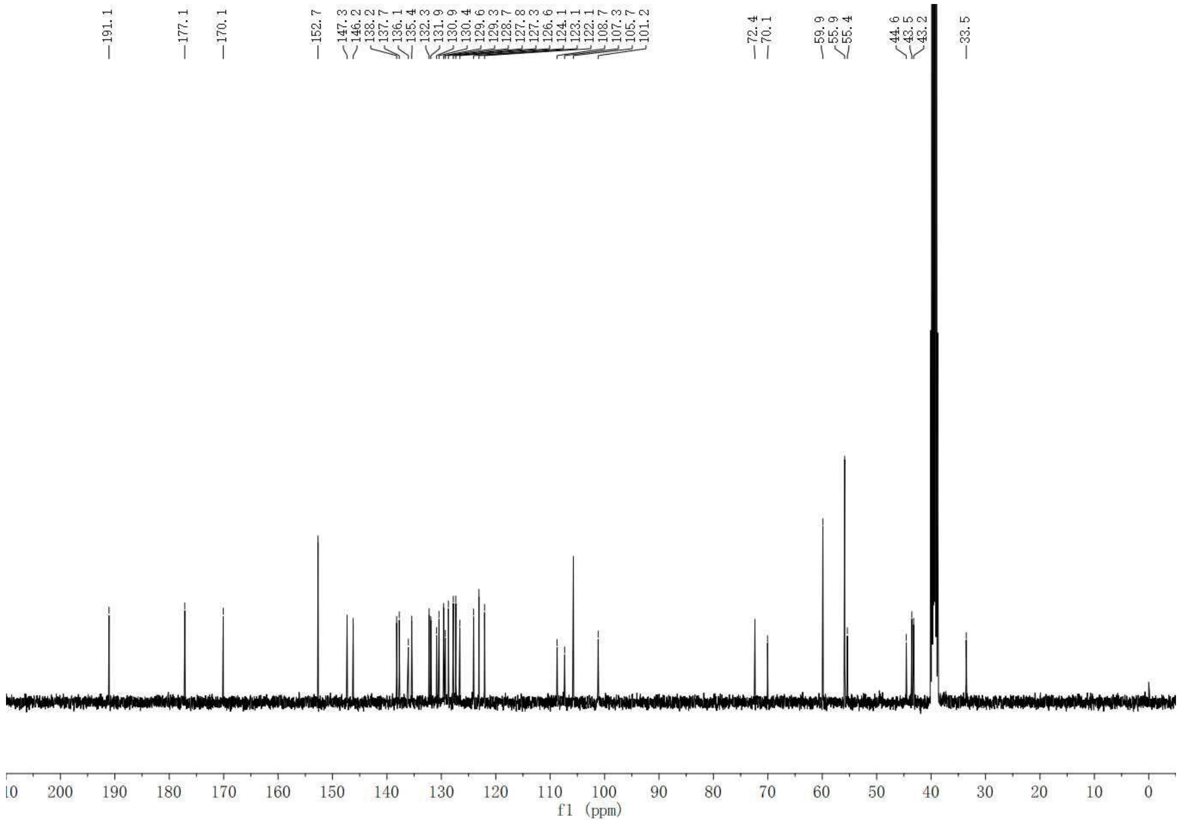

^{1}H NMR spectra (400 MHz, $CDCl_3$) of compound a5

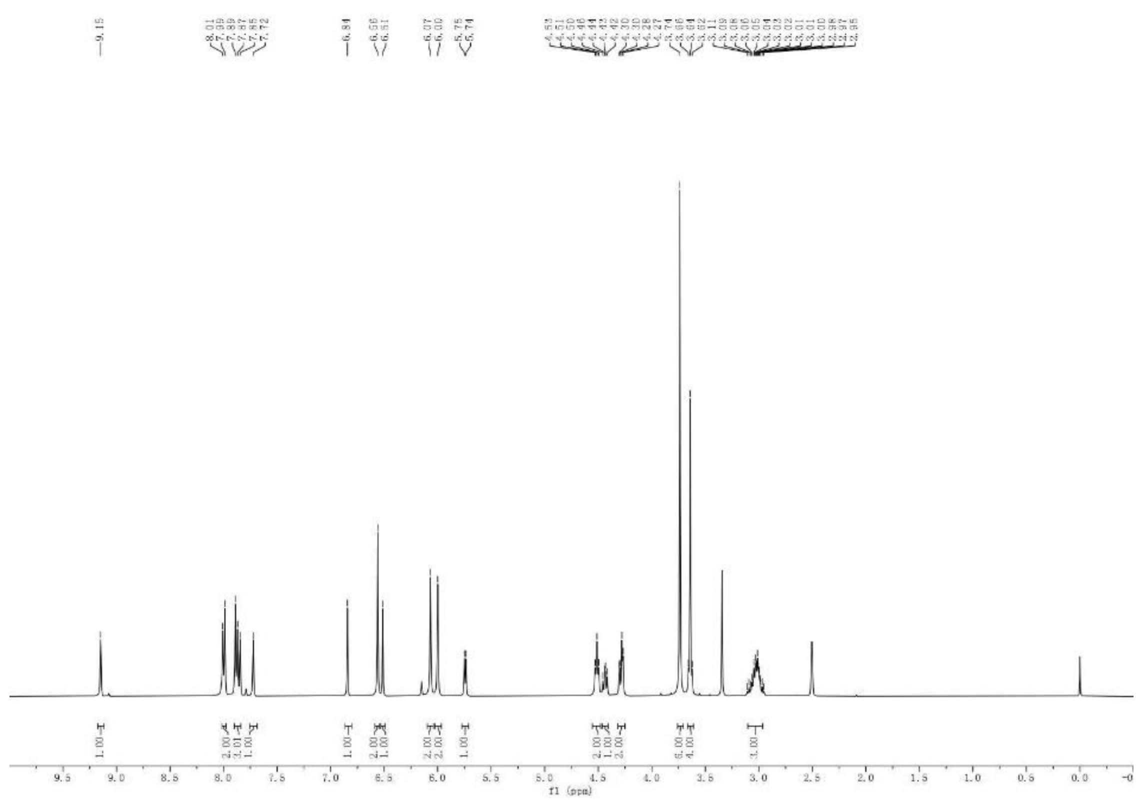

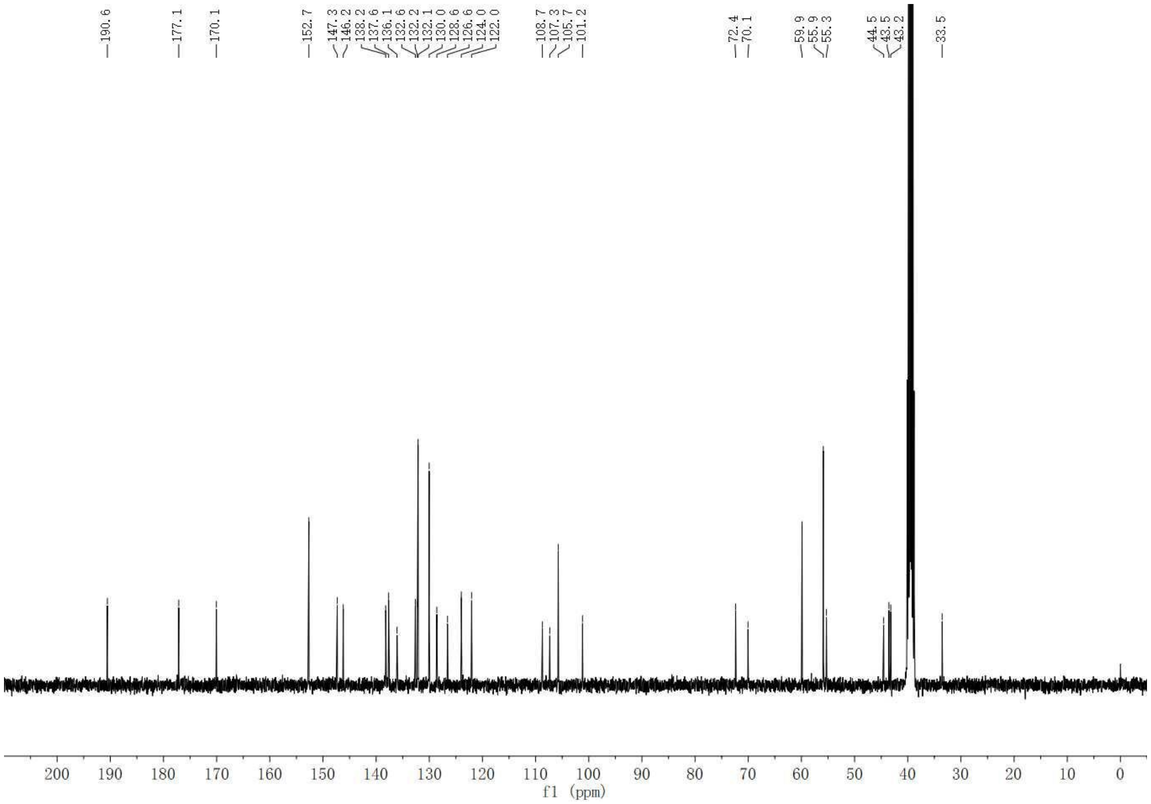

^{1}H NMR spectra (400 MHz, $CDCl_3$) of compound a6

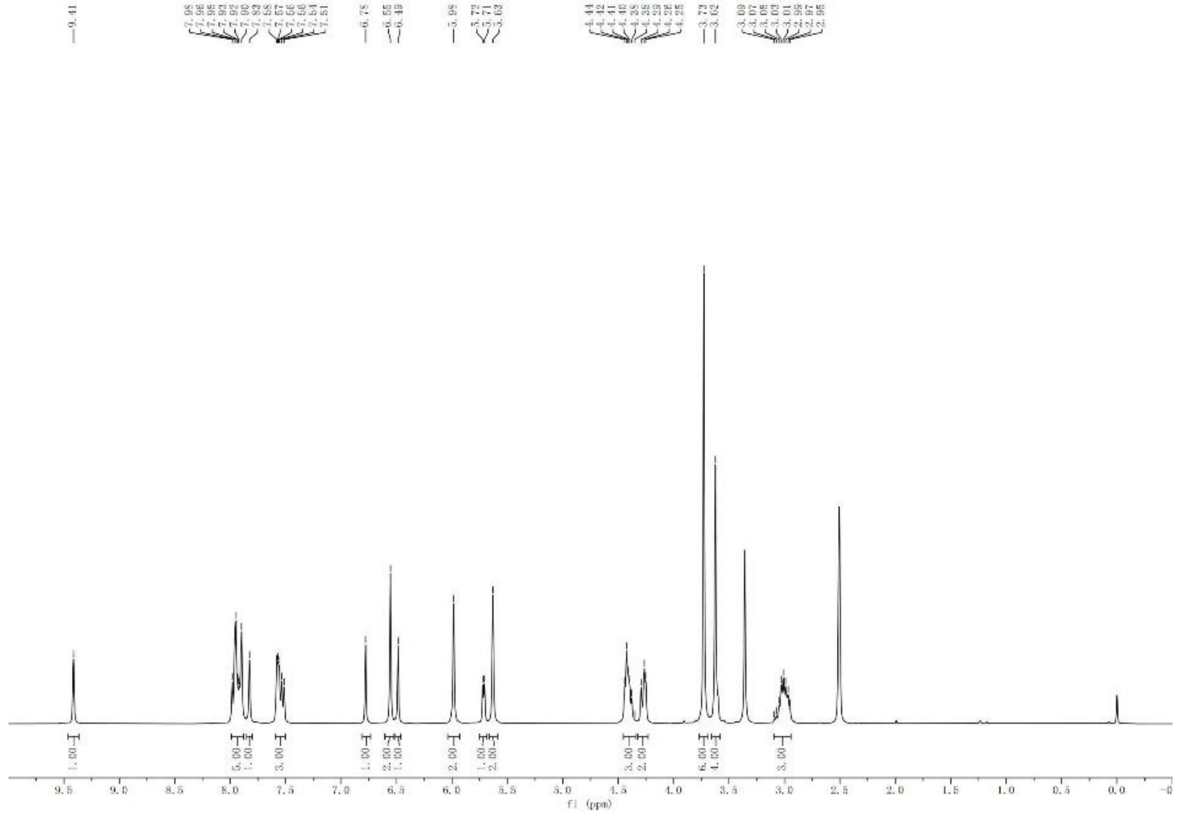

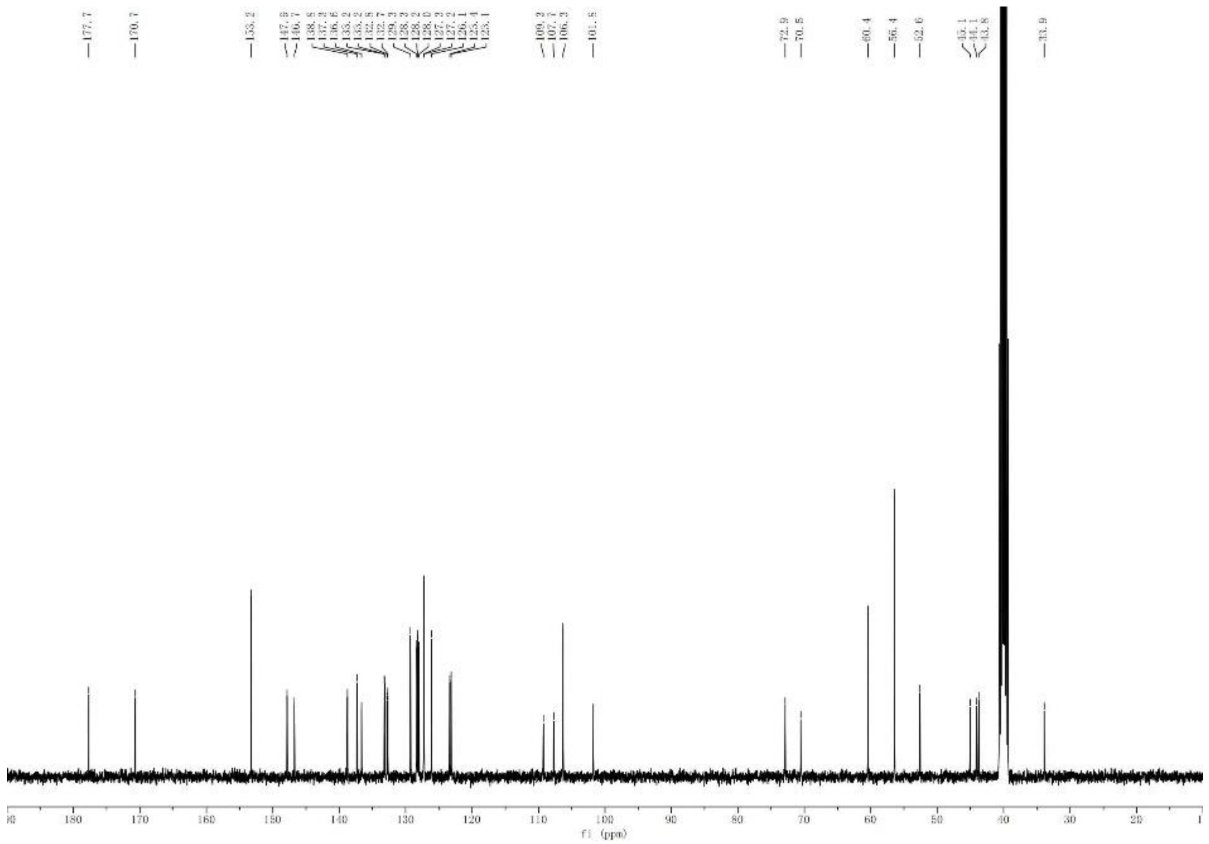

^{1}H NMR spectra (400 MHz, $CDCl_3$) of compound a7

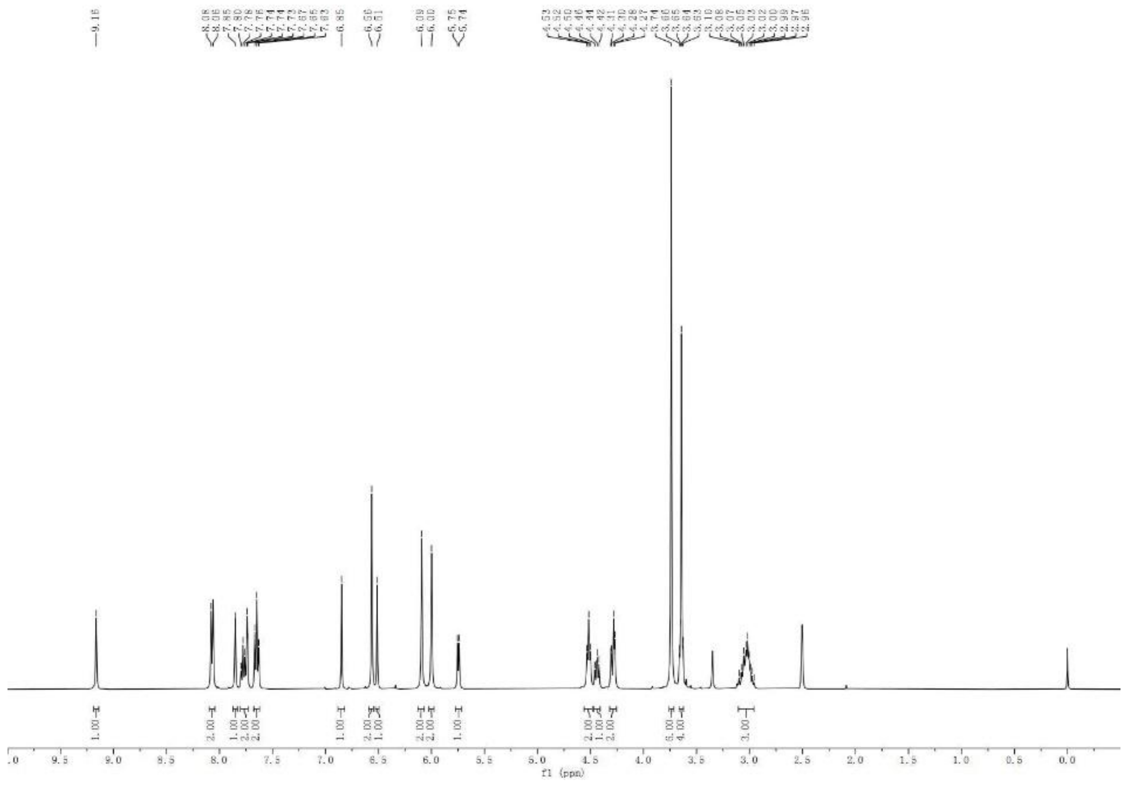

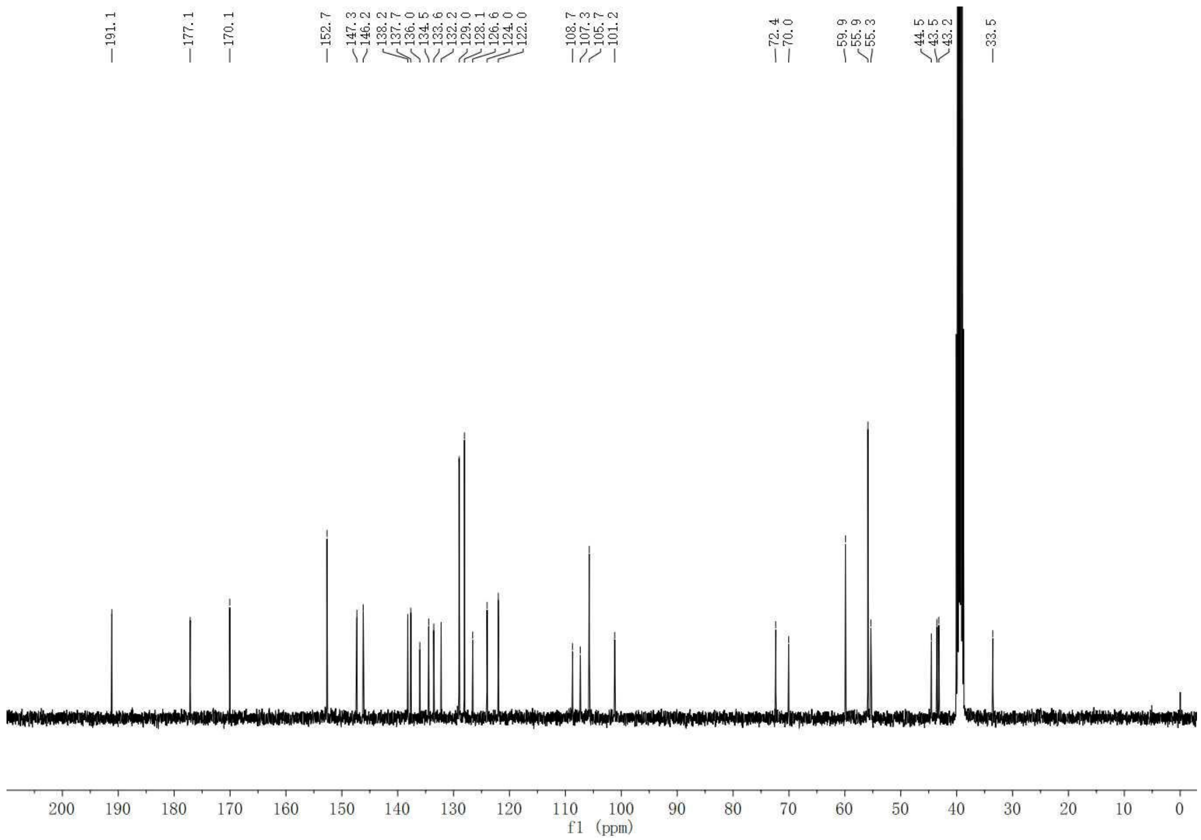

^{1}H NMR spectra (400 MHz, $CDCl_3$) of compound a8

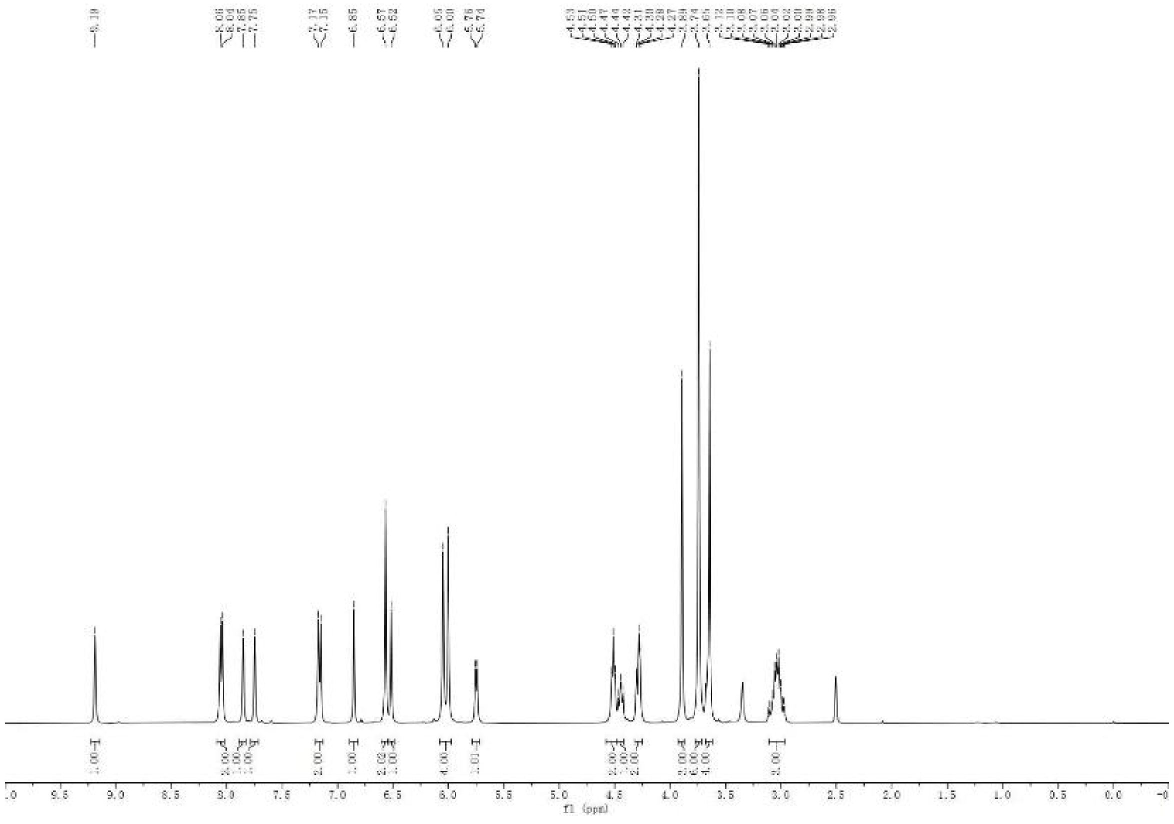

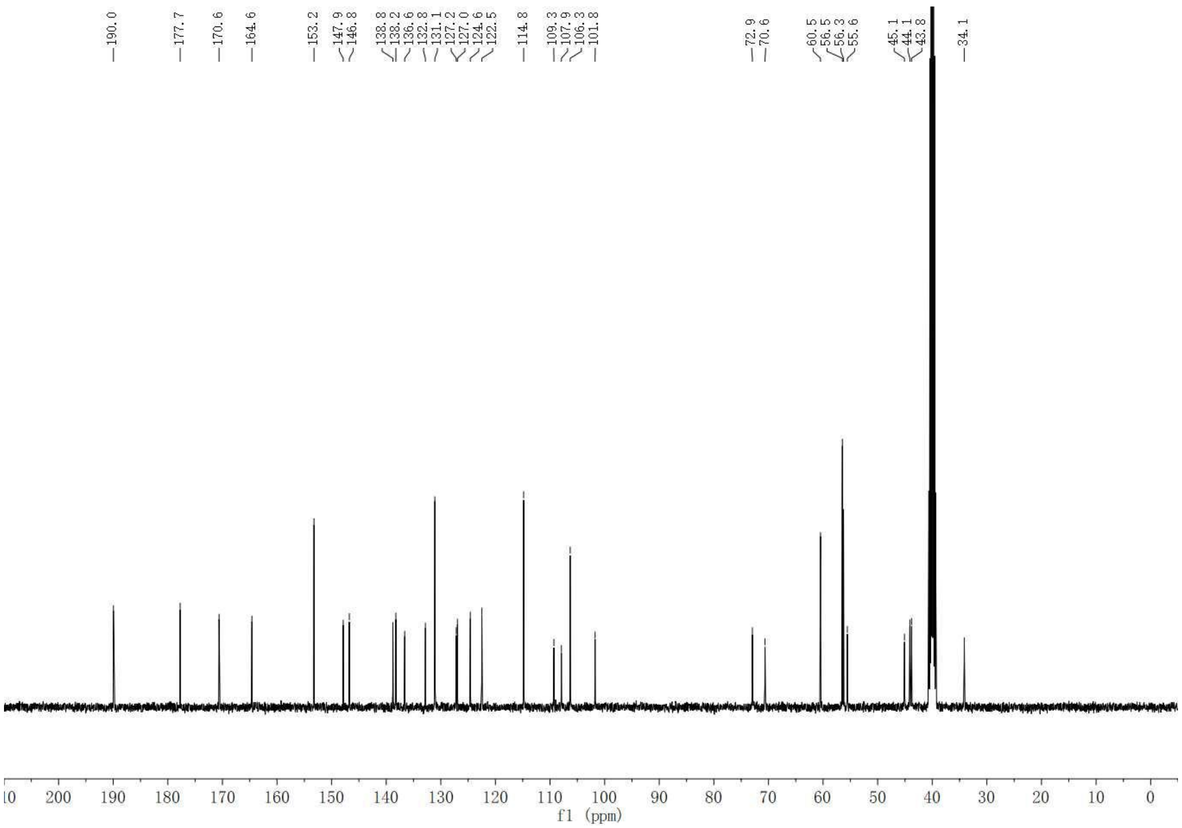

^{1}H NMR spectra (400 MHz, $CDCl_3$) of compound a9

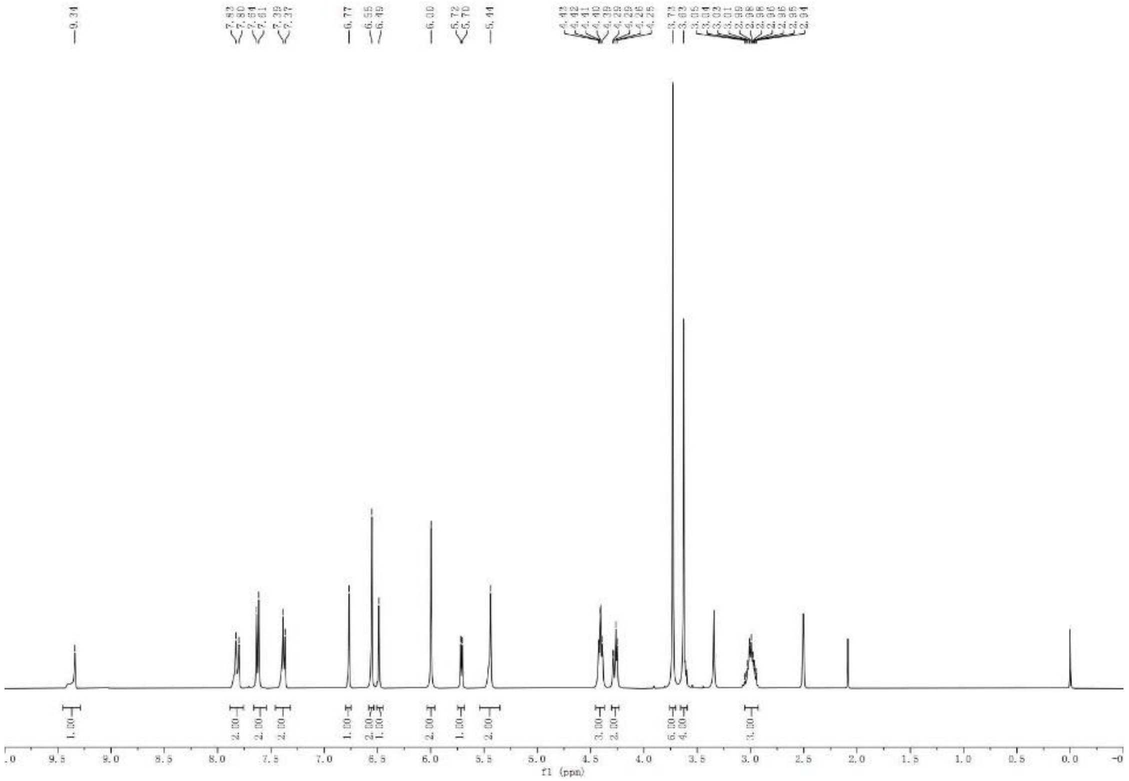

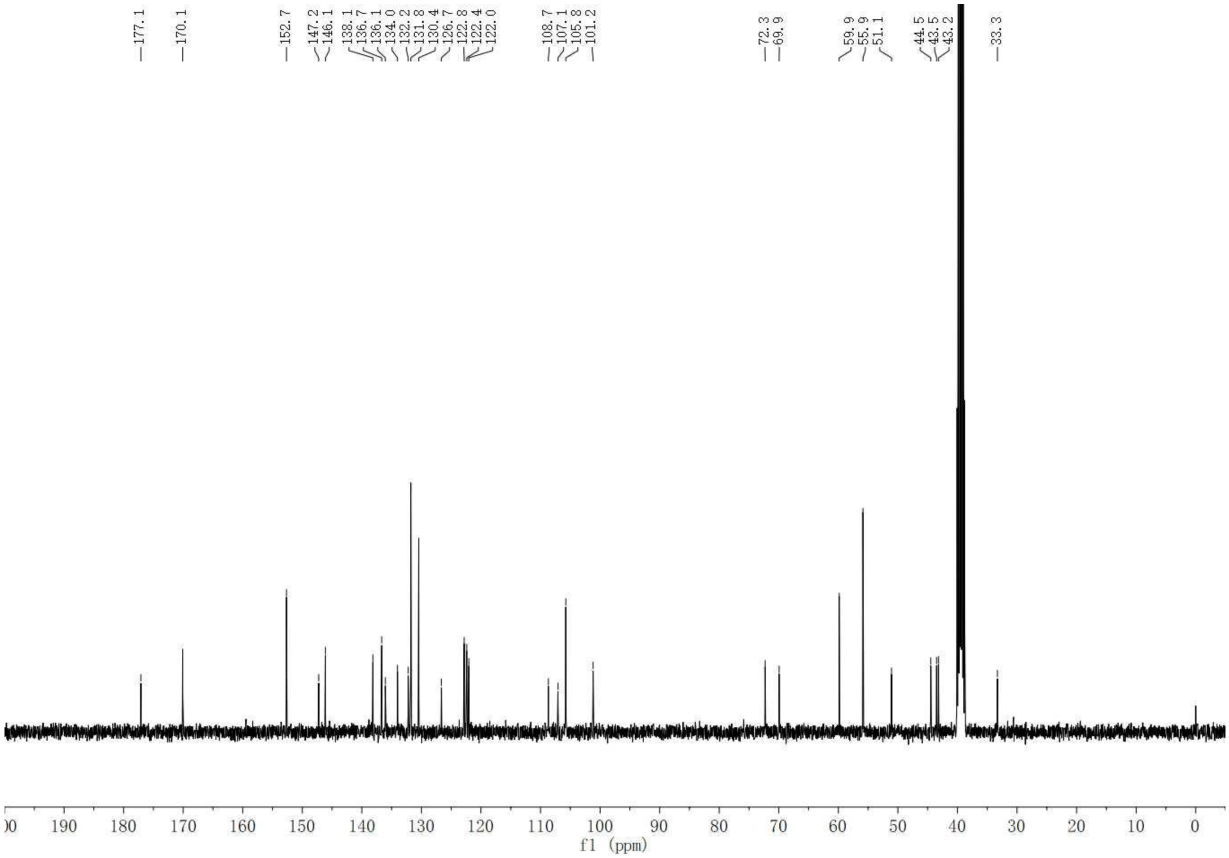

^{1}H NMR spectra (400 MHz, $CDCl_3$) of compound a10

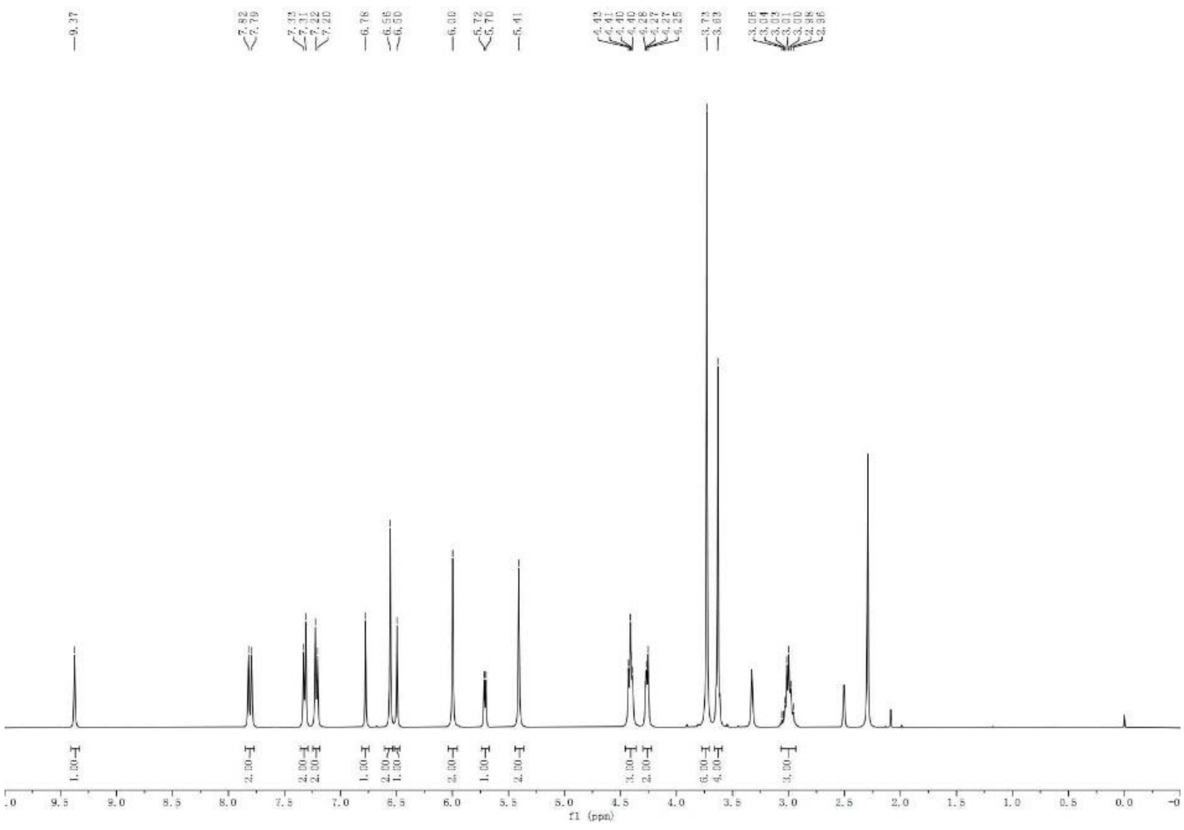

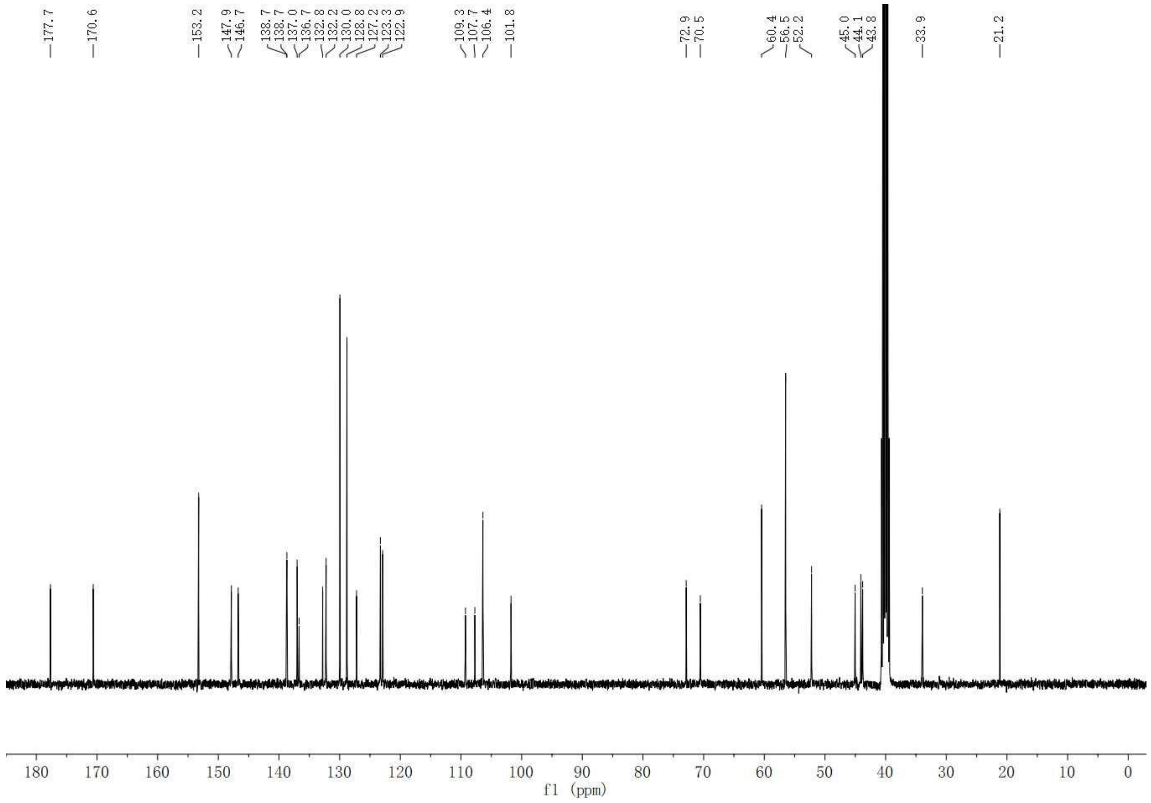

^{1}H NMR spectra (400 MHz, $CDCl_3$) of compound a11

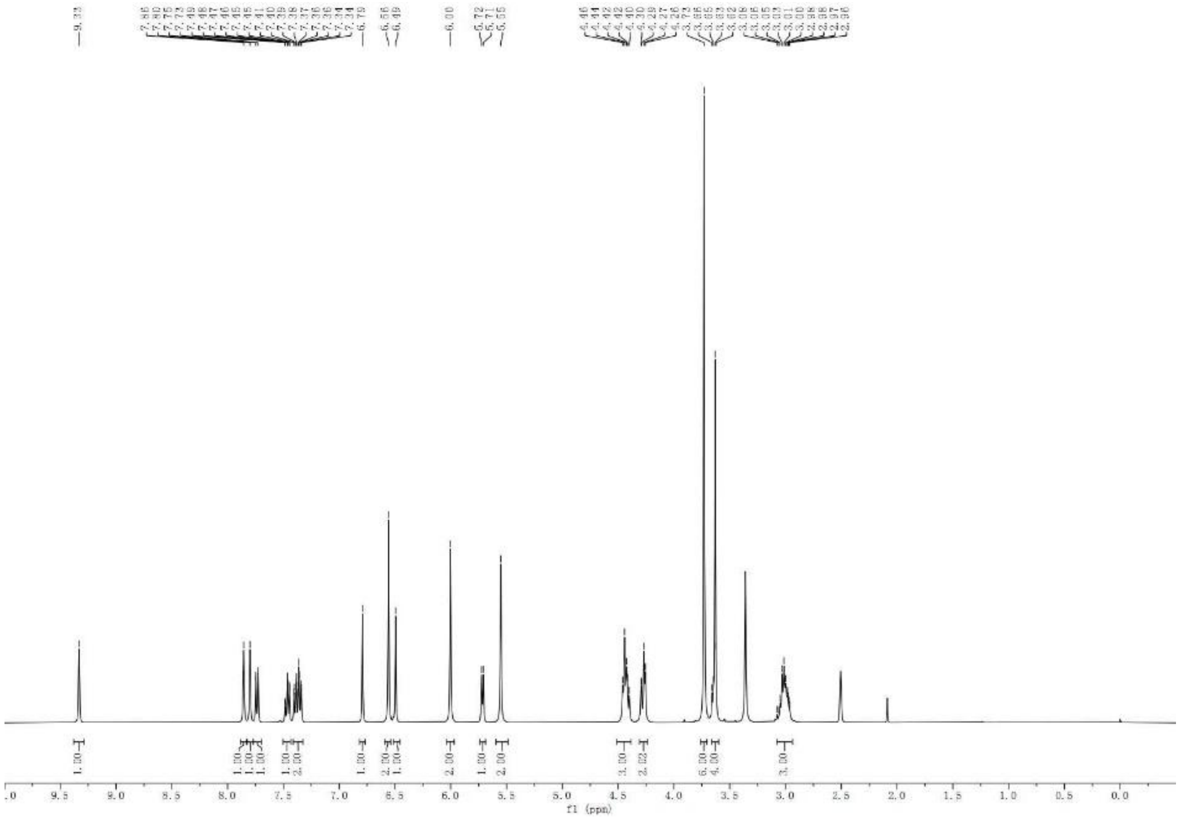

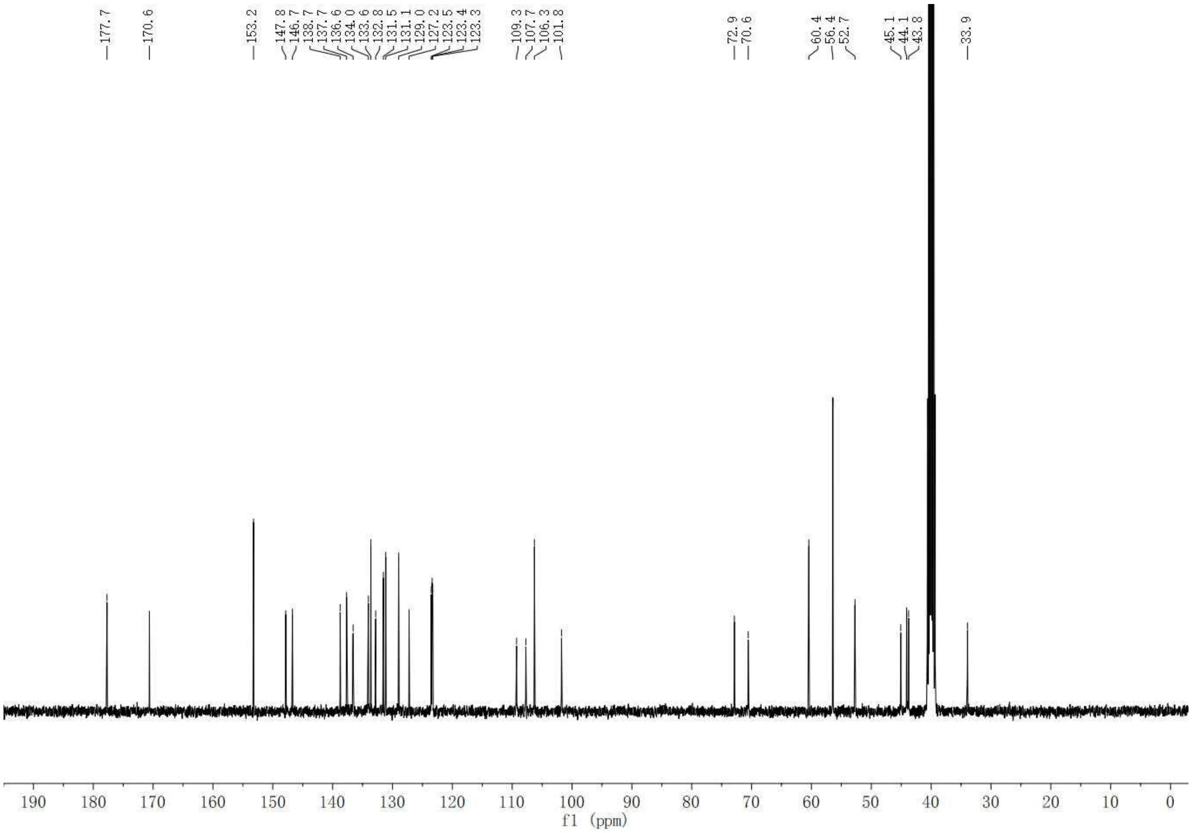

^{1}H NMR spectra (400 MHz, $CDCl_3$) of compound a12

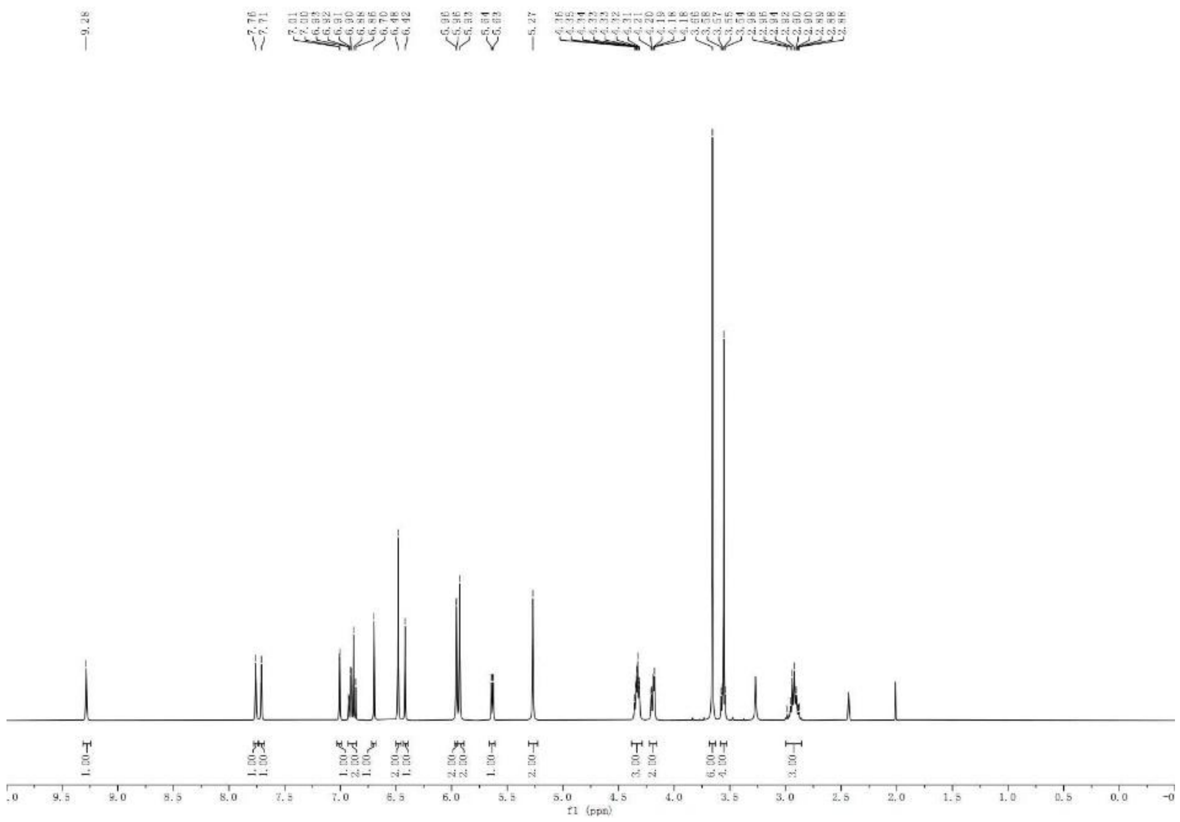

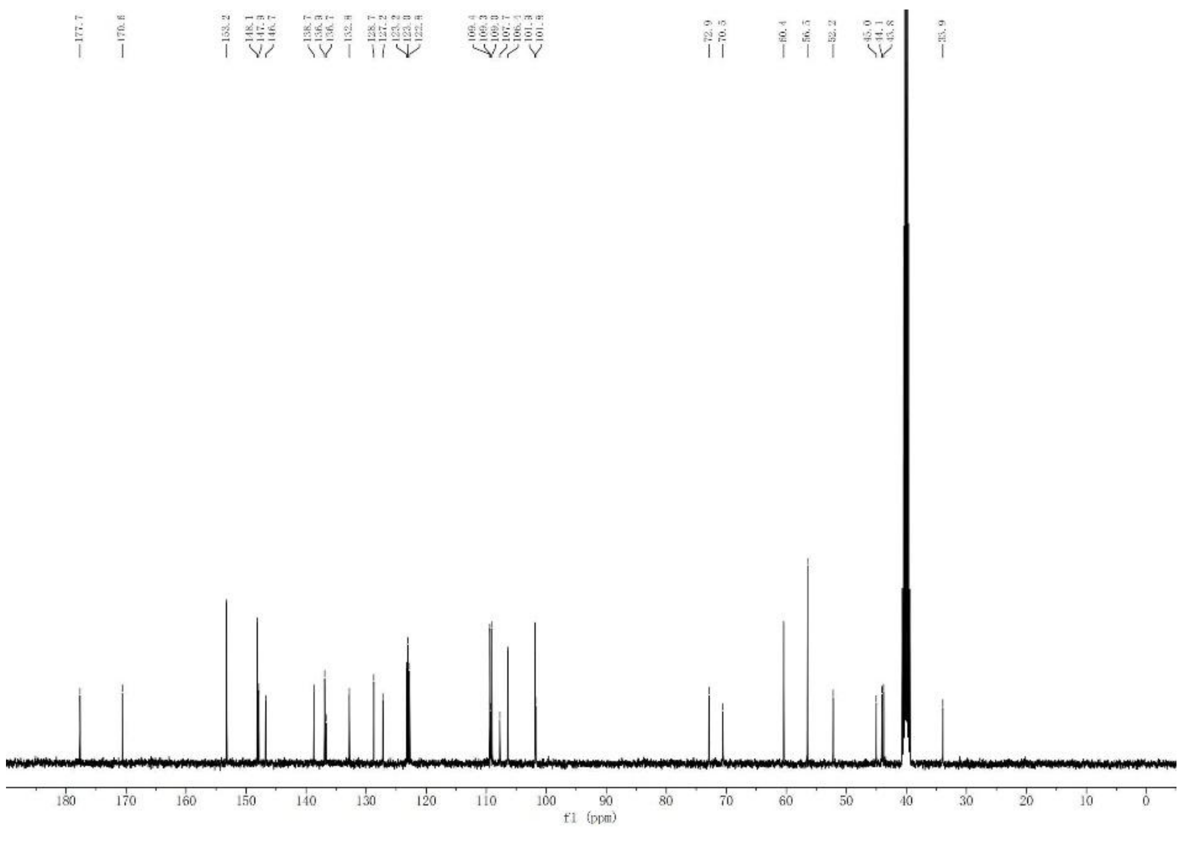

^{1}H NMR spectra (400 MHz, $CDCl_3$) of compound a13

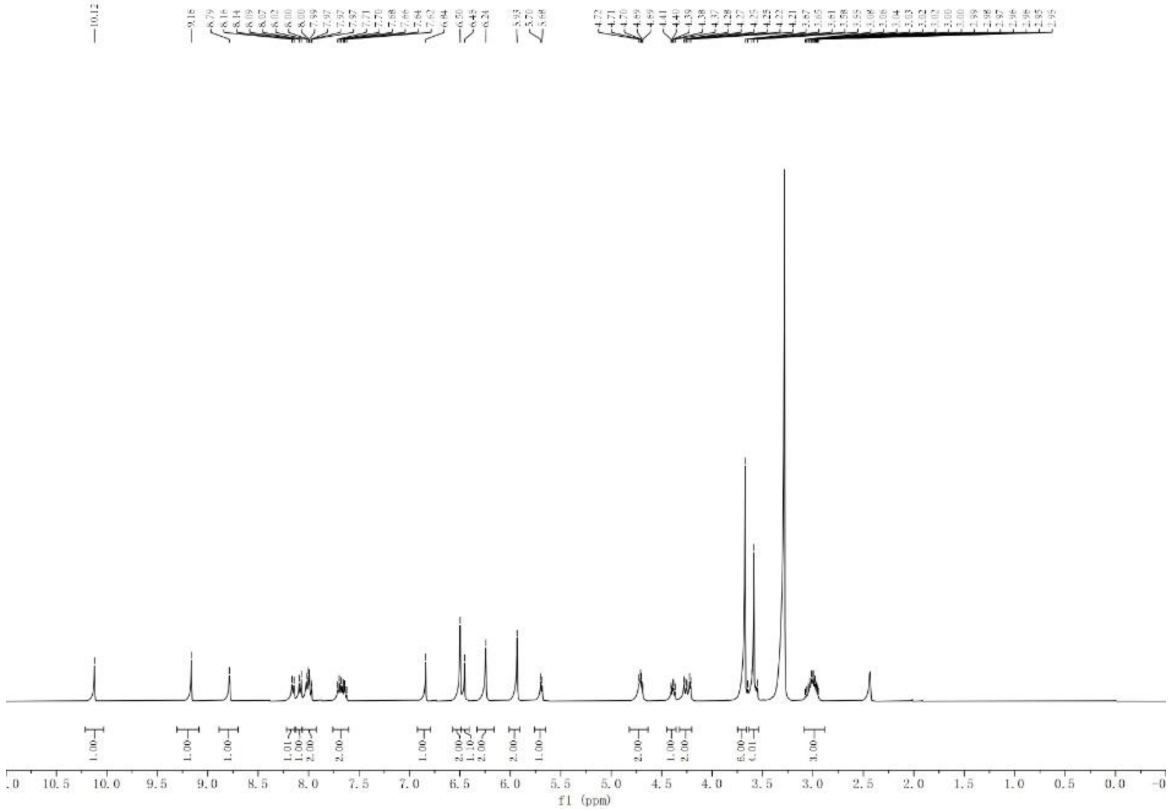

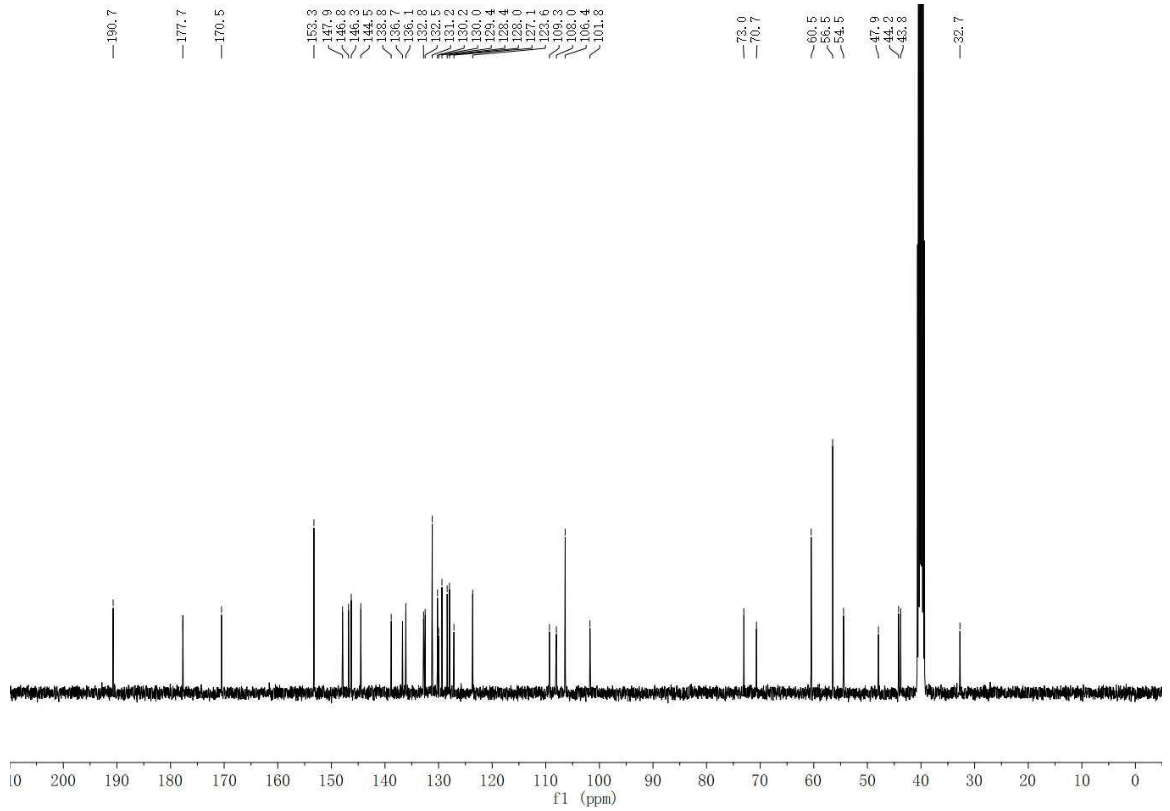

^{1}H NMR spectra (400 MHz, $CDCl_3$) of compound a14

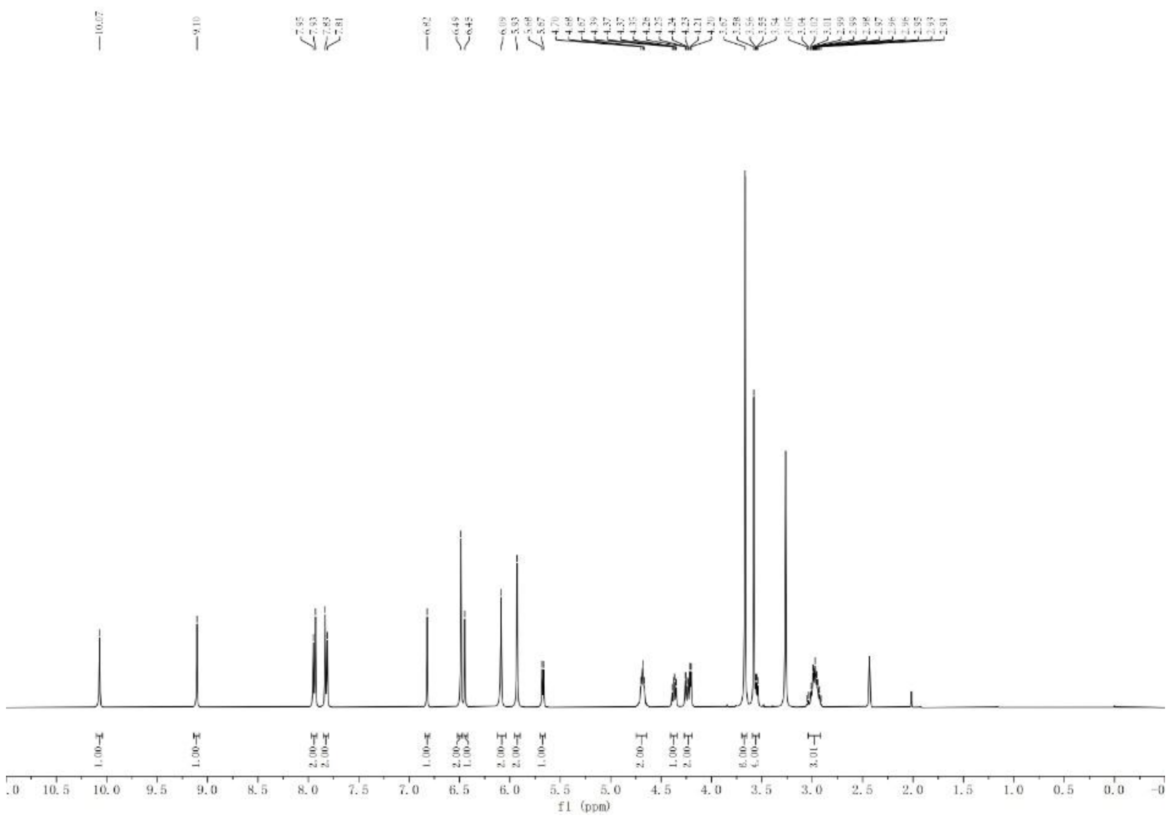

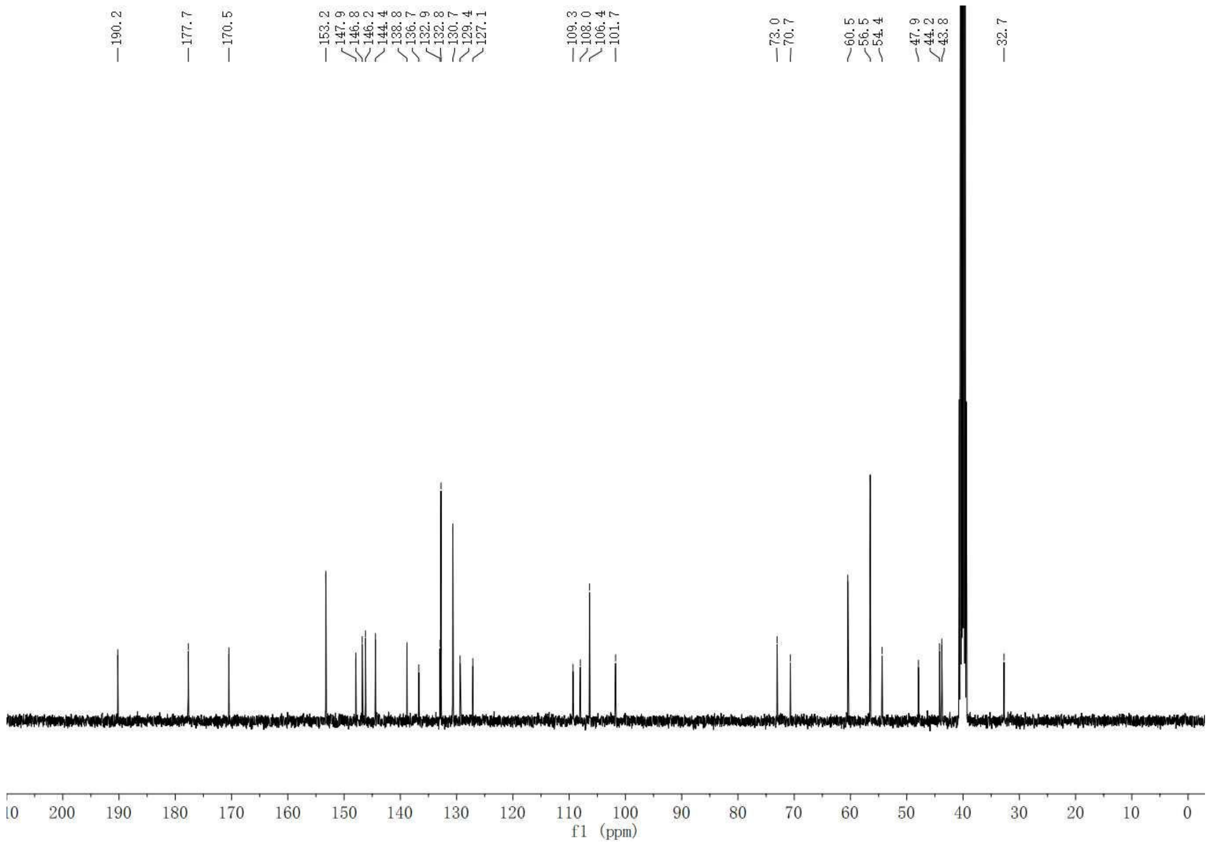

^{1}H NMR spectra (400 MHz, $CDCl_3$) of compound a15

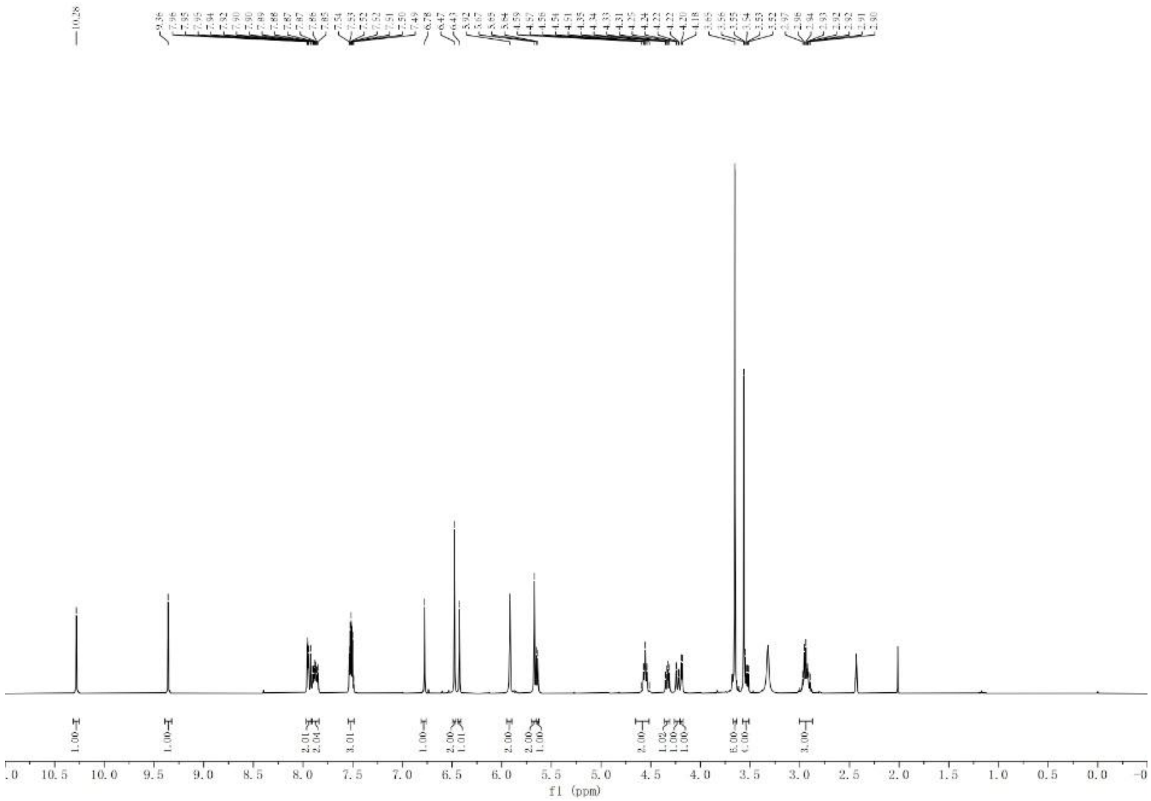

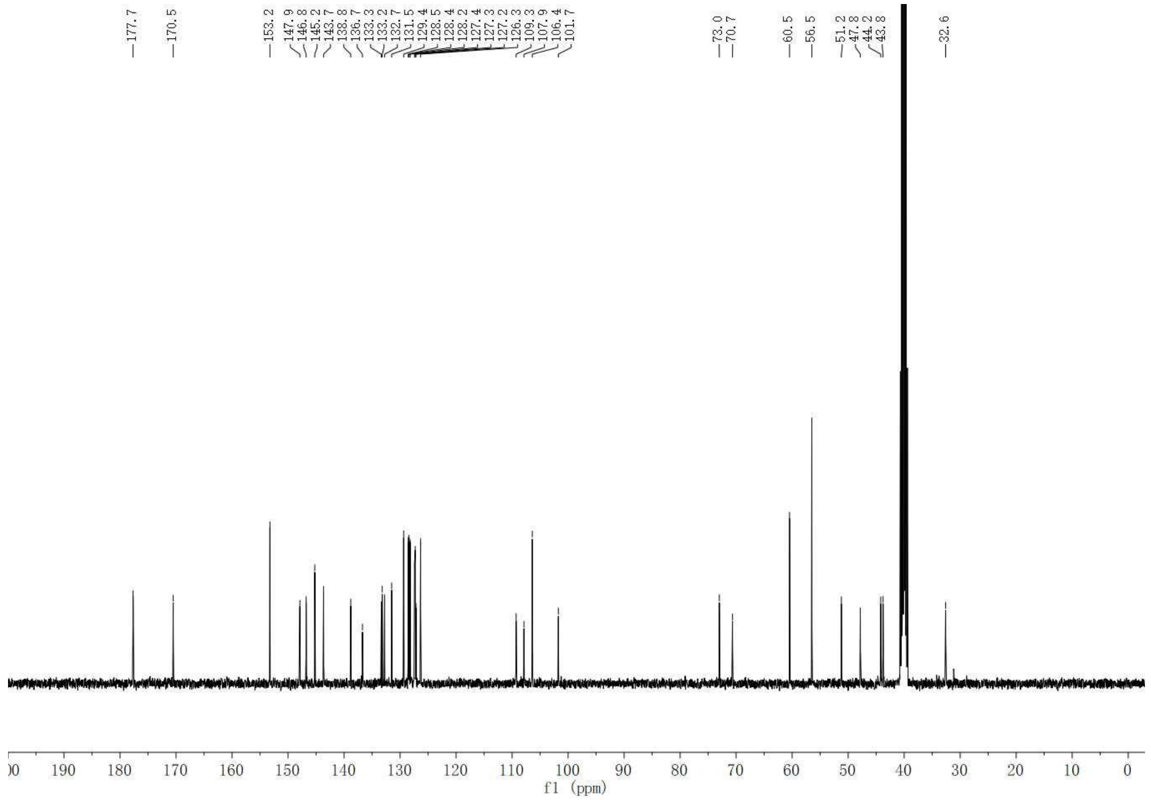

^{1}H NMR spectra (400 MHz, $CDCl_3$) of compound a16

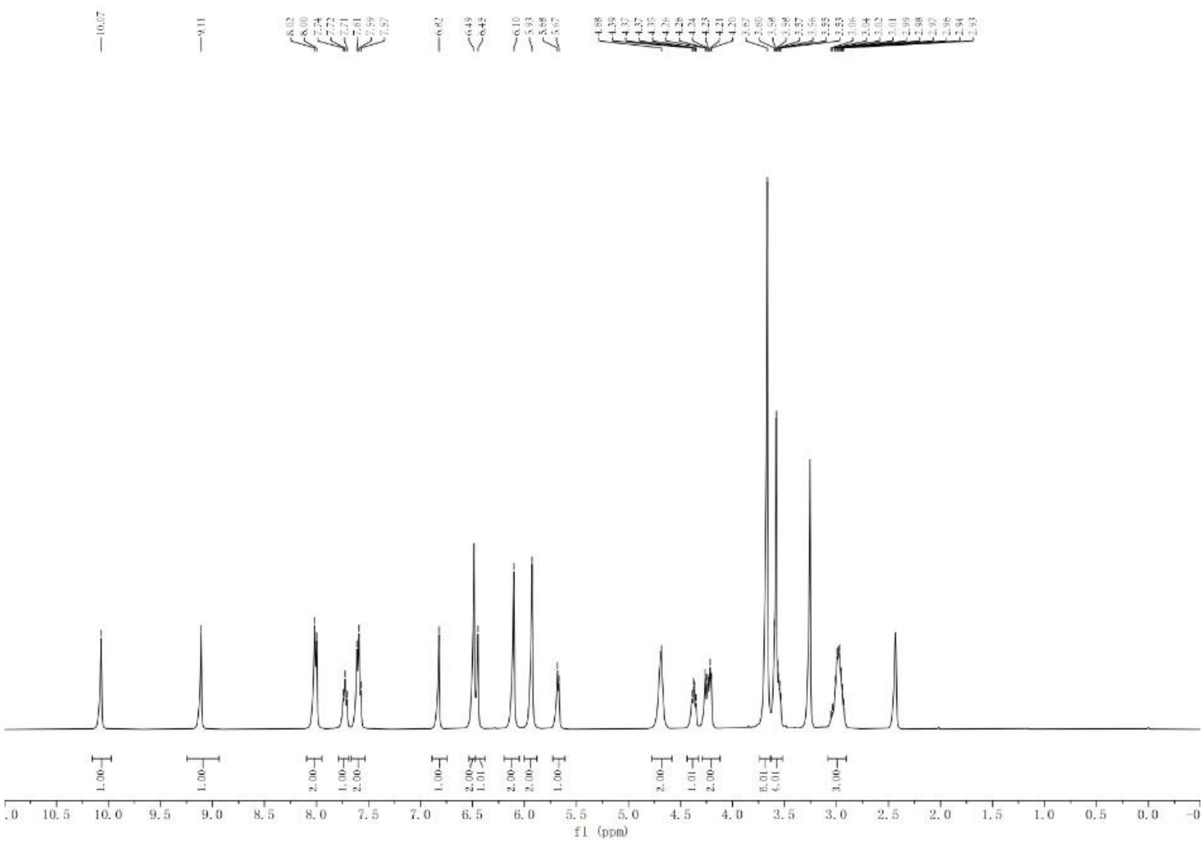

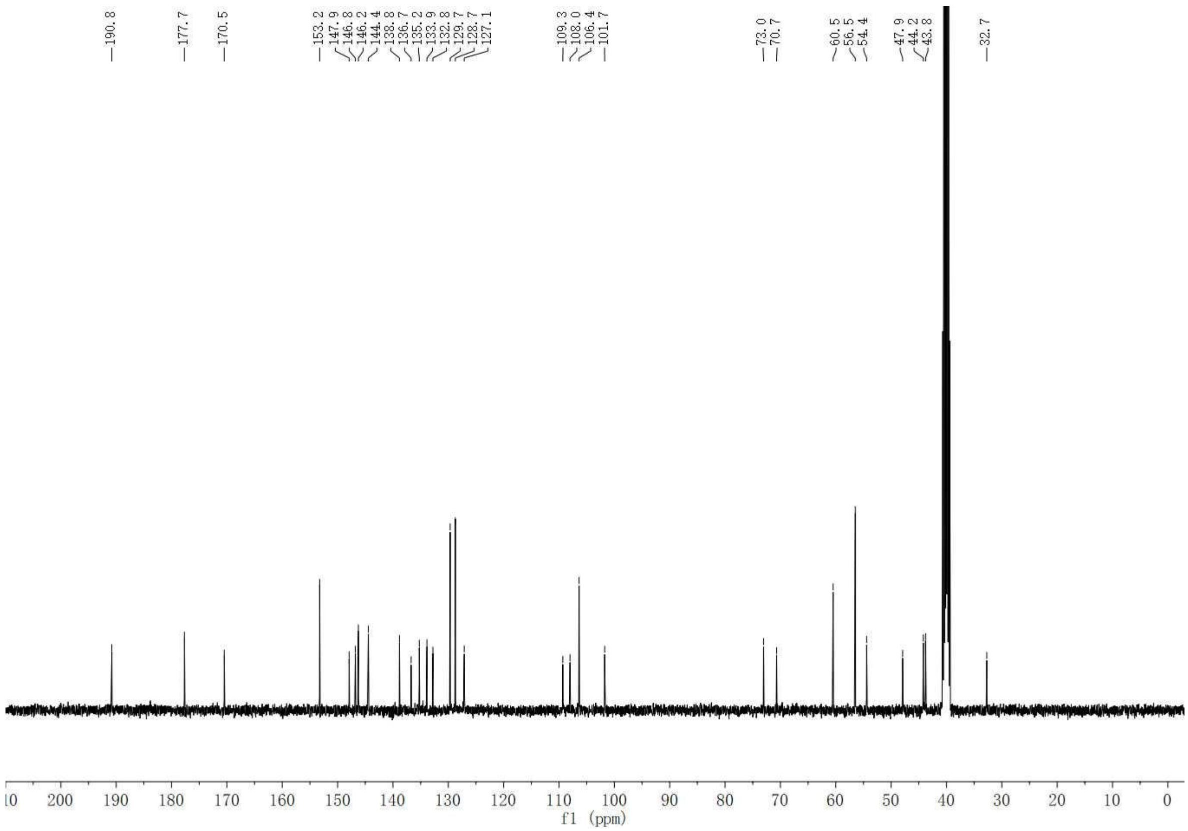

^{1}H NMR spectra (400 MHz, $CDCl_3$) of compound a17

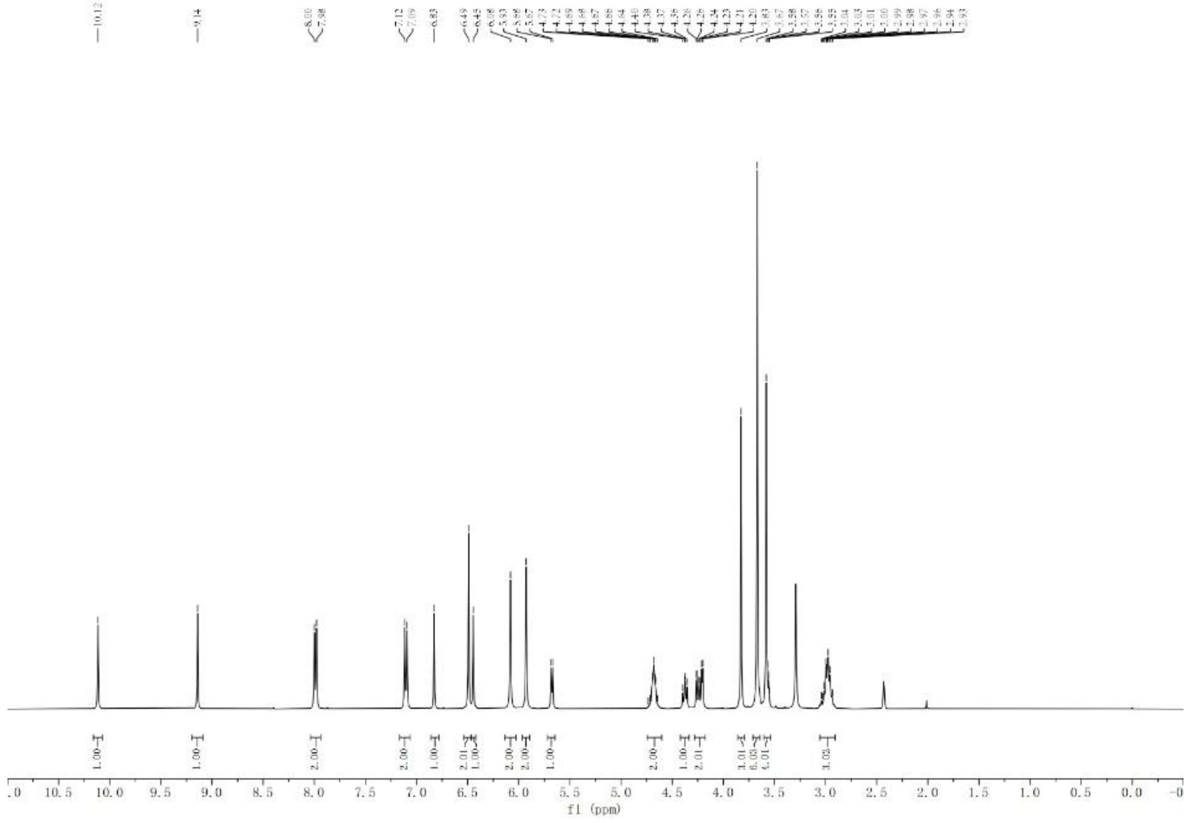

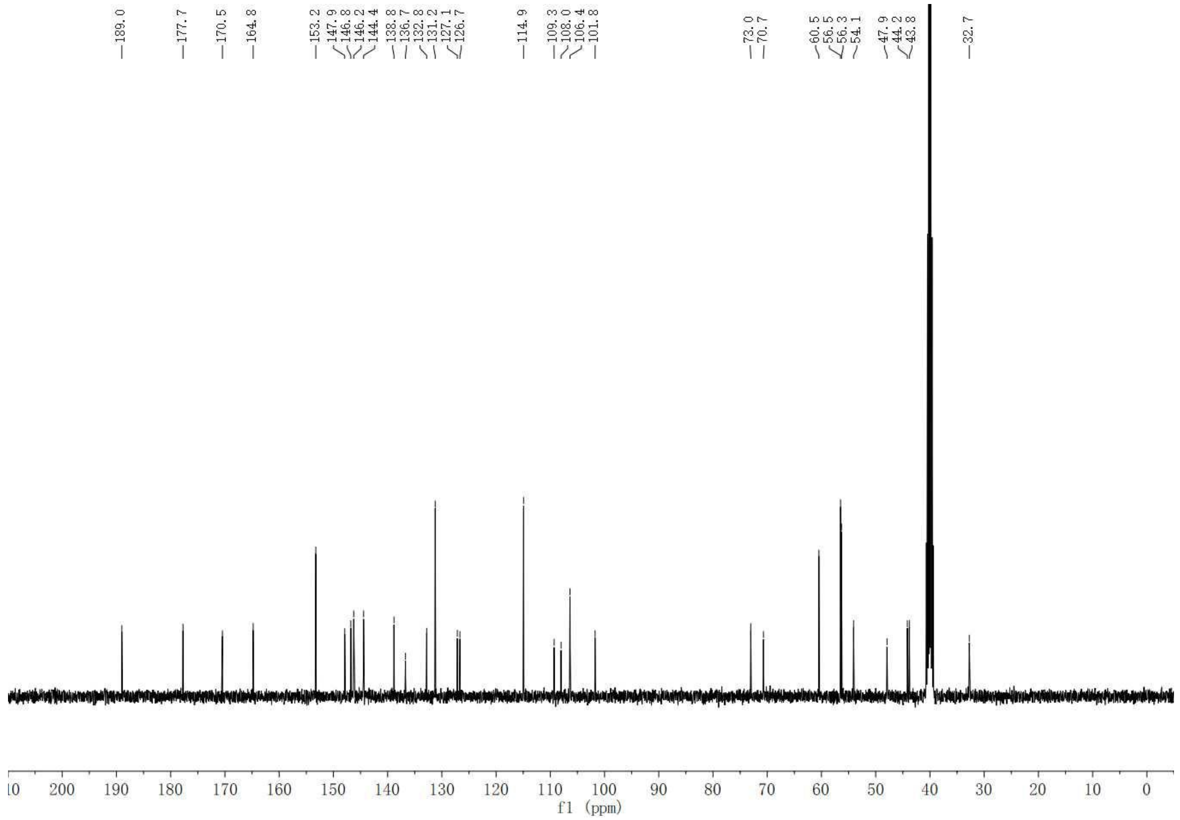

^{1}H NMR spectra (400 MHz, $CDCl_3$) of compound a18

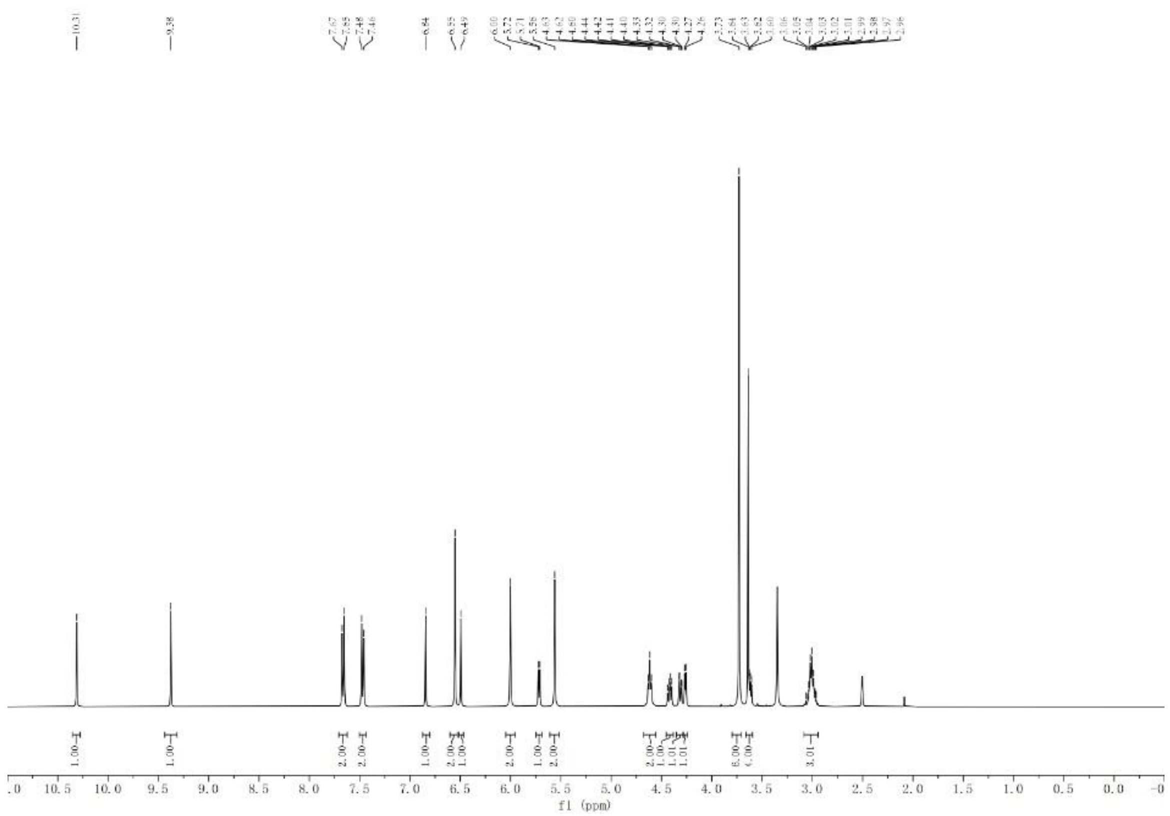

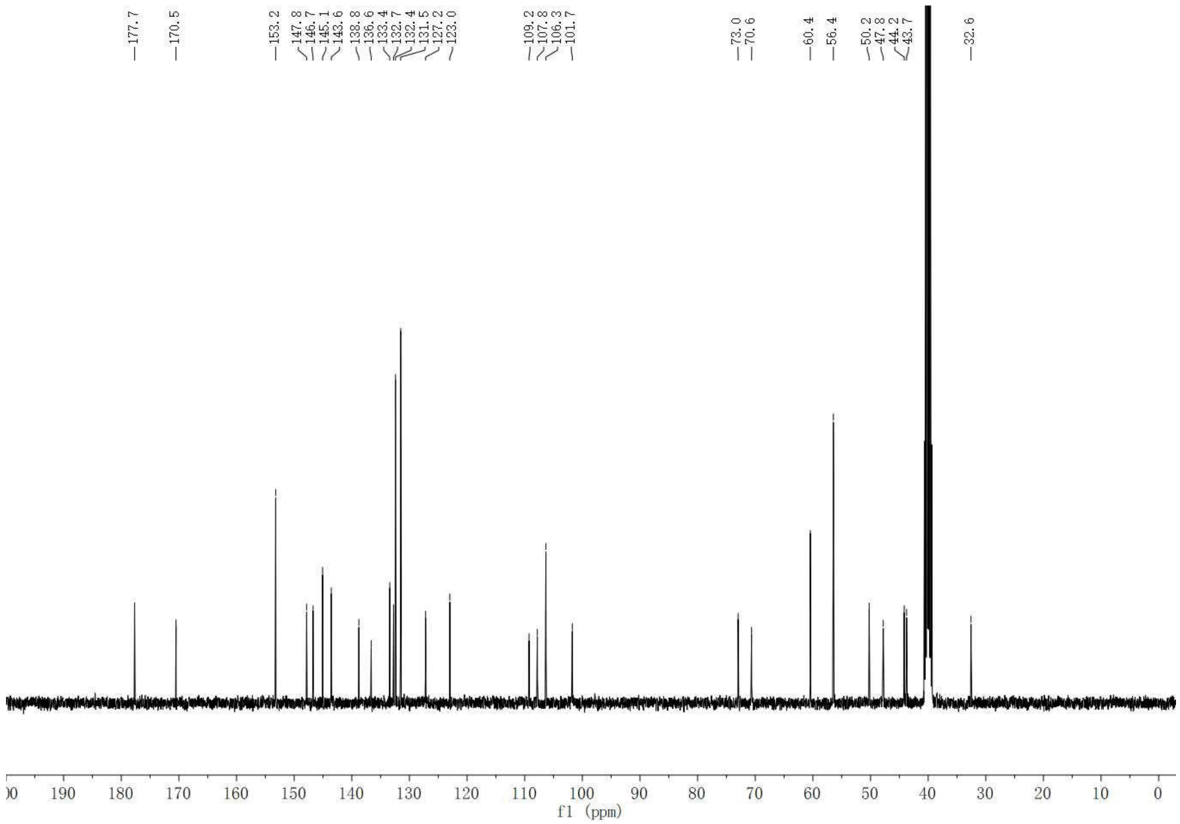

^{1}H NMR spectra (400 MHz, $CDCl_3$) of compound a19

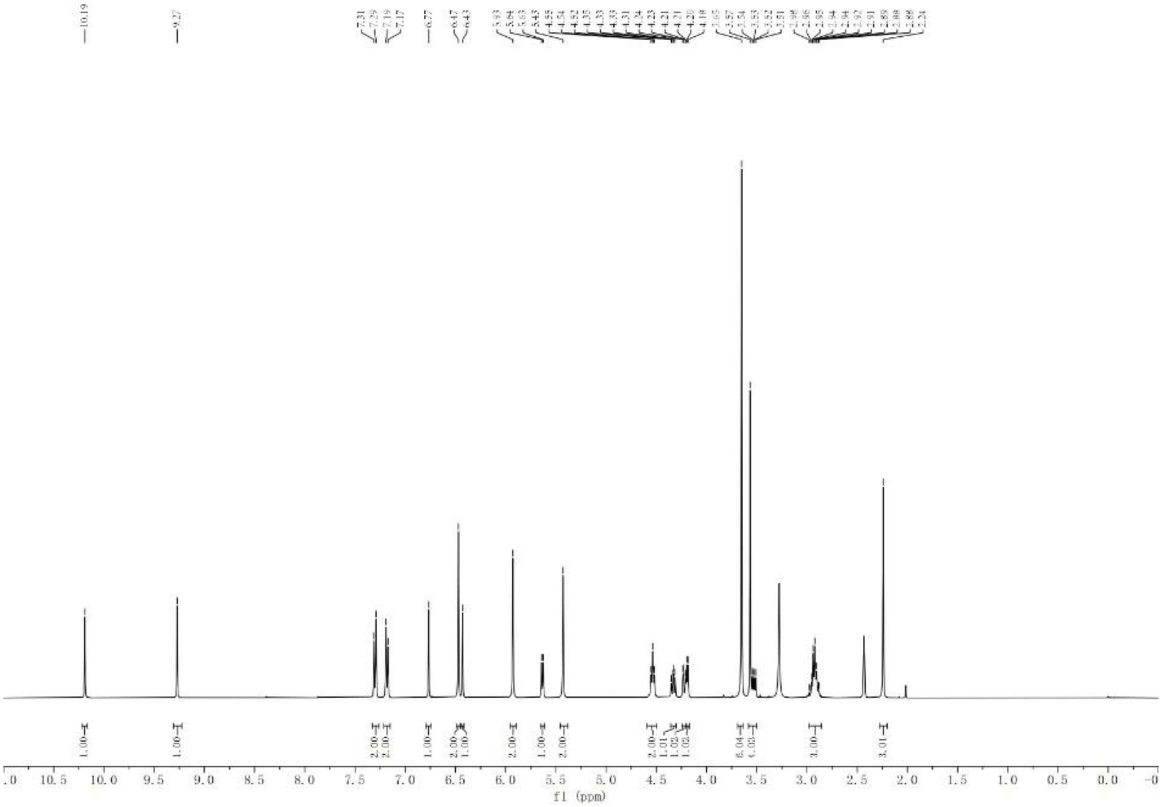

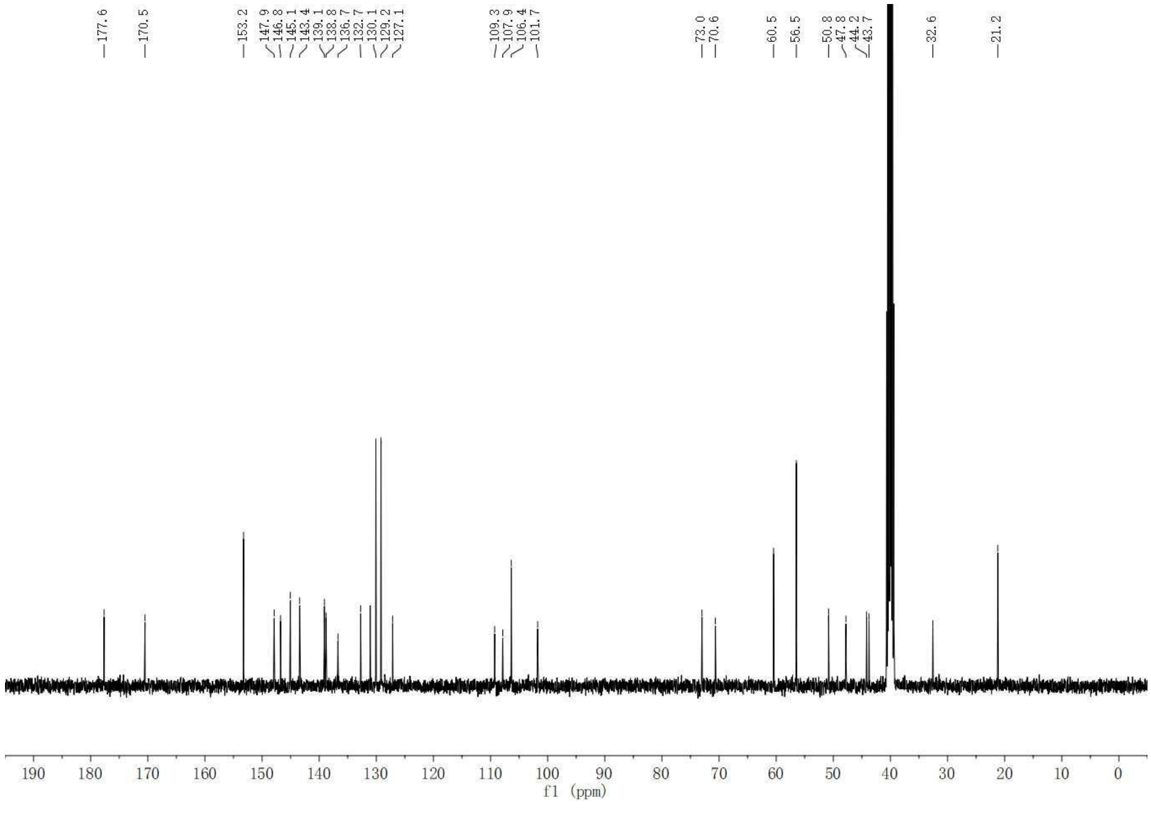

^{1}H NMR spectra (400 MHz, CDCl$_3$) of compound a20

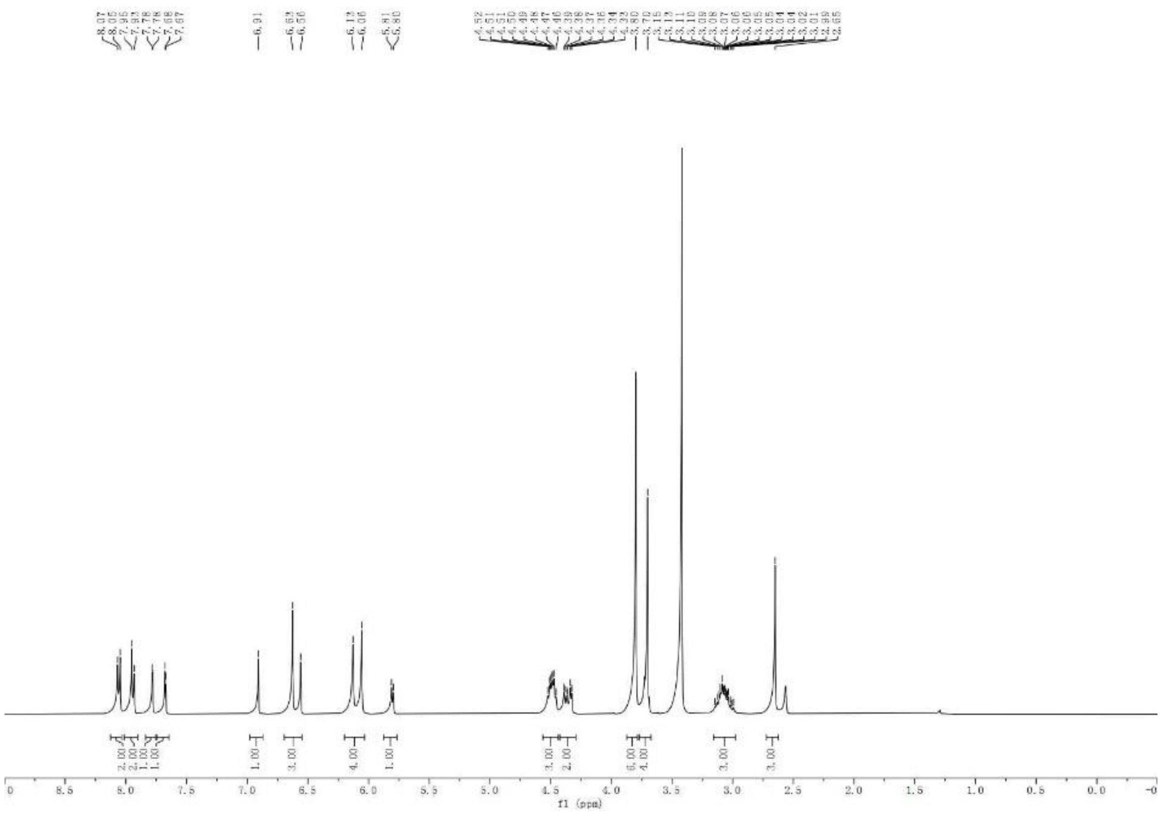

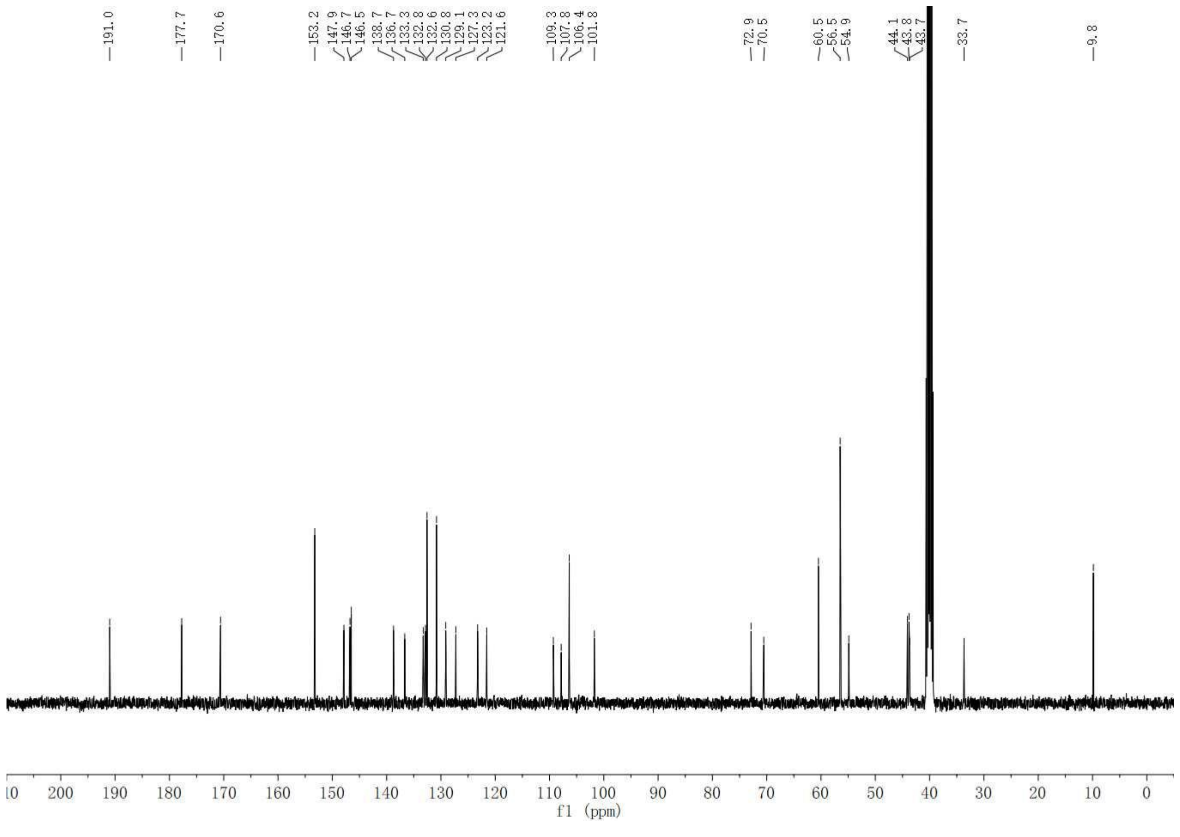

^{1}H NMR spectra (400 MHz, $CDCl_3$) of compound a21

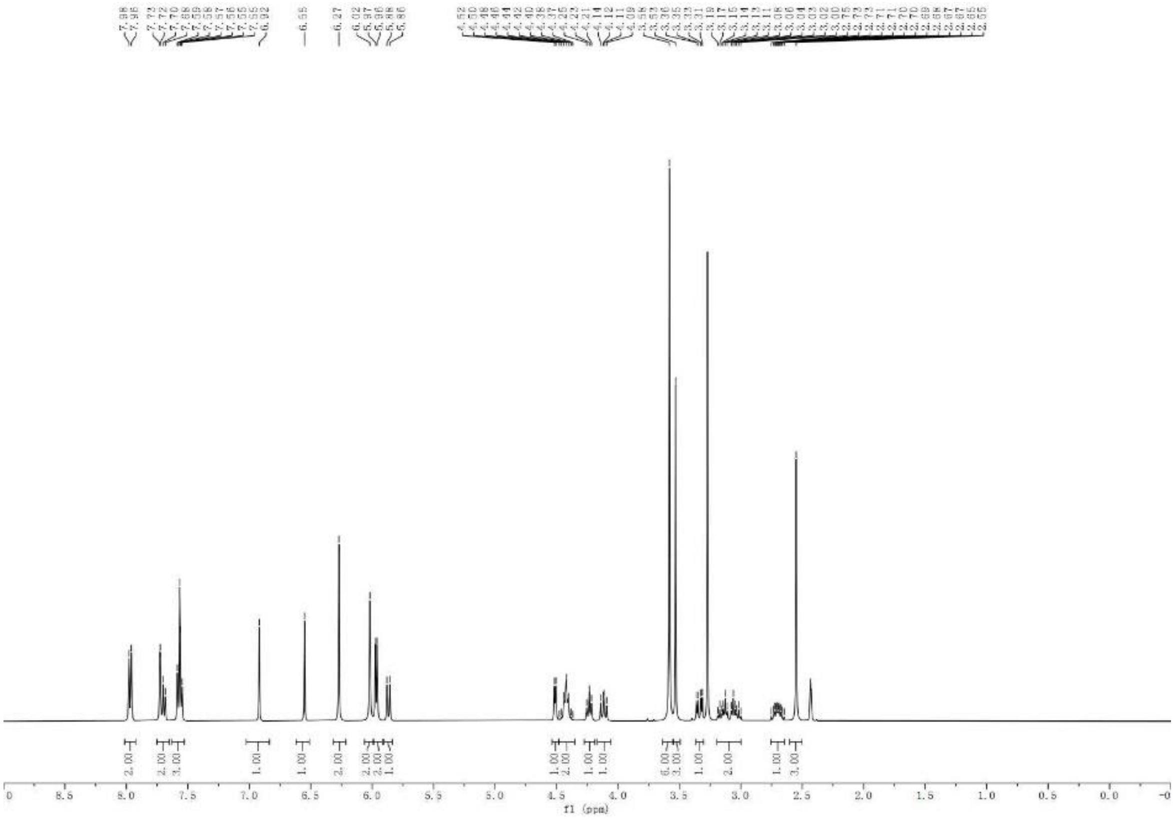

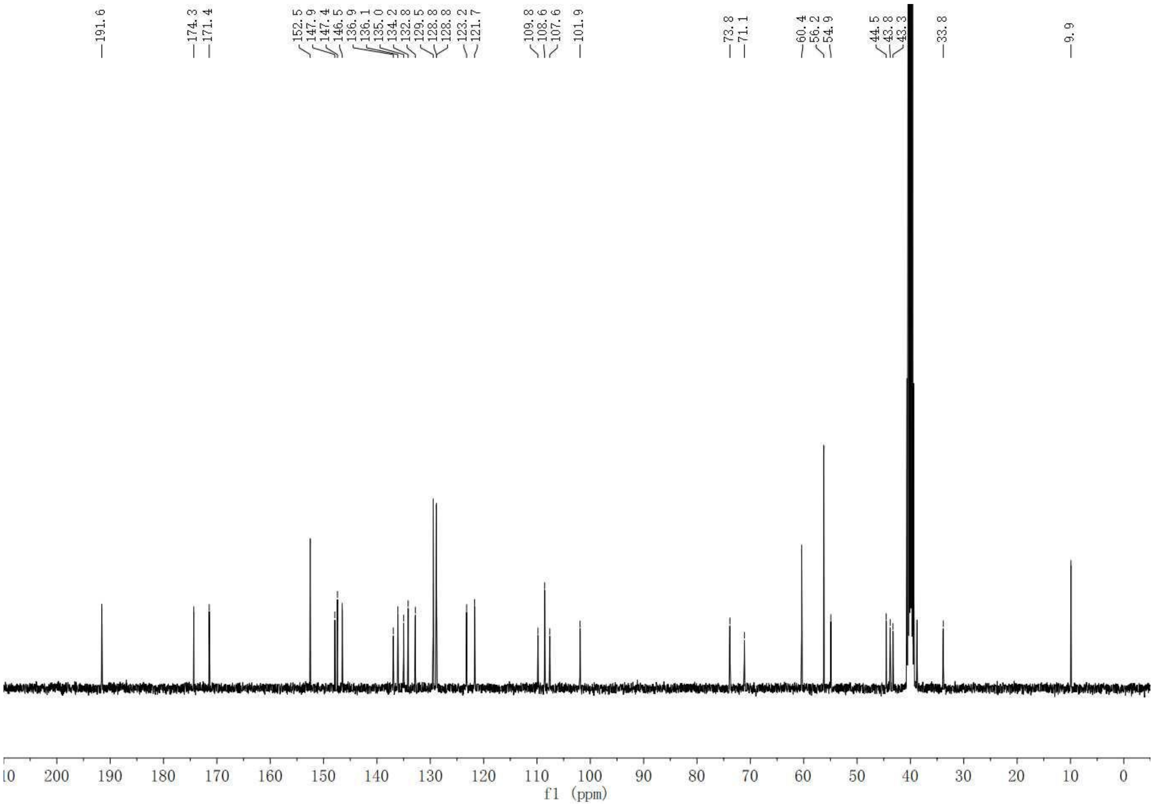

^{1}H NMR spectra (600 MHz, $CDCl_3$) of compound b1

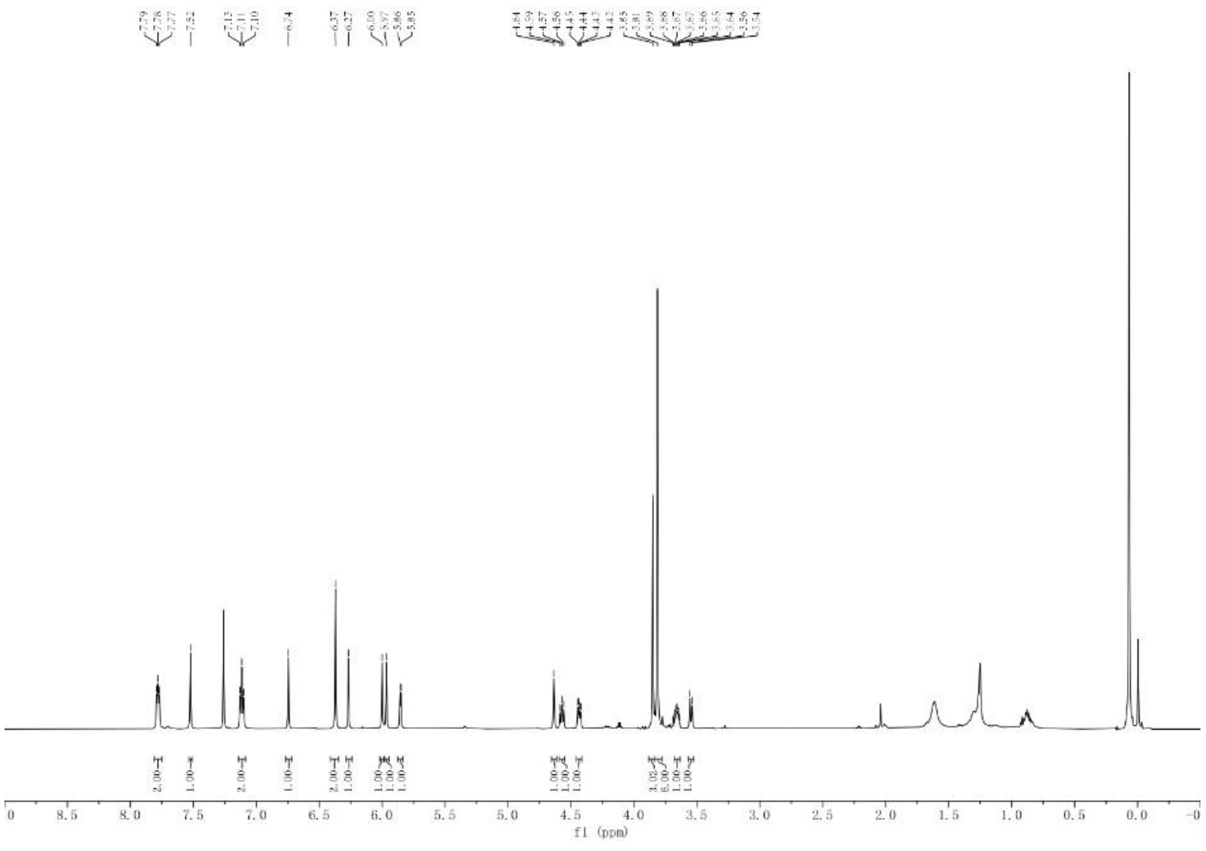

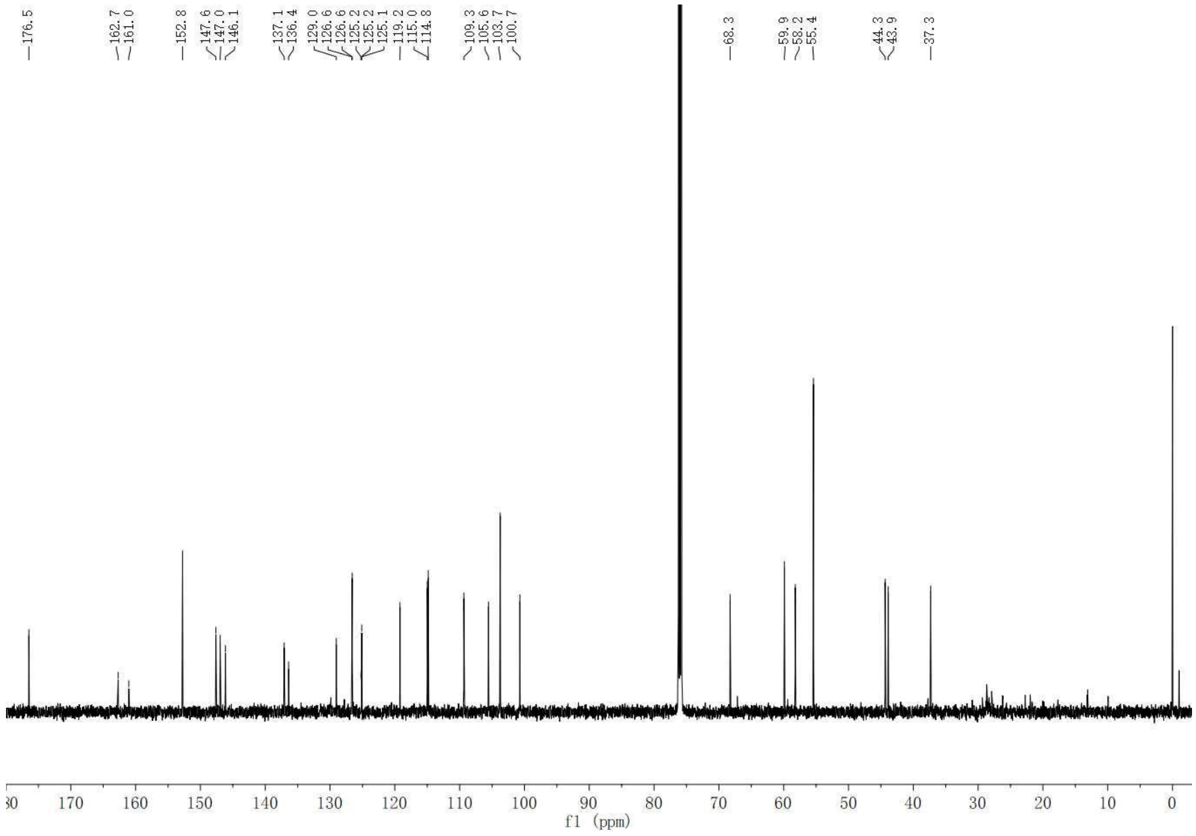

^{1}H NMR spectra (600 MHz, $CDCl_3$) of compound b2

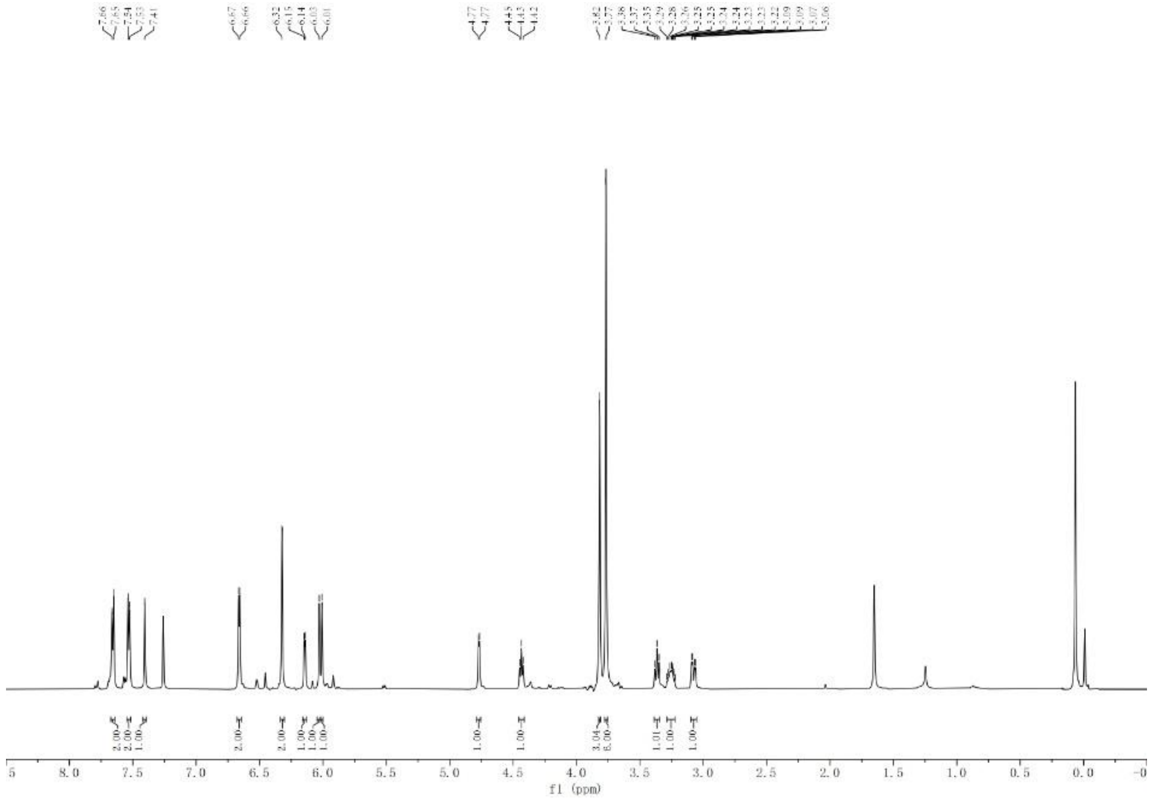

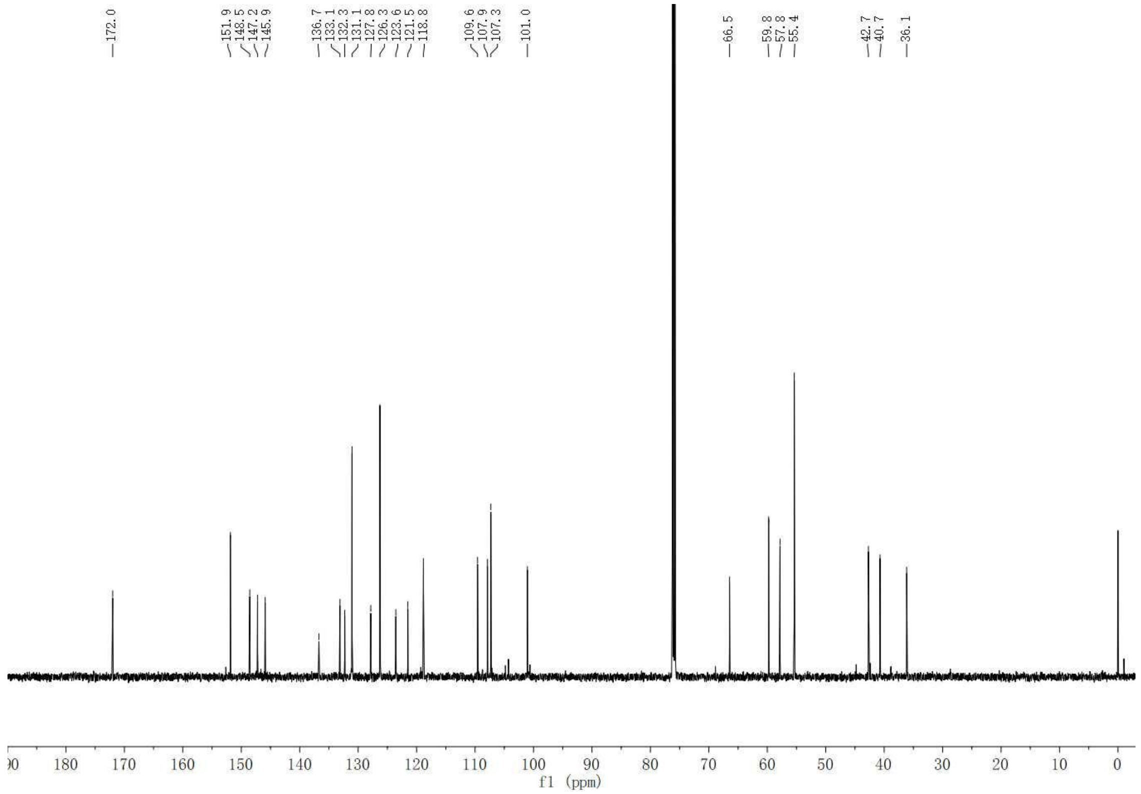

^{1}H NMR spectra (600 MHz, $CDCl_3$) of compound b3

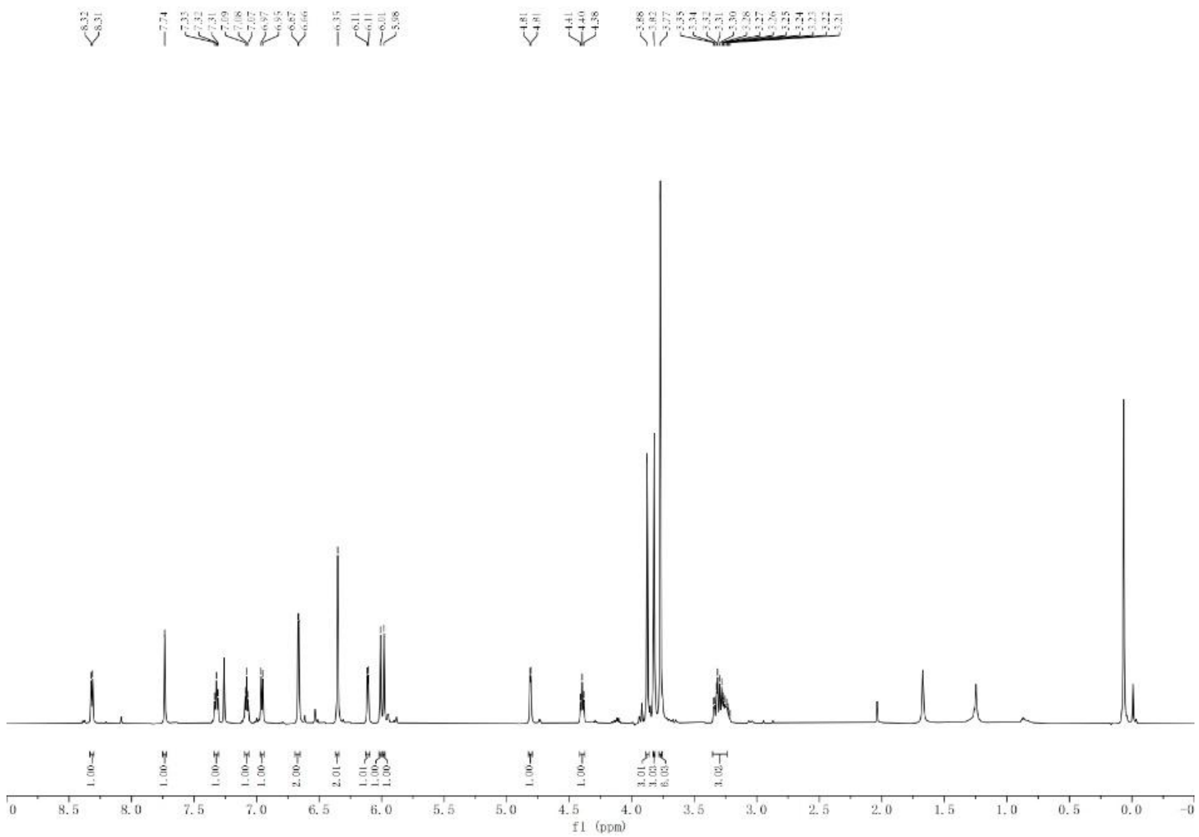

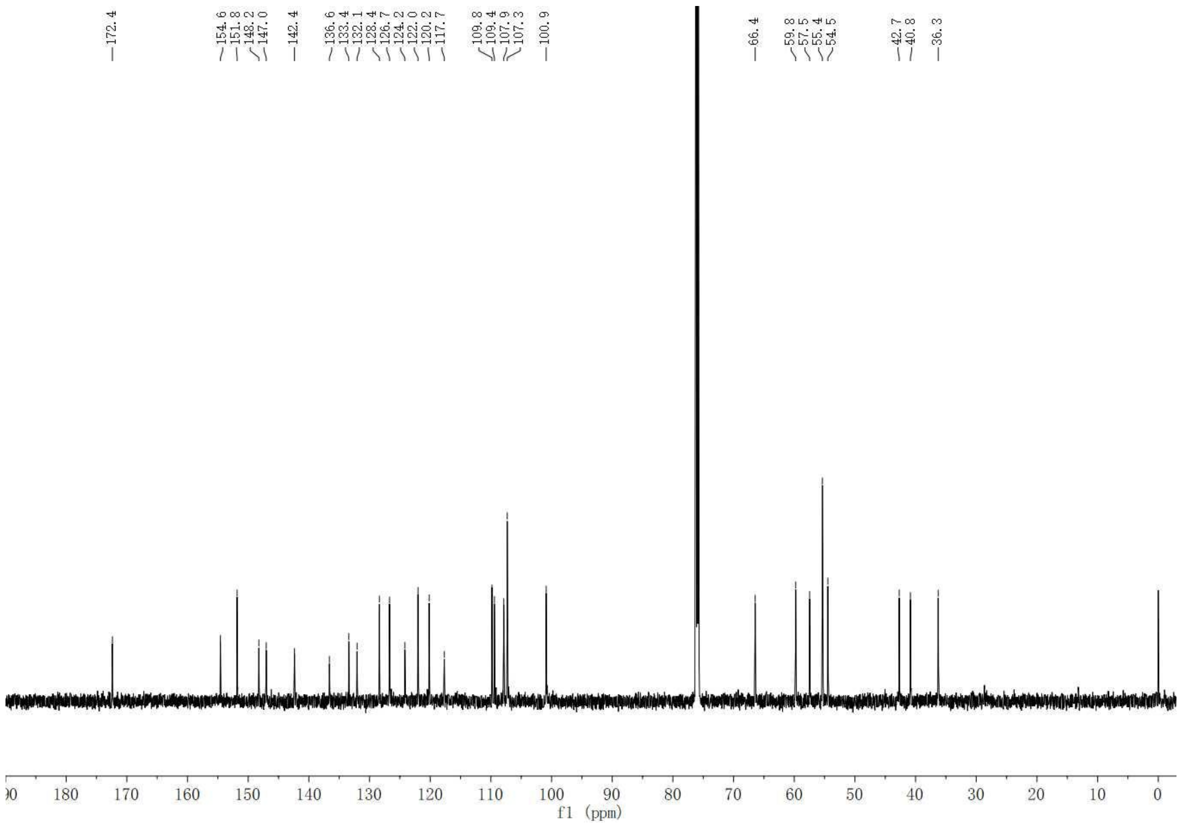

^{1}H NMR spectra (600 MHz, $CDCl_3$) of compound b4

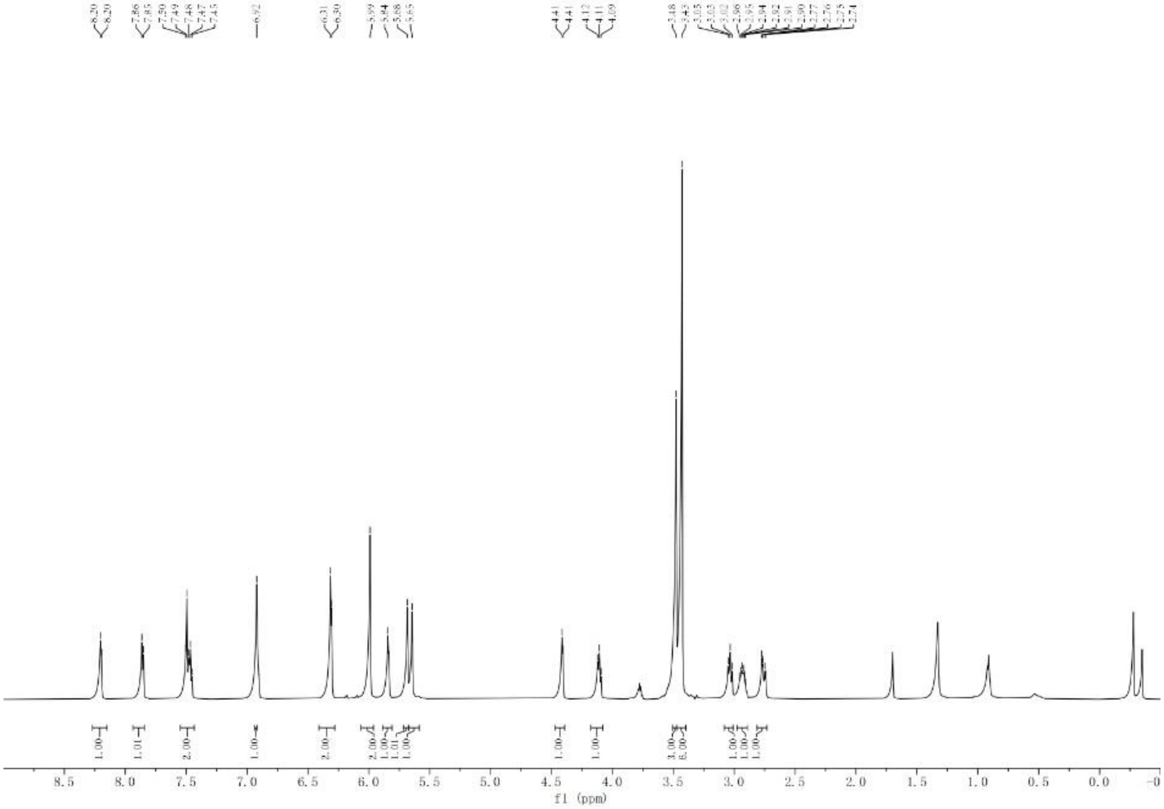

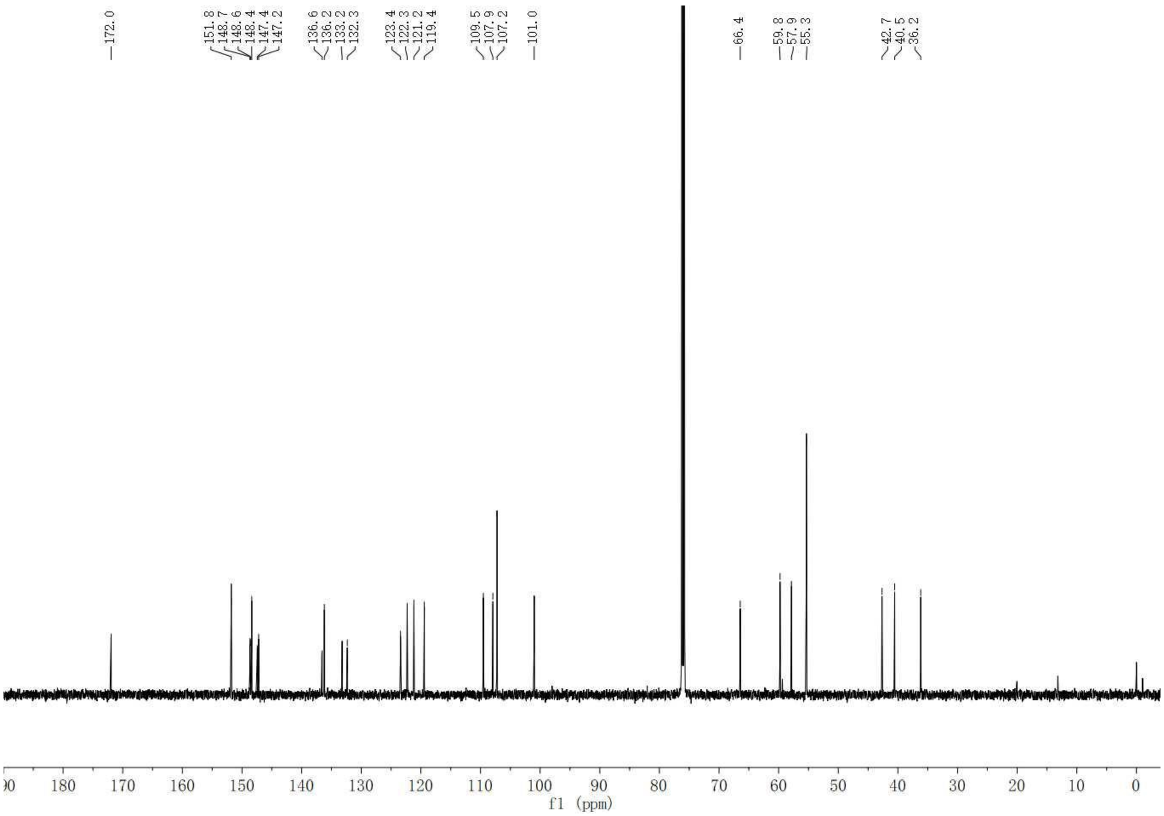

^{1}H NMR spectra (600 MHz, $CDCl_3$) of compound b5

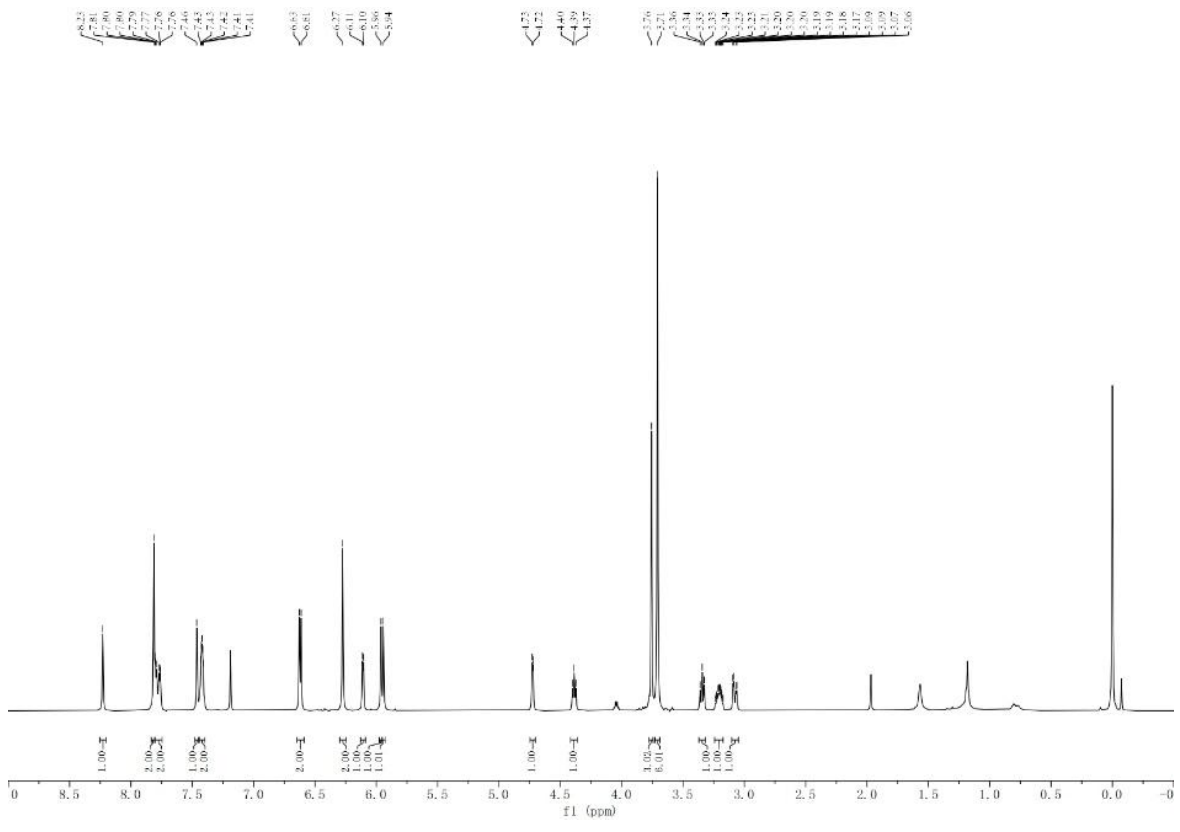

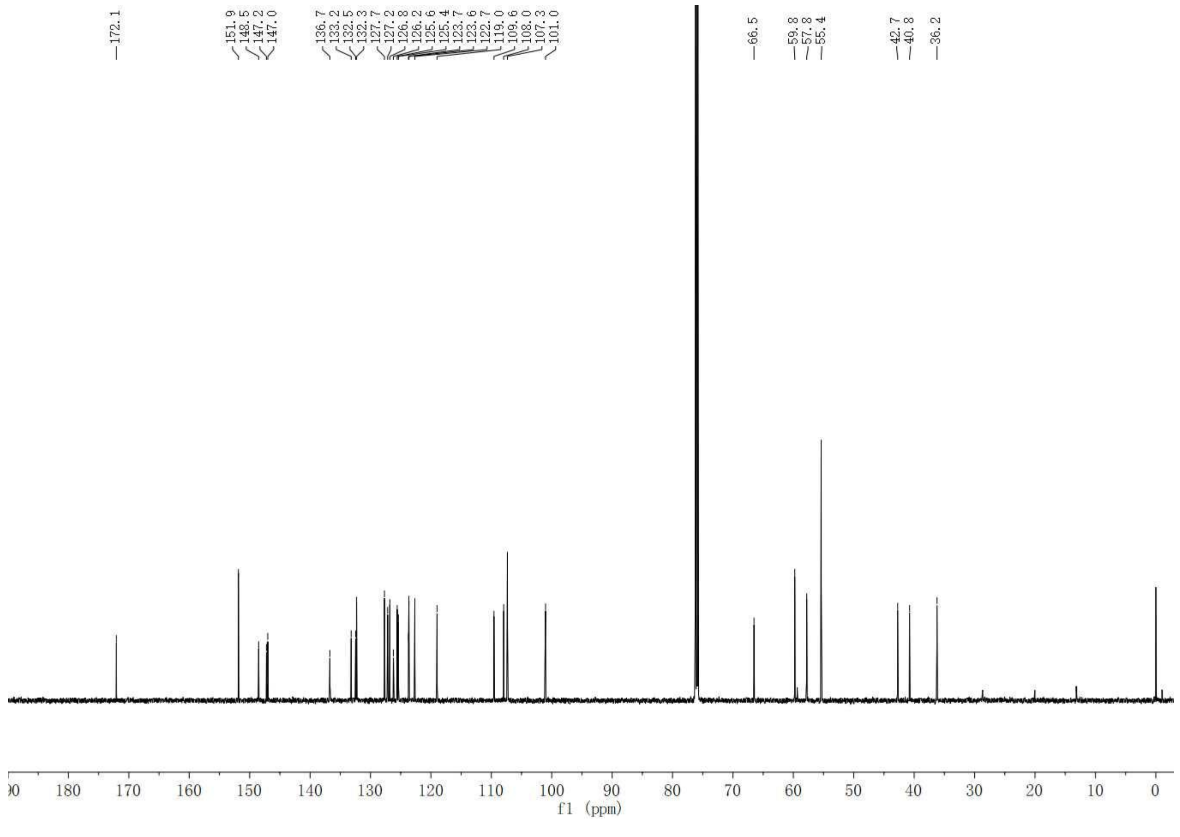

^{1}H NMR spectra (600 MHz, $CDCl_3$) of compound c1

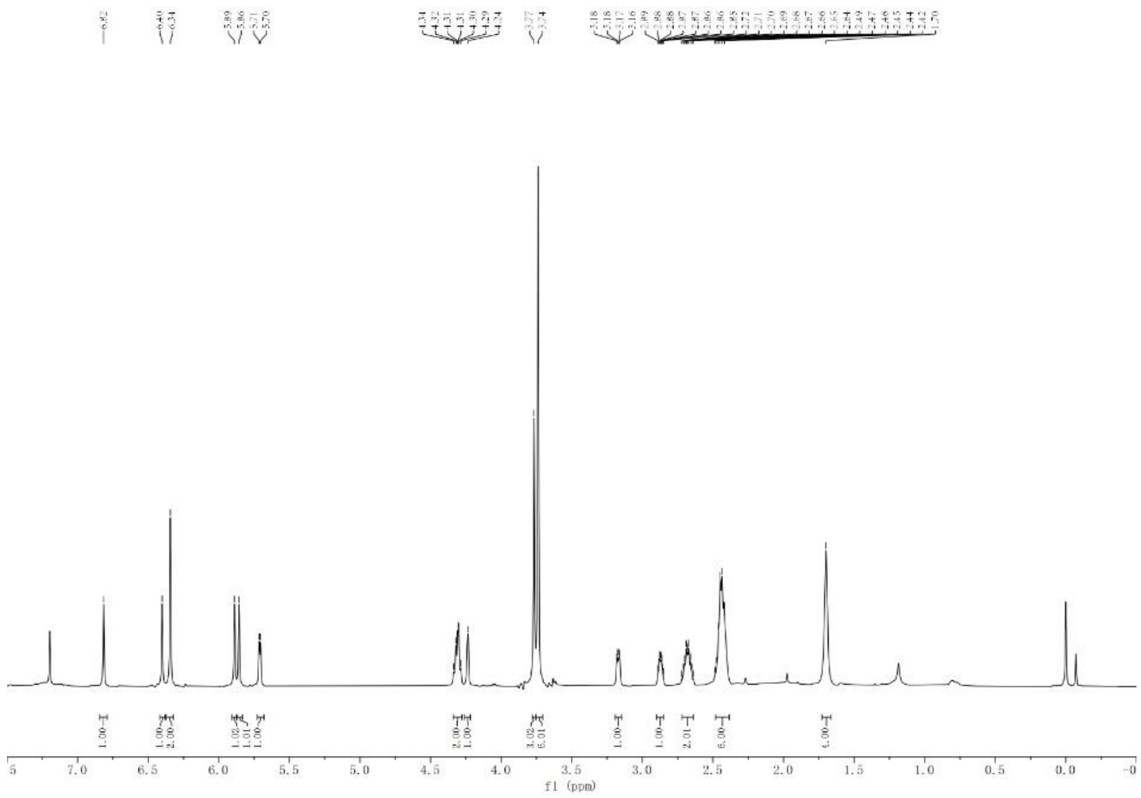

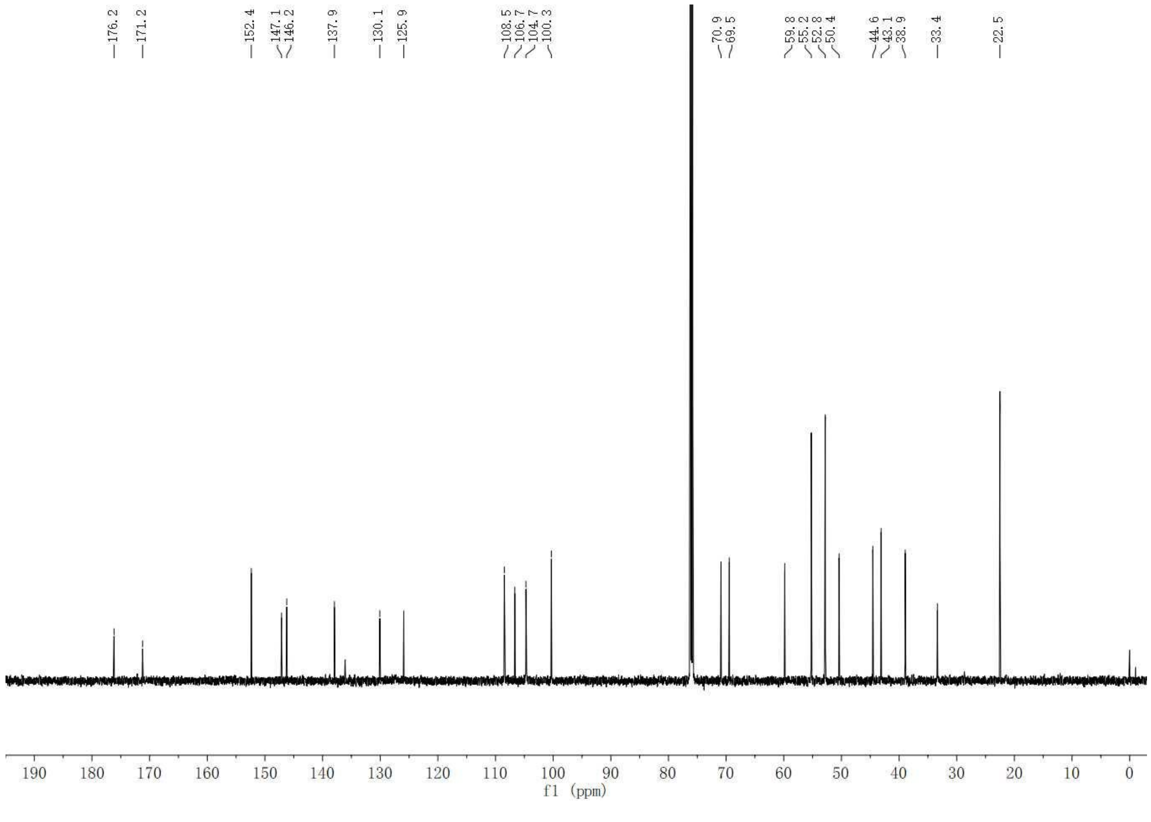

^{1}H NMR spectra (600 MHz, $CDCl_3$) of compound c2

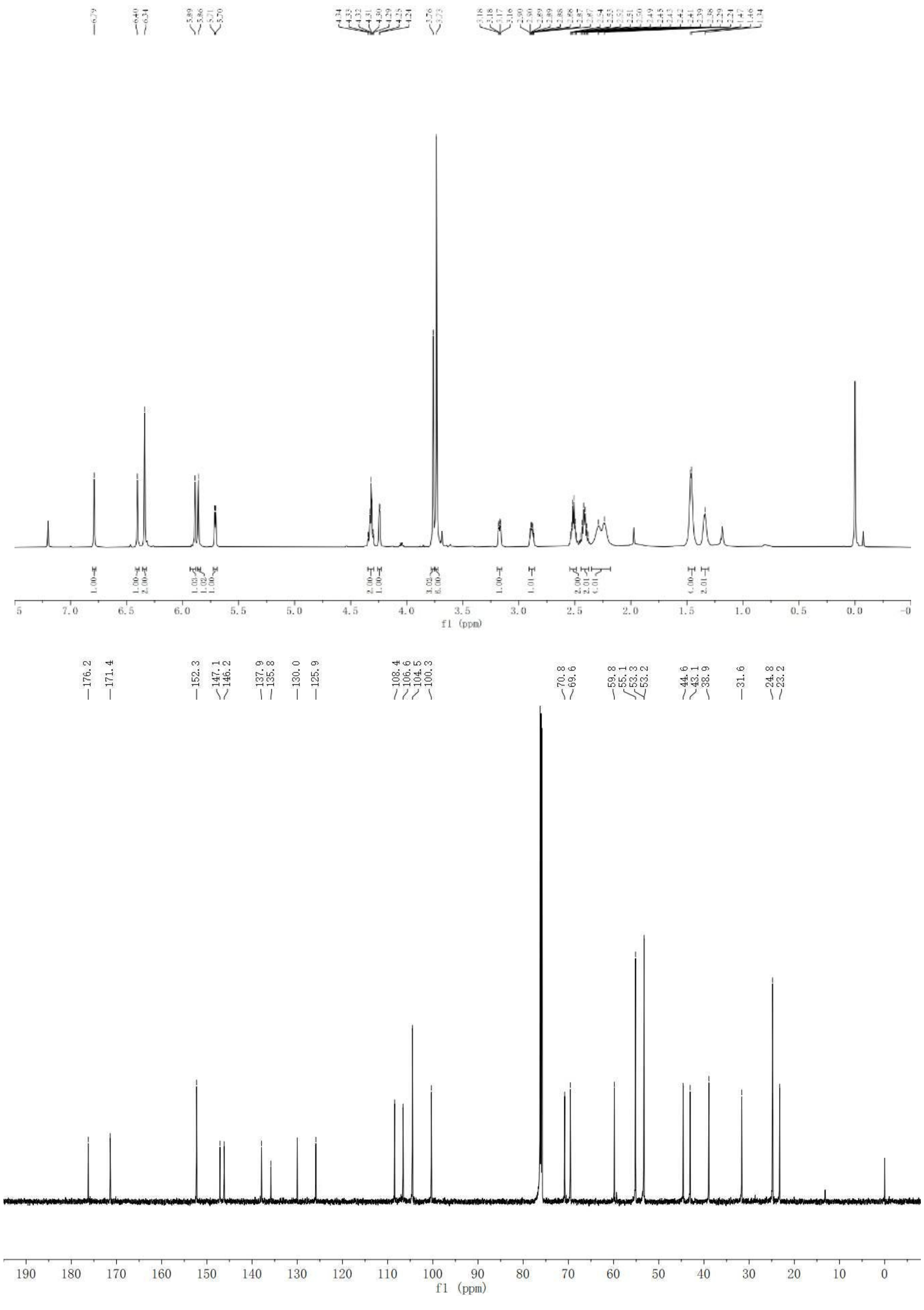

^{1}H NMR spectra (600 MHz, $CDCl_3$) of compound c3

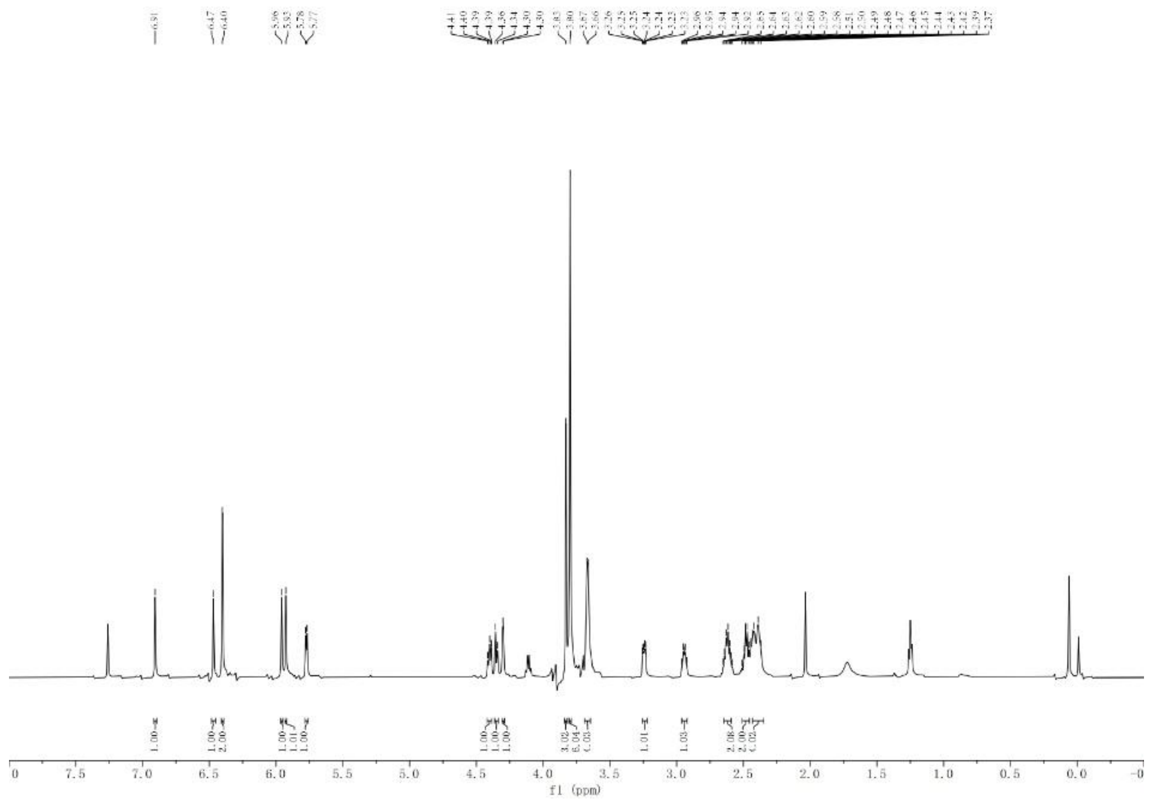
f1 (ppm)

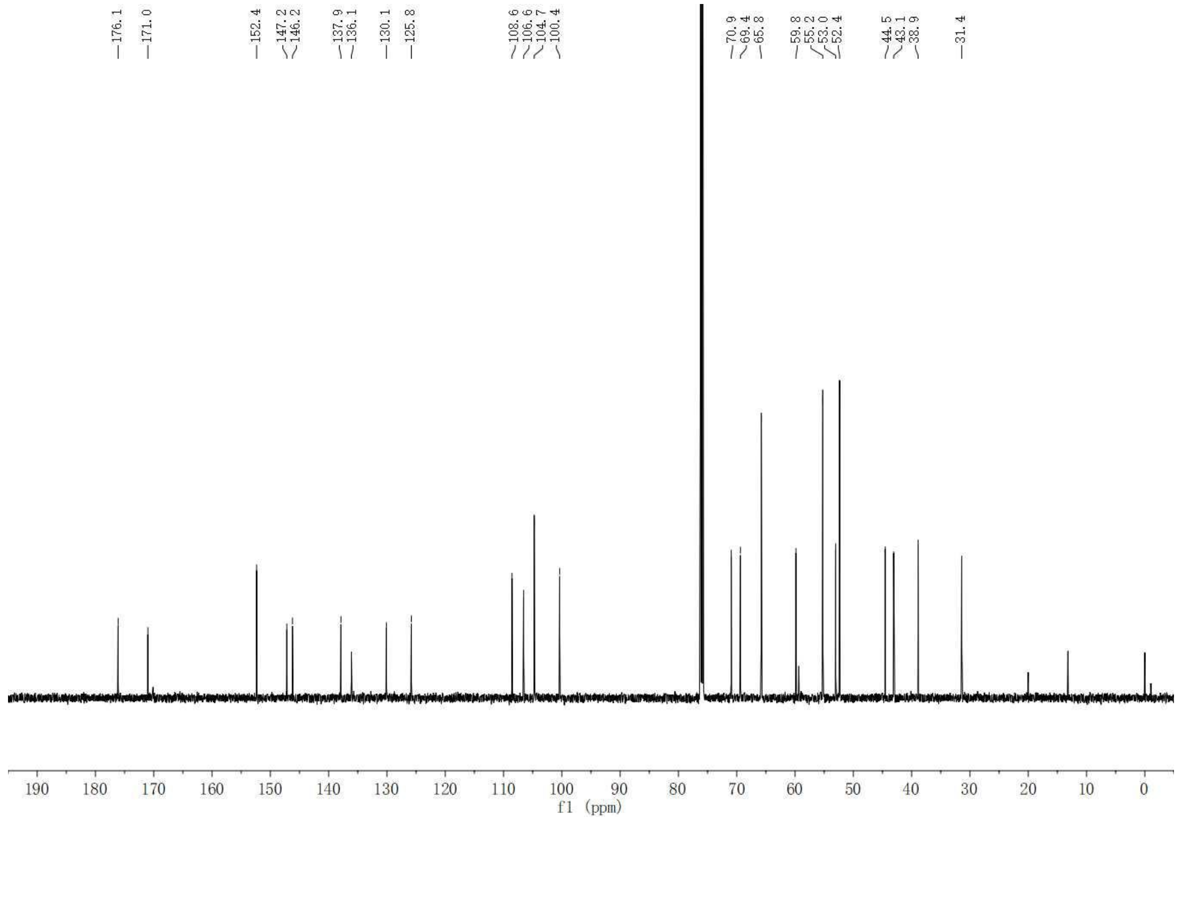
176.1
171.0
152.4
147.2
146.2
137.9
136.1
130.1
125.8
108.6
106.6
104.7
100.4
70.9
69.4
65.8
59.8
55.2
53.0
52.4
44.5
43.1
38.9
31.4
f1 (ppm)
